# Type II Alveolar Epithelial Cells Promote Sepsis‐Induced Immunosuppression in Alveolar Macrophages via Exosomal lncRNA Rmrp Release

**DOI:** 10.1002/advs.202500376

**Published:** 2025-11-03

**Authors:** Chengxi Liu, Weixia Xuan, Song Cao, Huayun Jia, Qian Wu, Xiaowu Tan, Qijie Wang, Xiaojun Li, Lisha Ding, Yaru Xiong, Meiyun Zhao, Longcheng Zheng, Yunzhu Xi, Jianhua Tan, Rong Li, Xulong Zhang, Wenjie Liu, Xu Wu

**Affiliations:** ^1^ Department of Anesthesiology Anesthesiology and Pain Research Institute the Second Affiliated Hospital University of South China Hengyang Hunan 421001 China; ^2^ Department of Respiratory and Critical Care Medicine Henan Provincial People's Hospital People's Hospital of Zhengzhou University Zhengzhou Henan 450003 China; ^3^ Department of Pain Medicine The Tenth Affiliated Hospital, Southern Medical University (Dongguan People's Hospital) Dongguan Guangdong 523000 China; ^4^ Biosafety Level 3 Laboratory Hunan Province Center for Disease Control and Prevention Changsha Hunan 410005 China; ^5^ Institute of Human Virology Zhongshan School of Medicine and Key Laboratory of Tropical Disease Control of Ministry of Education Sun Yat‐sen University Guangzhou 510275 China; ^6^ Pulmonary and Critical Care Medicine the Second Affiliated Hospital, University of South China. Hengyang Medical School University of South China Hengyang Hunan 421001 China; ^7^ Department‌ of Infectious Disease The Central Hospital of Shaoyang Shaoyang Hunan 422000 China; ^8^ Department of lmmunology School of Basic Medical Sciences Capital Medical University Beijing China

**Keywords:** alveolar macrophages, exosomal Rmrp, glycolysis, secondary pneumonia, sepsis‐induced immunosuppression

## Abstract

Secondary pneumonia, a common complication of sepsis‐induced immunosuppression (SII), is closely linked to alveolar macrophage (AM) dysfunction primarily due to impaired glycolytic activity. However, the underlying molecular mechanisms remain unclear. In this study, it is found that exosomal RNA component of the mitochondrial RNA processing endoribonuclease (Rmrp), derived from type II alveolar epithelial cells (AEC‐IIs), drives glycolytic defects and immune tolerance in AMs following cecal ligation and puncture (CLP) sepsis. Targeted depletion of Rmrp in either AEC‐IIs or AMs alleviated SII and secondary pneumonia induced by *Pseudomonas aeruginosa* infection 48 h post CLP. Mechanistically, Rmrp interacts with and inhibits the ubiquitination and degradation of the RNA‐binding protein zinc finger protein 36 (ZFP36). This results in ZFP36 upregulation, subsequently accelerating the decay of 6‐phosphofructo‐2‐kinase/fructose‐2,6‐bisphosphatase 3 (Pfkfb3*)* mRNA by binding to its AU‐rich elements in the 3′ untranslated region. The degradation of *Pfkfb3* mRNA leads to impaired glycolysis and suppresses immune responses in AMs after sepsis. Additionally, it is found that exosomal Rmrp levels are correlated with AM immune tolerance and the prognosis of patients with sepsis. These findings highlight the critical role of AEC‐II‐derived exosomal Rmrp in the pathogenesis of SII and secondary pneumonia. Importantly, the study suggests that exosomal Rmrp may serve as a biomarker for predicting and managing SII in clinical settings.

## Graphical abstract

The S100A4/STAT3 axis promotes Rmrp transcription in AEC‐IIs and Rmrp expression in AEC‐II‐derived exosomes. Upon entry into AMs, Rmrp binds to and blocks the ubiquitination and degradation of ZFP36. Increased ZFP36 induced *Pfkfb3* mRNA decay by binding to AREs in the 3′ UTR, curbing glycolysis and immune responses of AMs and aggravating SII and secondary pneumonia.

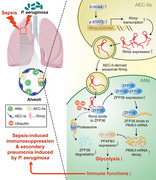

## 1 Introduction

Sepsis is a life‐threatening syndrome characterized by a dysregulated systemic inflammatory response to infection that can be triggered by various factors, such as infectious diseases, trauma, shock, and other acute or critical conditions.^[^ ^1^ ^]^ Globally, sepsis affects ≈49 million individuals annually, resulting in 11 million deaths and accounting for 19.7% of global mortality.^[^ ^2^ ^]^ The pathophysiology of sepsis is characterized by a profound imbalance between pro‐inflammatory and anti‐inflammatory responses. Excessive inflammation and its associated cytokine storms have been recognized as major contributors to sepsis‐related mortality. However, therapies that target inflammation have largely failed to improve patient outcomes.^[^ ^3^, ^4^ ^]^ By contrast, sepsis‐induced immunosuppression (SII) leads to immune dysfunction, rendering patients highly susceptible to secondary infections and contributing to high rehospitalization rates and mortality.^[^ ^5^, ^6^ ^]^ Secondary pneumonia, a frequent complication of SII, is one of the leading causes of death in patients with sepsis, with an incidence ranging from 30% to 50%.^[^ ^7^ ^]^ Despite their high clinical relevance, the mechanisms driving SII and secondary pneumonia remain poorly understood, and effective interventions are still lacking. Therefore, the identification of reliable biomarkers and therapeutic targets to mitigate SII is urgently needed to improve sepsis outcomes.

SII is characterized by the impaired function of immune effector cells and excessive activation of immunosuppressive pathways. Alveolar macrophages (AMs) are crucial innate immune cells in the lungs and are responsible for vital immune functions, including phagocytosis, antigen presentation, cytokine secretion, and efferocytosis.^[^ ^8^ ^]^ These cells play a critical role in maintaining pulmonary immune homeostasis and defending against pathogens.^[^ ^9^ ^]^ In sepsis, AM function is severely compromised, as evidenced by the reduced phagocytic capacity and increased production of anti‐inflammatory cytokines.^[^ ^10^ ^]^ Restoration of AM function alleviates secondary pneumonia in sepsis, highlighting the potential of AM as a therapeutic agent.^[^ ^11^, ^12^ ^]^ Following activation, macrophages undergo a metabolic shift from mitochondrial oxidative phosphorylation to aerobic glycolysis, a phenomenon known as the “Warburg effect.”^[^ ^13^ ^]^ This metabolic reprogramming is crucial for supporting the inflammatory response and pathogen clearance capacity of AMs.^[^ ^14^, ^15^ ^]^ However, glycolytic dysfunction in AMs following sepsis is well‐documented,^[^ ^16^, ^17^, ^18^ ^]^ and enhancing AM glycolysis has shown promise for restoring immune function, suggesting that targeting glycolysis may provide a therapeutic avenue for addressing SII and secondary pneumonia. However, the molecular mechanisms underlying the reprogramming of glucose metabolism in AMs during sepsis remain poorly understood.

Type II alveolar epithelial cells (AEC‐IIs) are essential components of the alveolar structure and are responsible for surfactant production, regulation of alveolar fluid volume, modulation of pulmonary innate immune responses, and promotion of epithelial repair after injury.^[^ ^19^, ^20^, ^21^ ^]^ AEC‐II dysfunction has been implicated in several pulmonary diseases, including chronic obstructive pulmonary disease,^[^ ^22^ ^]^ acute respiratory distress syndrome (ARDS),^[^ ^23^ ^]^ and idiopathic pulmonary fibrosis (IPF).^[^ ^24^ ^]^ Importantly, AEC‐IIs are also critical in the pathogenesis of post‐sepsis lung injury and can influence disease outcomes.^[^ ^23^, ^25^, ^26^ ^]^ However, the precise role of AEC‐IIs in the regulation of SII and secondary pneumonia remains unclear. Owing to their anatomical proximity, AEC‐IIs and AMs engage in extensive crosstalk, which is mediated primarily by exosomes —extracellular vesicles ranging from 30 to 150 nm in diameter. Under healthy conditions, AEC‐IIs regulate AM density in the alveoli via exosome‐mediated signaling of granulocyte‐macrophage colony‐stimulating factor,^[^ ^27^ ^]^ whereas AMs are responsible for clearing excess surfactants produced by AEC‐IIs.^[^ ^28^ ^]^ However, during pathological states, intercellular communication is disrupted, and exosomes play a critical role in modulating pulmonary inflammation. For example, AMs may suppress inflammation by transferring suppressors of cytokine signaling proteins to AEC‐IIs through exosomes,^[^ ^29^ ^]^ whereas AEC‐II‐derived exosomes can exacerbate asthma inflammation by promoting AM proliferation and chemotaxis.^[^ ^30^ ^]^ Furthermore, AEC‐II‐derived exosomes have been shown to influence metabolic reprogramming and immune responses of AMs in ARDS and IPF.^[^ ^31^ ^]^ However, the role of AEC‐II‐derived exosomes in regulating glycolysis and immune function in AMs during sepsis remains unclear.

The biological functions of exosomes are largely determined by their cargo, which includes various nucleic acids such as long noncoding RNAs (lncRNAs). lncRNAs, defined as noncoding RNAs longer than 200 nucleotides, are involved in regulating gene expression at both the transcriptional and post‐transcriptional levels through interactions with proteins and other RNAs.^[^ ^32^, ^33^ ^]^ LncRNAs are highly enriched in exosomes and play crucial roles in mediating intercellular communication, particularly in the context of sepsis, where they contribute to the regulation of organ injury in the lungs, heart, liver, brain, and kidneys.^[^ ^34^, ^35^, ^36^ ^]^ Exosomal lncRNAs are key regulators of sepsis progression, suggesting their potential use as biomarkers and therapeutic targets.^[^ ^37^ ^]^ Although several studies have examined the involvement of lncRNAs in SII,^[^ ^38^, ^39^ ^]^ the role of exosomal lncRNAs in the regulation of SII remains underexplored.

In this study, we demonstrate that AEC‐II‐derived exosomes drive glycolytic defects and immune dysfunction in AMs following sepsis, thereby exacerbating SII and secondary pneumonia. We identified an lncRNA, the RNA component of the mitochondrial RNA processing endoribonuclease (Rmrp), as a key exosomal cargo responsible for regulating glycolysis in AMs. Further investigation revealed that Rmrp binds to and stabilizes zinc finger protein 36 (ZFP36), facilitating the degradation of 6‐phosphofructo‐2‐kinase/fructose‐2,6‐bisphosphatase 3 (PFKFB3) mRNA and inhibiting AM glycolysis. Targeted inhibition of Rmrp in AMs significantly alleviated SII and secondary pneumonia in a murine model. Notably, we also observed a positive correlation between exosomal Rmrp levels and immune suppression in AMs from patients with sepsis, suggesting its potential as a prognostic marker for poor outcomes in sepsis. These findings offer new insights into the role of AEC‐II‐derived exosomal Rmrp in the pathogenesis of SII and secondary pneumonia and highlight Rmrp as a promising therapeutic target for improving sepsis outcomes.

## 2 Results

### 2.1 AEC‐IIs Inhibit Immune Responses and Glycolysis in AMs via Exosomal Transmission Following Sepsis

To investigate the cellular mechanisms underlying SII, we isolated AMs, neutrophils, and natural killer (NK) cells at various time points after cecal ligation and puncture (CLP), a widely used sepsis model, and assessed their immune responses using an in vitro immune tolerance model (Figure S1A, Supporting Information). Upon lipopolysaccharide (LPS) stimulation, AMs from CLP mice exhibited significantly reduced production of pro‐inflammatory cytokines and lactate compared to the sham control group (Figures S1B and S2A, Supporting Information), suggesting impaired immune function. By contrast, neutrophils and NK cells demonstrated enhanced immune activation following sepsis, as evidenced by increased cytokine production upon LPS stimulation (Figure S3A–D, Supporting Information). These results indicate that AMs, rather than neutrophils or NK cells, are primarily responsible for the immunosuppressive phenotype observed in sepsis. Based on these observations, we selected 48 h post‐CLP as the time point for subsequent experiments when AM immune responses and glycolysis were most significantly compromised.

Given the anatomical proximity and functional interactions between AECs and AMs, we hypothesized that AEC‐IIs modulate AM function in the alveolar microenvironment. To test this hypothesis, we isolated AEC‐Is (type I) and AEC‐IIs from CLP mice 48 h post‐surgery and cocultured them with primary AMs in a Transwell system (Figures S1C and S2B, Supporting Information). After LPS stimulation, AMs exhibited robust immune activation, as indicated by increased tumor necrosis factor‐α (TNF‐α), interleukin (IL)‐6, IL‐1β, and lactate production (Figure S1D, Supporting Information), along with upregulated CD86 and major histocompatibility complex‐II (MHC‐II) expression (Figure S1E, F, Supporting Information). In addition, AMs exhibited an enhanced extracellular acidification rate (ECAR), glucose uptake, and cellular glucose‐6‐phosphate (G6P) and pyruvate levels after LPS stimulation (Figure S1G–J, Supporting Information), suggesting potentiated glycolytic activity. By contrast, coculture with AEC‐IIs, but not with AEC‐Is, significantly impaired the immune responses and glycolytic activity in AMs (Figures S1D–J and S4A–G, Supporting Information). To further corroborate these findings, we used the mouse AEC line MLE‐12, which similarly inhibited AM immune responses and glycolysis when cocultured with AMs after LPS stimulation (Figure S5A–H, Supporting Information). Collectively, these data suggest that AEC‐IIs, rather than AEC‐Is or other immune cells, play a central role in the inhibition of AM function following sepsis.

**Figure 1:**
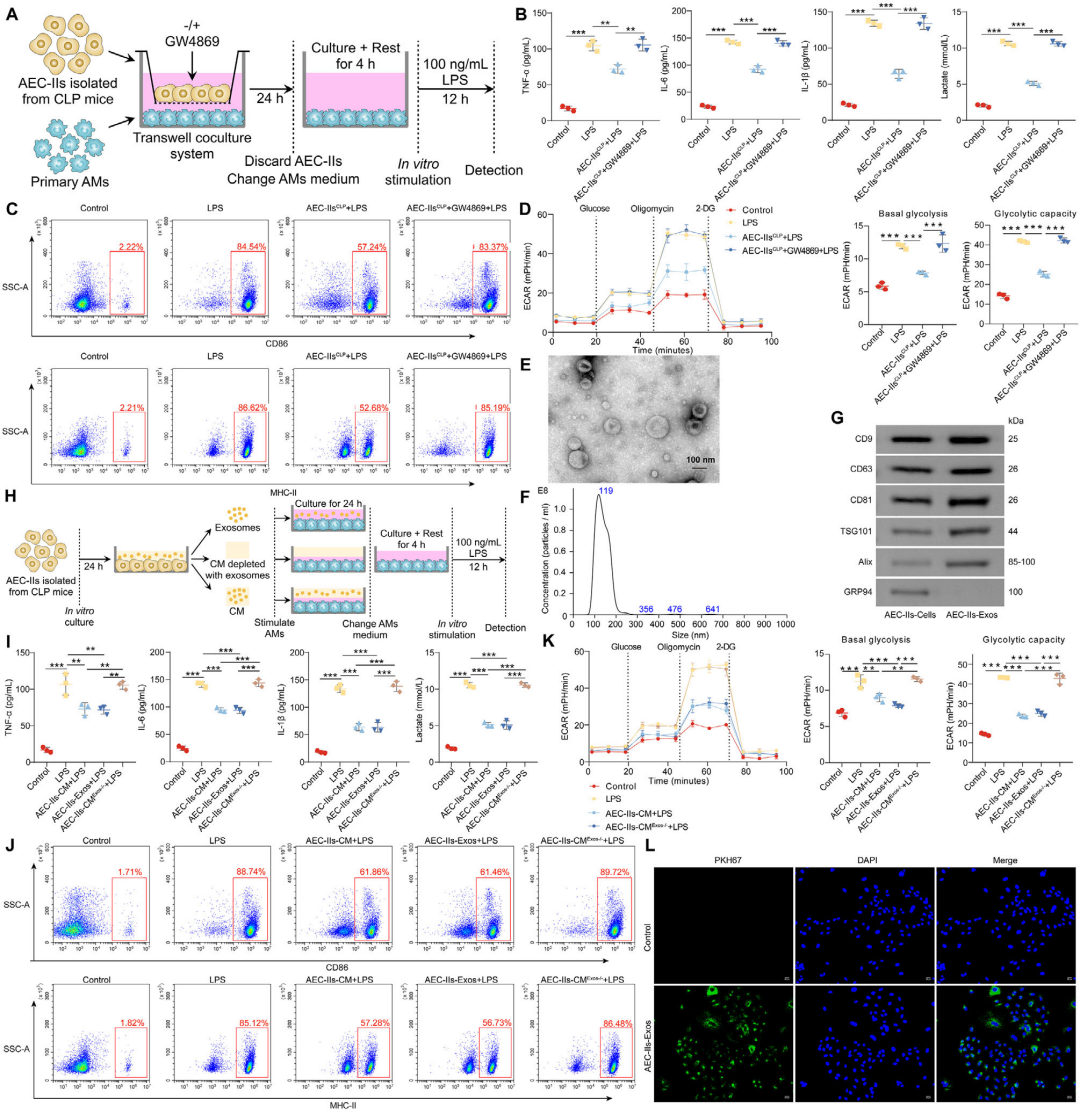
AEC‐IIs inhibit immune responses and glycolysis in AMs via exosome‐mediated communication after sepsis. A) Experimental scheme for panels 1B–D. B) Enzyme‐linked immunosorbent assay (ELISA) of TNF‐α, IL‐6, IL‐1β, and lactate levels in the supernatant of AMs cocultured with AEC‐IIs and subsequently stimulated with LPS (n = 3/group). C) Flow cytometric analysis of CD86 and MHC‐II expression on AMs. Representative flow cytometry plots are shown. D) Seahorse extracellular flux analysis was performed to determine the ECAR of AMs cocultured with AEC‐IIs and subsequently stimulated with LPS, and the basal glycolysis and glycolytic capacity were calculated (n = 3/group). E) Representative TEM image showing exosomes secreted by AEC‐IIs. Scale bar: 100 nm. F) Nanoparticle tracking analysis (NTA) of the size distribution and particle count of AEC‐II‐derived exosomes. G) Western blot (WB) analysis of exosomal markers (CD9, CD63, CD81, TSG101, and Alix) and the cellular marker GRP94 in AEC‐IIs or exosomes isolated from AEC‐IIs. H) Schematic overview of the experimental design for panels 1I–K. I) ELISA of TNF‐α, IL‐6, IL‐1β, and lactate in the supernatant of AMs treated with AEC‐II‐CM or AEC‐II‐derived exosomes (n = 3/group). J) Flow cytometry analysis of CD86 and MHC‐II levels in AMs following treatment with AEC‐II‐CM or AEC‐II‐derived exosomes. Representative flow cytometry plots are shown. K) Seahorse extracellular flux analysis was performed to determine the ECAR of AMs treated with AEC‐II‐CM or AEC‐II‐derived exosomes, and the basal glycolysis and glycolytic capacity were calculated (n = 3/group). L) Representative fluorescence microscopy images showing AMs incubated with PKH67‐labeled exosomes derived from AEC‐IIs. Scale bar: 25 µm. Data are presented as mean ± standard deviation (SD). Statistical significance was determined using one‐way ANOVA followed by Tukey's post hoc test. ^**^ *p* < 0.01, ^***^ *p* < 0.001.

To investigate the underlying mechanism, we hypothesized that AEC‐IIs regulate AM function through exosomes, which are small extracellular vesicles that mediate intercellular communication. In the Transwell coculture system, we treated AEC‐IIs with the exosome secretion inhibitor GW4869 to block exosome release (**Figure** **1**A). Notably, inhibition of exosome secretion from AEC‐IIs completely abolished the impairment of AM immune responses (Figure 1B,C; Figure S6A, Supporting Information) and glycolysis (Figures 1D; Figure S6B–D, Supporting Information) induced by coculture with AEC‐IIs. Similar results were obtained when MLE‐12 cells were used instead of AEC‐IIs (Figure S6E–L, Supporting Information), further supporting the role of exosomes in mediating these effects.

Next, we isolated exosomes from the conditioned medium (CM) of AEC‐IIs by ultracentrifugation. Transmission electron microscopy (TEM) revealed that the AEC‐II‐derived exosomes exhibited a characteristic cup‐shaped morphology (Figure 1E) and a size range of 30–150 nm (Figure 1F). Markers such as CD9, CD63, CD81, tumor susceptibility gene 101 (TSG101), and ALG‐2‐interacting protein‐X (Alix) were detected in AEC‐II‐derived exosomes, whereas the endoplasmic reticulum marker glucose‐regulated protein 94 (GRP94) was absent, confirming the successful isolation of exosomes (Figure 1G). We treated AMs with AEC‐II‐conditioned medium (AEC‐II‐CM), AEC‐II‐derived exosomes, or exosome‐depleted AEC‐II‐CM (Figure 1H). Both AEC‐II‐CM and AEC‐II‐derived exosomes effectively inhibited the AM immune responses and glycolysis following LPS stimulation. However, the inhibitory effects of AEC‐II‐CM on AM function were reversed upon the depletion of exosomes (Figure 1I–K; Figure S7A–D, Supporting Information), further supporting the exosomal mediation of this process. Similarly, exosomes derived from MLE‐12 cells also suppressed the immune and glycolytic responses in AMs (Figure S7E–N, Supporting Information).

To confirm the uptake of AEC‐II‐derived exosomes by AMs, we labeled exosomes from both AEC‐IIs and MLE‐12 cells with PKH67 dye and incubated them with AMs for 24 h (Figure S8A, B, Supporting Information). Confocal microscopy revealed that AMs efficiently internalized PKH67‐labeled exosomes (Figures 1L; Figure S8C, Supporting Information), confirming that exosomal transmission occurred directly from AEC‐IIs to AMs.

Collectively, these results provide compelling evidence that AEC‐IIs inhibit immune responses and glycolysis in AMs by secreting exosomes after sepsis, highlighting the critical role of exosomal communication in the pathogenesis of SII.

### 2.2 AEC‐II‐Derived Exosomal Rmrp Inhibits Immune Responses and Glycolysis in AMs After Sepsis

**Figure 2:**
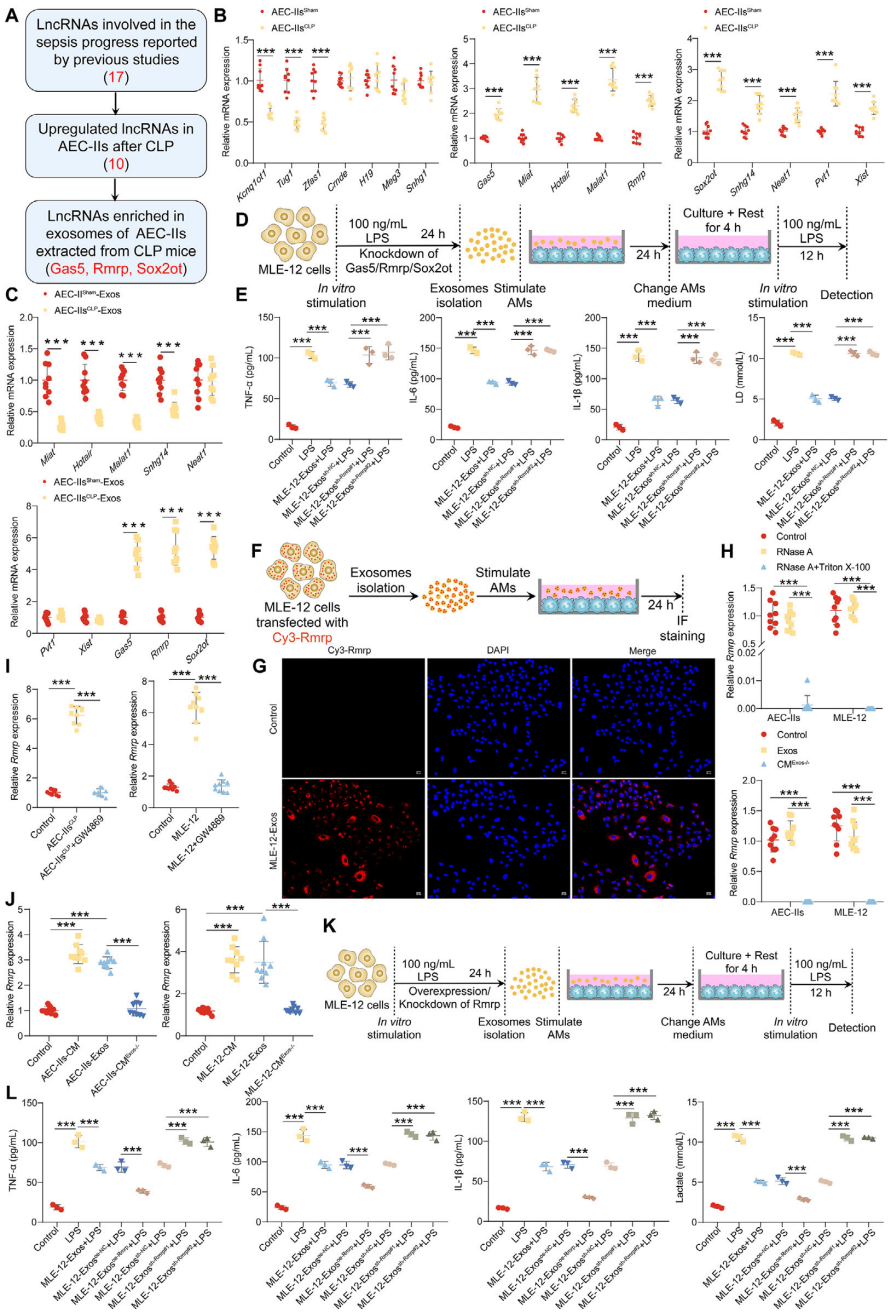
AEC‐II‐derived exosomal Rmrp inhibits immune responses and glycolysis in AMs after sepsis. A) Flowchart outlining the screening of candidate lncRNAs in AEC‐II‐derived exosomes that regulate glycolysis and immune responses in AMs. B) Reverse‐transcription quantitative PCR (RT‐qPCR) analysis of lncRNA expression in AEC‐IIs derived from sham or CLP mice (n = 9/group). C) RT‐qPCR analysis of Miat, Hotair, Malat1, Snhg14, Neat1, Pvt1, Xist, Gas5, Rmrp, and Sox2ot expression in exosomes of AEC‐IIs isolated from sham mice or CLP mice (n = 9/group). D) Schematic representation of the experimental setup for panels 2E and S9B, C. E) ELISA of TNF‐α, IL‐6, IL‐1β, and lactate levels in the supernatant of AMs treated with MLE‐12 cell‐derived exosomes and subsequently stimulated with LPS (n = 3/group). F) Experimental scheme for panel 2G. G) Representative fluorescence images of AMs incubated with Cy3‐labeled exosomes from MLE‐12 cells. Scale bar: 25 µm. H) RT‐qPCR analysis of Rmrp in CM from AEC‐IIs or MLE‐12 cells treated with RNase A or Triton X‐100 (upper panel), and Rmrp expression in CM or exosomes from AEC‐IIs or MLE‐12 cells (lower panel) (n = 9/group). I) RT‐qPCR analysis of Rmrp in AMs cocultured with AEC‐IIs (left) or MLE‐12 cells (right) (n = 9/group). J) RT‐qPCR analysis of Rmrp in AMs treated with CM or exosomes derived from AEC‐IIs (left) or MLE‐12 cells (right) (n = 9/group). K) Schematic overview of the experimental design for panels 2L and S10B‐H. L) ELISA of TNF‐α, IL‐6, IL‐1β, and lactate levels in the supernatant of AMs after coculture with MLE‐12 cell‐derived exosomes and subsequent LPS stimulation (n = 3/group). Data are presented as mean ± SD. Statistical significance was determined using two‐way ANOVA followed by Sidak's post hoc test (B, C, and H) or one‐way ANOVA followed by Tukey's post hoc test (E, I, J, and L). ^***^ *p* < 0.001.

LncRNAs are key cargos within exosomes and play pivotal roles in sepsis progression.^[^ ^32^, ^34^, ^35^ ^]^ We hypothesized that AEC‐II‐derived exosomes mediate immune tolerance and impair glycolysis in AMs by delivering specific lncRNAs. To test this, AEC‐IIs were isolated from mice 48 h after CLP or sham surgery, and exosomes were subsequently isolated from the CM of the AEC‐IIs (**Figure** **2**A). We evaluated the expression levels of several lncRNAs implicated in sepsis progression, including growth arrest‐specific 5 (Gas5), the RNA component of mitochondrial Rmrp, and SOX2 overlapping transcript (Sox2ot), in AEC‐IIs and their derived exosomes. Consistent with previous studies,^[^ ^34^, ^35^ ^]^ we found that Gas5, Rmrp, and Sox2ot, but not other lncRNAs, were significantly upregulated in both the cellular (Figure 2B) and exosomal fractions (Figure 2C) of AEC‐IIs after CLP. We hypothesized that these three lncRNAs, but not others, are likely the cargo of AEC‐II‐derived exosomes that induce impaired immune tolerance and glycolysis in AMs.

To determine which of these lncRNAs is responsible for modulating AM function, we knocked down Gas5, Rmrp, or Sox2ot in MLE‐12 cells (Figure S9A, Supporting Information) and isolated exosomes to treat AMs (Figure 2D). Knockdown of Rmrp (Figure 2E), but not of Gas5 (Figure S9B, Supporting Information) or Sox2ot (Figure S9C, Supporting Information), abolished the inhibitory effects of MLE‐12 cell‐derived exosomes on pro‐inflammatory cytokine secretion and lactate production in AMs. These results suggest that AEC‐II‐derived exosomes primarily mediate immune tolerance and glycolytic impairment in AMs via Rmrp.

Next, we examined the dynamic changes in Rmrp levels in bronchoalveolar lavage fluid (BALF) exosomes, AEC‐IIs, and AMs after sepsis. Rmrp levels were significantly elevated in BALF exosomes, AEC‐IIs, and AMs from CLP mice compared to those in the sham group, peaking at 48 h post‐CLP (Figure S10A, Supporting Information). To confirm exosomal transfer, MLE‐12 cells were transfected with Cy3‐tagged Rmrp, and their exosomes were collected to stimulate AMs (Figure 2F). Confocal microscopy revealed Cy3 fluorescence in AMs (Figure 2G), confirming that Rmrp was successfully transferred from AEC‐II to AMs via exosomes.

To further investigate the mechanism of Rmrp transfer, we treated the CM from AEC‐IIs or MLE‐12 cells with RNase A or Triton X‐100. RNase A treatment did not affect Rmrp levels in the CM, whereas cotreatment with RNase A and Triton X‐100 markedly reduced Rmrp levels (Figure 2H). Notably, Rmrp levels in exosomes and CM were nearly equivalent, but depletion of exosomes from CM significantly decreased Rmrp expression (Figure 2H). These findings suggest that Rmrp is encapsulated in exosomes rather than released directly into the extracellular space.

In the Transwell coculture system of AEC‐IIs/MLE‐12 cells with AMs, we observed that AEC‐IIs/MLE‐12 cells significantly increased Rmrp levels in AMs in the lower chamber. However, inhibition of exosome secretion from AEC‐IIs/MLE‐12 cells using GW4869 prevented the elevation of Rmrp in AMs (Figure 2I). Additionally, exosomes derived from AEC‐IIs or MLE‐12 cells, but not from exosome‐depleted CM, increased Rmrp levels in AMs (Figure 2J), further confirming that Rmrp was delivered to AMs via exosomes.

To explore the functional consequences of Rmrp transfer, we overexpressed or knocked down Rmrp in MLE‐12 cells, followed by LPS stimulation and exosome isolation after AM treatment (Figure 2K). Upon treatment with MLE‐12 cell‐derived exosomes, Rmrp levels in AMs increased significantly (Figure S10B, Supporting Information), leading to impaired pro‐inflammatory cytokine production (Figure 2L), lactate secretion (Figure 2L), CD86 and MHC‐II expression (Figure S10C, D, Supporting Information), and glycolysis (Figure S10E–H, Supporting Information). Overexpression of Rmrp further augmented the inhibitory effects of MLE‐12 cell‐derived exosomes, whereas Rmrp knockdown abrogated the immune tolerance and glycolytic defects induced by MLE‐12‐derived exosomes (Figure 2L; Figure S10C–H, Supporting Information). These findings demonstrate that AEC‐II‐derived exosomal Rmrp plays a crucial role in suppressing immune responses and glycolysis in AMs following sepsis.

### 2.3 Rmrp Inhibits Immune Responses and Glycolysis in AMs by Downregulating PFKFB3

**Figure 3:**
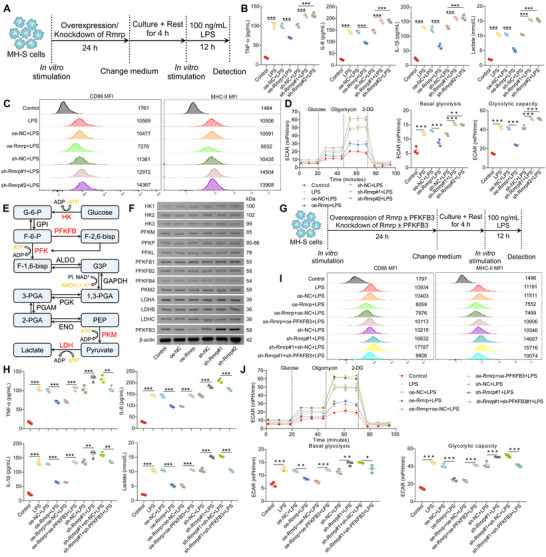
Rmrp restrains immune responses and glycolysis in AMs by decreasing PFKFB3. A) Schematic overview of the experimental design for panels 3B–D and S11B‐E. B) ELISA of TNF‐α, IL‐6, IL‐1β, and lactate concentrations in the supernatant of MH‐S cells after Rmrp overexpression or knockdown, followed by LPS treatment (n = 3/group). C) Flow cytometry analysis of CD86 and MHC‐II levels in MH‐S cells. Representative flow cytometry plots are shown. D) Seahorse extracellular flux analysis was performed to determine the ECAR of MH‐S cells after Rmrp overexpression or knockdown, followed by LPS treatment, and the basal glycolysis and glycolytic capacity were calculated (n = 3/group). E) Diagram of the glycolysis pathway, highlighting the rate‐limiting enzymes in red. F) WB analysis of glycolytic rate‐limiting enzymes in MH‐S cells after Rmrp overexpression or knockdown. G) Schematic overview of the experimental design for panels 3H–J and S12K‐N. H) ELISA of TNF‐α, IL‐6, IL‐1β, and lactate concentrations in the supernatant of MH‐S cells after Rmrp/PFKFB3 overexpression or silencing, followed by LPS stimulation (n = 3/group). I) Flow cytometry analysis of CD86 and MHC‐II expression in MH‐S cells. Representative flow cytometry plots are shown. J) Seahorse extracellular flux analysis was performed to determine the ECAR of MH‐S cells after Rmrp/PFKFB3 overexpression or silencing, followed by LPS stimulation, and the basal glycolysis and glycolytic capacity were calculated (n = 3/group). Data are presented as mean ± SD. Statistical significance was determined using one‐way ANOVA followed by Tukey's post hoc test. ^*^ *p* < 0.05, ^**^ *p* < 0.01, ^***^ *p* < 0.001.

Our previous data demonstrated that AEC‐IIs transmit Rmrp to AMs via exosomes, thereby suppressing immune responses and glycolysis in AMs. To further investigate the role of Rmrp in regulating immune responses and glycolysis in AMs, we overexpressed or silenced Rmrp in the mouse AM cell line MH‐S cultured in vitro (Figure S11A, Supporting Information). Following a 24 h incubation, the medium was replaced, and the cells were rested for 4 h before being stimulated with LPS for 12 h (**Figure** **3**A). LPS treatment significantly increased the secretion of pro‐inflammatory cytokines (Figure 3B), lactate production (Figure 3B), expression of CD86 and MHC‐II (Figure 3C; Figure S11B, Supporting Information), and glycolysis (Figure 3D; Figure S11C–E, Supporting Information) in MH‐S cells. Notably, Rmrp knockdown further intensified these effects, whereas Rmrp overexpression reversed them (Figure 3B–D; Figure S11B–E, Supporting Information). These results confirmed that Rmrp effectively modulates immune responses and glycolysis in AMs.

Glycolysis plays a crucial role in sustaining the immune function of AMs, and its rate and direction are tightly regulated by glycolytic rate‐limiting enzymes (Figure 3E).^[^ ^40^ ^]^ To explore the effect of Rmrp on glycolysis, we assessed the expression of key glycolytic enzymes in MH‐S cells. Among all the glycolytic enzymes tested, only PFKFB3 exhibited a marked increase upon Rmrp silencing and a notable decrease following Rmrp overexpression (Figures 3F; Figure S11F–G, Supporting Information). This suggests that Rmrp may regulate both immune function and glycolysis in AMs by modulating PFKFB3 expression. PFKFB3 catalyzes the production of fructose‐2,6‐bisphosphate, which activates phosphofructokinase‐1, thereby promoting glycolysis.^[^ ^41^ ^]^ PFKFB3 induces a pro‐inflammatory phenotype in macrophages during sepsis;^[^ ^41^ ^]^ however, its role in immune tolerance in AMs after SII remains unclear.

To investigate the role of PFKFB3 in the regulation of AM immune responses and glycolysis, we manipulated PFKFB3 expression in MH‐S cells before LPS stimulation (Figure S12A–C, Supporting Information). The overexpression of PFKFB3 led to enhanced immune responses (Figure S12D–F, Supporting Information), increased lactate production (Figure S12D, Supporting Information), and upregulated glycolysis (Figure S12G–J, Supporting Information) in MH‐S cells. Conversely, silencing PFKFB3 significantly attenuated the LPS‐induced immune activation (Figure S12D–F, Supporting Information) and glycolytic activity (Figure S12G–J, Supporting Information). These findings indicate that PFKFB3 is essential for proper immune and metabolic responses of AMs.

Furthermore, overexpression of PFKFB3 in MH‐S cells abolished the immune tolerance and glycolytic impairment induced by Rmrp (Figure 3G–J; Figure S12K–N, Supporting Information), whereas silencing PFKFB3 in Rmrp‐downregulated MH‐S cells reversed the enhanced immune responses and glycolysis (Figure 3G–J; Figure S12K‐N). These results provide compelling evidence that Rmrp impairs immune responses and glycolysis in AMs by downregulating PFKFB3 expression.

### 2.4 Rmrp Protects ZFP36 from Ubiquitin–Proteasome‐Dependent Degradation

**Figure 4:**
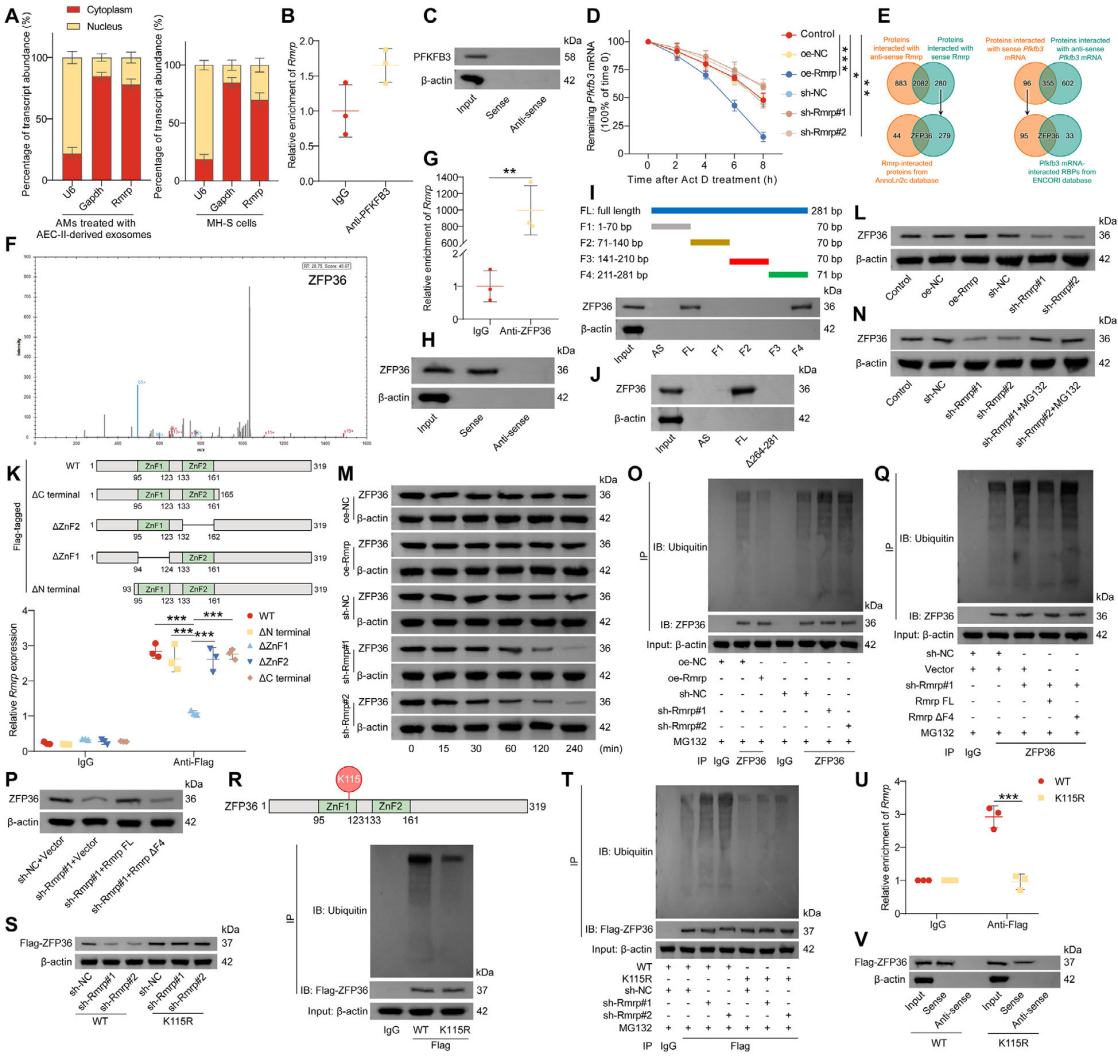
Rmrp protects ZFP36 from UPS‐dependent degradation. A) RT‐qPCR analysis of Rmrp expression in the cytoplasm and nucleus of AMs or MH‐S cells. GAPDH and U6 were used as positive controls for the cytoplasm and nucleus, respectively. B) RIP analysis of the binding between PFKFB3 and Rmrp in MH‐S cells. C) RNA pull‐down assay to verify the interaction between PFKFB3 and Rmrp in MH‐S cells. D) RT‐qPCR analysis of *Pfkfb3* mRNA in Rmrp‐overexpressed or ‐silenced MH‐S cells after treatment with actinomycin D (5 µg mL^−1^) (n = 3/group). E) Biotin–RNA pull‐down assay using FL Rmrp or *Pfkfb3* mRNA transcript (sense) and the anti‐sense Rmrp or *Pfkfb3* mRNA, followed by MS. Rmrp‐interacted proteins or *Pfkfb3* mRNA‐interacted proteins were intersected with Rmrp‐interacted proteins predicted using AnnoLnc2 database (left) or *Pfkfb3* mRNA‐interacted proteins from ENCORI database (right), respectively. F) MS profile of ZFP36 protein. G) RIP analysis of the interaction between ZFP36 with Rmrp in MH‐S cells (n = 3/group). H) RNA pull‐down analysis of the binding between ZFP36 with Rmrp in MH‐S cells. I) Schematic diagram of FL and truncated Rmrp fragments (upper). RNA pull‐down assay to identify the binding between ZFP36 and Rmrp fragments in MH‐S cells (lower). J) RNA pull‐down assay was used to determine the binding between ZFP36 with anti‐sense Rmrp, FL Rmrp, or 264–281 truncated Rmrp fragments in MH‐S cells. K) Schematic illustration of FL and truncated flag‐tagged ZFP36 (upper). RIP analysis of the binding between truncated ZFP36 and Rmrp in MH‐S cells (lower) (n = 3/group). L) WB analysis of ZFP36 protein levels in MH‐S cells after Rmrp overexpression or knockdown. M) MH‐S cells overexpressing or silencing Rmrp were treated with cycloheximide (100 µg mL^−1^) for different time points, and ZFP36 expression was assessed using WB. N) WB analysis of ZFP36 in MH‐S cells after Rmrp knockdown or MG132 treatment. O) Polyubiquitination analysis of ZFP36 levels in MH‐S cells after Rmrp overexpression or knockdown. P) WB analysis and Q) polyubiquitination analysis of ZFP36 expression and ubiquitination, respectively, in MH‐S cells where Rmrp was silenced and then transfected with FL Rmrp or Rmrp ΔF4 vector. R) Potential ubiquitination site of ZFP36 predicted using the PhosphoSitePlus database (upper). Polyubiquitination analysis of Flag‐ZFP36 WT or K115R MUT (lower). S) WB analysis and T) polyubiquitination analysis of Flag‐ZFP36 expression and ubiquitination in MH‐S cells transfected with WT or K115R Flag‐ZFP36, followed by Rmrp silencing. U) RIP and V) RNA pull‐down assays were used to assess the interaction between ZFP36 and Rmrp in MH‐S cells (n = 3/group). Data are presented as mean ± SD. Statistical significance was determined using one‐way ANOVA followed by Tukey's post hoc test (D), Student's *t*‐test (B, G), or two‐way ANOVA followed by Sidak's post hoc test (K, U). ^*^ *p* < 0.05, ^**^ *p* < 0.01, ^***^ *p* < 0.001.

To investigate the mechanism by which Rmrp regulates PFKFB3 expression, we first examined its cellular localization, because the molecular functions of lncRNAs are often closely linked to their subcellular distribution.^[^ ^42^ ^]^ In AMs treated with AEC‐II‐derived exosomes and the untreated MH‐S cells, Rmrp was predominantly localized in the cytoplasm (**Figure** **4**A). As cytoplasmic lncRNAs typically exert their functions at the post‐transcriptional level by interacting with proteins,^[^ ^42^ ^]^ we examined whether Rmrp directly binds to PFKFB3 using RNA immunoprecipitation (RIP) and RNA pull‐down assays. However, the anti‐PFKFB3 antibody failed to immunoprecipitate Rmrp (Figure 4B), and biotinylated Rmrp did not pull down PFKFB3 (Figure 4C), suggesting that Rmrp does not regulate PFKFB3 through a direct interaction.

Interestingly, we observed that Rmrp promoted the degradation of *Pfkfb3* mRNA in MH‐S cells (Figure 4D), leading us to hypothesize that Rmrp regulates *Pfkfb3* mRNA stability via an RNA‐binding protein (RBP). To identify potential RBPs involved in this process, we conducted a biotin–RNA pull‐down assay using full‐length (FL) Rmrp or *Pfkfb3* mRNA transcripts (sense) and anti‐sense Rmrp or *Pfkfb3* mRNA, followed by mass spectrometry (MS) to screen for proteins that interact with Rmrp and *Pfkfb3* mRNA. Furthermore, RNA pull‐down and MS identified Rmrp‐interacting proteins or *Pfkfb3* mRNA‐interacting proteins were intersected with Rmrp‐interacting proteins predicted using the AnnoLnc2 database and *Pfkfb3* mRNA‐interacting proteins from the ENCORI database, respectively. The ZFP36 protein was identified using intersection analysis (Figure 4E,F). RIP and RNA pull‐down assays confirmed that ZFP36 was bound to Rmrp (Figure 4G,H). To further map the region of Rmrp responsible for binding to ZFP36, we generated biotinylated FL Rmrp and several truncated mutants (MUTs) (F1: 1–70 bp, F2: 71–140 bp, F3: 141–210 bp, and F4: 211–281 bp) (Figure 4I). RNA pull‐down assays revealed that the F4 fragment of Rmrp was responsible for binding to ZFP36 (Figure 4I), and ZFP36 bound to biotinylated FL Rmrp but not to Rmrp truncated at nucleotides 264–281 (Figure 4J), which corresponded to the predicted binding sites identified using the AnnoLnc2 database (Figure S13A, Supporting Information). Simultaneously, FLAG‐tagged FL and truncated ZFP36 constructs were generated and transfected into MH‐S cells (Figure 4K). RIP assays showed that the zinc finger domain 1 (ZnF1) of ZFP36 specifically interacts with Rmrp (Figure 4K), confirming that Rmrp binds to the ZnF1 domain of ZFP36 through its F4 fragment.

Next, we assessed the effects of Rmrp on ZFP36 expression. Although Rmrp did not alter the *Zfp36* mRNA levels in MH‐S cells (Figure S13B, Supporting Information), it significantly increased ZFP36 protein expression (Figures 4L; Figure S13C, Supporting Information). Moreover, Rmrp knockdown promoted ZFP36 degradation, whereas Rmrp overexpression prolonged its half‐life (Figure 4M). The ubiquitin–proteasome system (UPS) is a major pathway that regulates protein stability in eukaryotic cells,^[^ ^43^ ^]^ and previous studies have shown that ZFP36 degradation is regulated by the UPS.^[^ ^44^, ^45^ ^]^ Proteasome inhibition by MG132 restored the ZFP36 levels, which were reduced by Rmrp knockdown (Figure 4N; Figure S13D, Supporting Information). Additionally, Rmrp overexpression inhibited, whereas Rmrp knockdown promoted, the ubiquitination of ZFP36 (Figure 4O), suggesting that Rmrp protects ZFP36 from UPS‐mediated degradation.

We investigated the role of the F4 fragment of Rmrp in regulating ZFP36 stability. Overexpression of FL Rmrp, but not a MUT version lacking the F4 fragment (Rmrp ΔF4), prevented the degradation (Figures 4P; Figure S13E, Supporting Information) and ubiquitination (Figure 4Q) of ZFP36 caused by Rmrp knockdown, highlighting the essential role of the F4 fragment in maintaining ZFP36 stability. Using the PhosphoSitePlus database, we identified lysine 115 (K115) in the ZnF1 domain of ZFP36 as a potential ubiquitination site (Figure 4R). Because the ZnF1 domain interacts with Rmrp, we hypothesized that Rmrp might inhibit K115 ubiquitination upon binding to ZFP36. K115 mutation significantly reduced ZFP36 ubiquitination (Figure 4R), confirming that K115 is a key ubiquitination site. Rmrp knockdown promoted the degradation (Figures 4S; Figure S13F, Supporting Information) and ubiquitination (Figure 4T) of wild‐type (WT) ZFP36 but had no effect on the expression (Figure 4S; Figure S13F, Supporting Information) or ubiquitination (Figure 4T) of the K115 MUT form of ZFP36. Additionally, RIP (Figure 4U) and RNA pull‐down assays (Figure 4V) revealed that the K115 mutation weakened interactions between ZFP36 and Rmrp. Collectively, these data suggest that Rmrp inhibits the ubiquitination of ZFP36 by masking the K115 site, thereby protecting ZFP36 from UPS‐mediated degradation.

### 2.5 ZFP36 Promotes *Pfkfb3* mRNA Decay Via Interaction with AU‐Rich Elements

**Figure 5:**
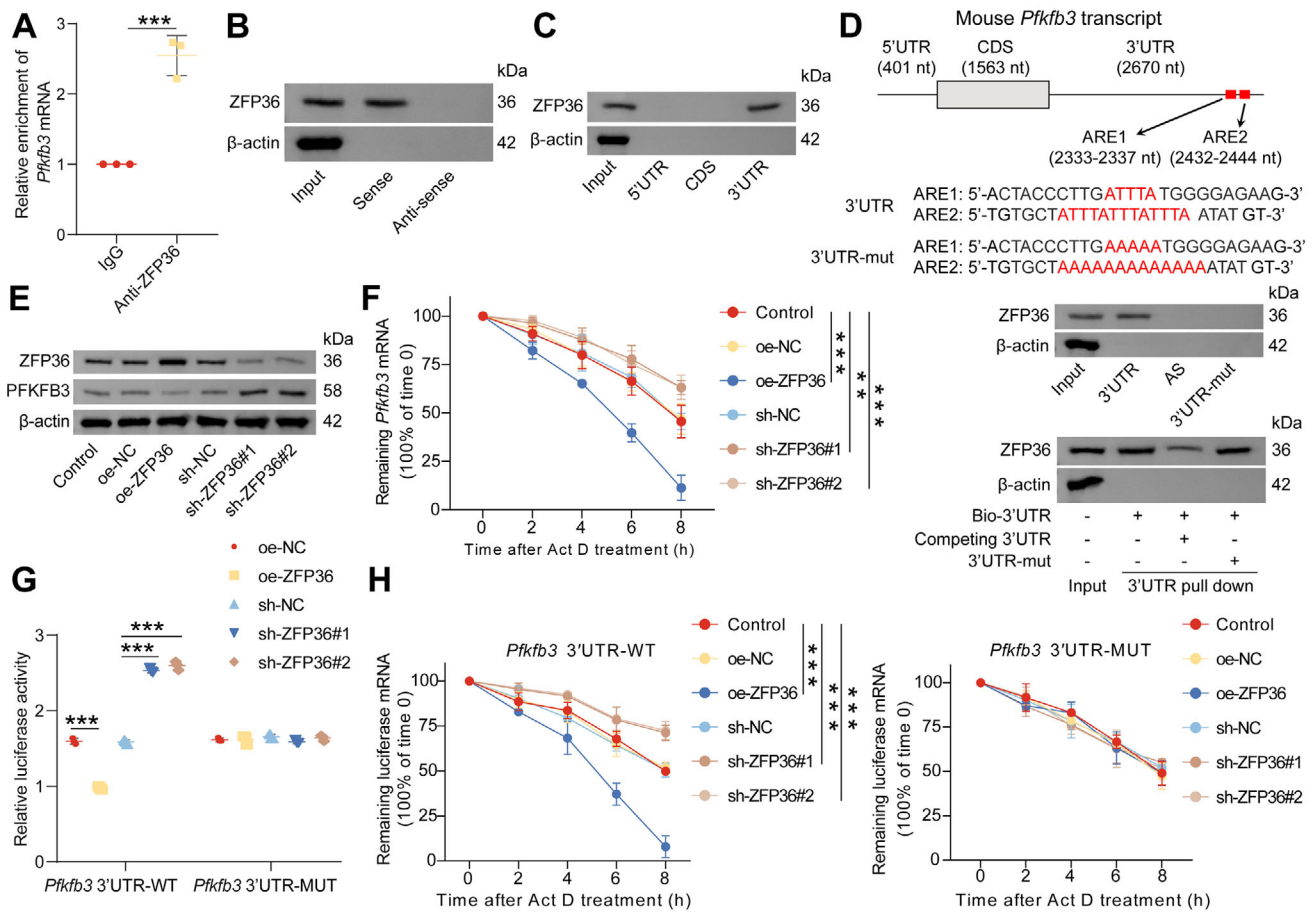
ZFP36 promotes *Pfkfb3* mRNA decay. A) RIP analysis of the binding between ZFP36 and *Pfkfb3* mRNA in MH‐S cells (n = 3/group). B) RNA pull‐down assay to verify the interaction between ZFP36 and *Pfkfb3* mRNA in MH‐S cells. C) RNA pull‐down assay to assess the binding of ZFP36 with different regions of *Pfkfb3* mRNA: 5′ UTR, CDS, or 3′ UTR. D) Schematic of the two AREs in the 3′ UTR of mouse *Pfkfb3* mRNA (upper panel). RNA pull‐down assay to analyze the interaction between ZFP36 and the *Pfkfb3* 3′ UTR or AREs‐MUT *Pfkfb3* 3′ UTR in MH‐S cells (middle). Streptavidin pull‐down of biotin‐labeled *Pfkfb3* 3′ UTR. The MH‐S cell lysates were treated with non‐labeled WT or AREs‐MUT *Pfkfb3* 3′ UTR (lower panel). E) WB analysis of ZFP36 and PFKFB3 protein levels in MH‐S cells after ZFP36 overexpression or knockdown. F) RT‐qPCR analysis of *Pfkfb3* mRNA in ZFP36‐overexpressed or ‐silenced MH‐S cells after treatment with Actinomycin D (5 µg mL^−1^) for different time points (n = 3/group). G) Luciferase activity measurement in MH‐S cells cotransfected with reporter constructs and ZFP36 expression vector or ZFP36‐specific short‐hairpin RNA (shRNA) (n = 3/group). H) Stability of luciferase mRNA in ZFP36‐overexpressed or ‐silenced MH‐S cells, measured using RT‐qPCR after Actinomycin D treatment (n = 3/group). Data are presented as mean ± SD. Statistical significance was assessed using Student's *t*‐test (A), one‐way ANOVA followed by Tukey's post hoc test (F, H), or two‐way ANOVA followed by Sidak's post hoc test (G). ^**^ *p* < 0.01, ^***^ *p* < 0.001.

Using RNA pull‐down and MS, we identified a potential binding between ZFP36 and *Pfkfb3* mRNA (Figure 4E). To explore this interaction, we first performed RIP assays, which demonstrated that the anti‐ZFP36 antibody successfully immunoprecipitated *Pfkfb3* mRNA (**Figure** **5**A). RNA pull‐down assays further confirmed that ZFP36 binds directly to biotinylated *Pfkfb3* mRNA (Figure 5B), suggesting a direct interaction. Notably, ZFP36 interacted with the 3′ untranslated region (3′ UTR) of *Pfkfb3* mRNA, but not with the 5′ UTR or coding sequence (CDS) (Figure 5C). These findings suggest that ZFP36 specifically targets the 3′ UTR of *Pfkfb3* mRNA for post‐transcriptional regulation.

Adenylate‐ and uridylate‐rich elements (AREs) within the 3′UTR of mRNAs are well‐established determinants of mRNA stability,^[^ ^46^ ^]^ and ZFP36 is known to mediate mRNA decay by binding to these AREs.^[^ ^47^ ^]^ We identified two AREs within the 3′ UTR of mouse *Pfkfb3* mRNA (Figure 5D) and found that mutating these AREs significantly impaired the interaction between ZFP36 and the *Pfkfb3* 3′ UTR (Figure 5D). Non‐biotin‐labeled WT 3′ UTR competitively inhibited the pull‐down of biotinylated *Pfkfb3* 3′ UTR by ZFP36, whereas the non‐biotin‐labeled MUT 3′ UTR, lacking AREs, did not exhibit this inhibitory effect (Figure 5D). These results confirm that ZFP36 interacts specifically with the *Pfkfb3* 3′ UTR via the AREs.

To assess the functional consequences of ZFP36 binding to *Pfkfb3* mRNA, we modulated ZFP36 expression in MH‐S cells. Overexpression of ZFP36 significantly reduced the half‐life of *Pfkfb3* mRNA (Figure 5F) and decreased PFKFB3 protein levels (Figure 5E; Figure S14A,B, Supporting Information), whereas ZFP36 knockdown prolonged the half‐life of *Pfkfb3* mRNA (Figure 5F) and increased PFKFB3 protein levels (Figure 5E; Figure S14A, B, Supporting Information). These findings suggest that ZFP36 negatively regulates *Pfkfb3* mRNA stability and PFKFB3 protein expression.

To further validate the role of ZFP36 in the regulation of *Pfkfb3* mRNA stability, we conducted luciferase reporter assays. ZFP36 overexpression significantly reduced the activity of luciferase reporters conjugated with the WT *Pfkfb3* 3′ UTR, but had no effect on luciferase reporters conjugated with AREs‐MUT 3′ UTR (Figure 5G). Similarly, ZFP36 knockdown markedly increased the activity of luciferase reporters with the WT *Pfkfb3* 3′ UTR but did not affect those with the AREs‐MUT 3′ UTR (Figure 5G). Additionally, ZFP36 overexpression shortened the half‐life of luciferase mRNA conjugated with the WT *Pfkfb3* 3′ UTR, whereas ZFP36 knockdown prolonged its half‐life. However, ZFP36 had no effect on the stability of luciferase mRNA conjugated with the AREs‐MUT 3′ UTR (Figure 5H).

Taken together, these results demonstrate that ZFP36 promotes *Pfkfb3* mRNA decay by specifically interacting with AREs within its 3′ UTR, thereby downregulating PFKFB3 expression and modulating glycolytic function in AMs.

### 2.6 Rmrp/ZFP36/PFKFB3 Axis Inhibits Immune Responses and Glycolysis in AMs

**Figure 6:**
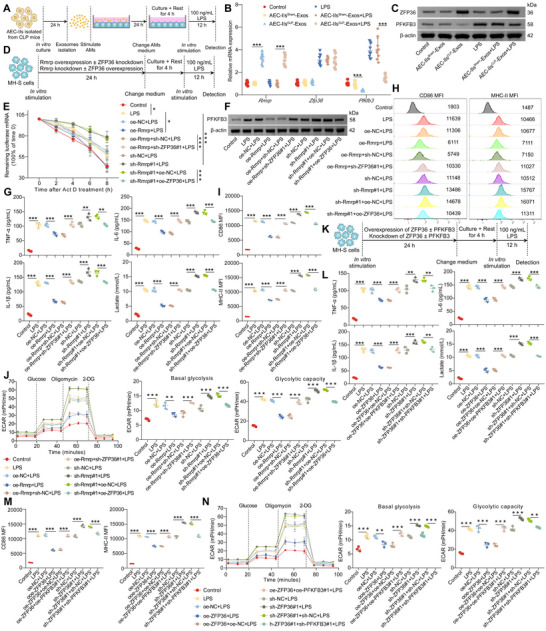
Rmrp/ZFP36/PFKFB3 axis inhibits immune responses and glycolysis in AMs. A) Experimental schematic for panels 6B, C, S15A. B) RT‐qPCR analysis of Rmrp, ZFP36, and PFKFB3 expression in AMs after treatment with AEC‐II‐derived exosomes (n = 9/group). C) WB analysis of ZFP36 and PFKFB3 protein levels in AMs treated with AEC‐II‐derived exosomes. D) Schematic overview of the experimental design for panels 6E–J, S15C–E. E) Stability of *Pfkfb3* mRNA in MH‐S cells assessed using RT‐qPCR after Actinomycin D (5 µg mL^−1^) treatment at different time points (n = 3/group). F) WB analysis of PFKFB3 protein levels in MH‐S cells following Rmrp/ZFP36 overexpression or knockdown. G) ELISA of TNF‐α, IL‐6, IL‐1β, and lactate concentrations in the supernatant of MH‐S cells after Rmrp/ZFP36 overexpression or silencing and subsequent LPS stimulation (n = 3/group). H) Flow cytometry analysis of CD86 and MHC‐II expression in MH‐S cells. Representative flow cytometry plots are shown. I) Mean fluorescence intensity (MFI) of CD86 (upper) and MHC‐II (lower) on MH‐S cells from panel H (n = 3/group). J) Seahorse extracellular flux analysis was performed to determine the ECAR of MH‐S cells after Rmrp/ZFP36 overexpression or silencing and subsequent LPS stimulation, and the basal glycolysis and glycolytic capacity were calculated (n = 3/group). K) Experimental schematic for panels 6L–N, S15F–I. L) ELISA of TNF‐α, IL‐6, IL‐1β, and lactate concentrations in the supernatant of MH‐S cells after ZFP36/PFKFB3 overexpression or knockdown and LPS stimulation (n = 3/group). M) MFI of CD86 (upper) and MHC‐II (lower) of MH‐S cells in panel S15F (n = 3/group). N) Seahorse extracellular flux analysis was performed to determine the ECAR of MH‐S cells after ZFP36/PFKFB3 overexpression or silencing and subsequent LPS stimulation, and the basal glycolysis and glycolytic capacity were calculated (n = 3/group). Data are presented as mean ± SD. Statistical significance was determined using two‐way ANOVA followed by Sidak's post hoc test (B) or one‐way ANOVA followed by Tukey's post hoc test (E, G, I, J, and L–N). ^*^ *p* < 0.05, ^**^ *p* < 0.01, ^***^ *p* < 0.001.

These data confirm that Rmrp upregulates ZFP36 expression, which, in turn, promotes the degradation of *Pfkfb3* mRNA. Next, we sought to elucidate whether Rmrp inhibited PFKFB3 expression through ZFP36 upregulation, thereby affecting glycolysis and immune tolerance in AMs. To investigate this, we treated AMs with exosomes derived from AEC‐IIs and assessed the expression levels of Rmrp, ZFP36, and PFKFB3 following the induction of the in vitro immune tolerance model (**Figure** **6**A). Compared with the sham group, AEC‐II‐derived exosomes from the CLP group significantly increased Rmrp and ZFP36 levels in AMs, while simultaneously reducing the mRNA and protein levels of PFKFB3 (Figures 6B,C; Figure S15A, Supporting Information). Notably, AEC‐II‐derived exosomes did not affect *Zfp36* mRNA levels in AMs (Figure 6B), suggesting that ZFP36 upregulation occurred independently of transcriptional regulation by exosomal Rmrp.

Next, we overexpressed or knocked down Rmrp and ZFP36 in MH‐S cells, followed by their exposure to an in vitro immune tolerance model (Figure 6D). ZFP36 silencing abolished Rmrp‐mediated decay of *Pfkfb3* mRNA, whereas ZFP36 overexpression prevented *Pfkfb3* mRNA stabilization induced by Rmrp knockdown (Figure 6E). Furthermore, ZFP36 knockdown reversed the Rmrp‐induced reduction in PFKFB3 levels, whereas ZFP36 overexpression diminished the increase in PFKFB3 levels observed upon Rmrp knockdown (Figures 6F; Figure S15B, Supporting Information). These data suggested that Rmrp mediates *Pfkfb3* mRNA degradation through ZFP36 upregulation, thereby regulating PFKFB3 expression.

To investigate the functional consequences of this pathway on immune responses and glycolysis, we assessed the effects of ZFP36 modulation in MH‐S cells. ZFP36 knockdown attenuated Rmrp‐induced impairment of immune responses and glycolysis (Figures 6G–J; Figure S15C–E, Supporting Information), whereas ZFP36 overexpression reduced the immune responses and glycolytic activity enhanced by Rmrp silencing (Figures 6G–J; Figure S15C–E, Supporting Information). These results supported the conclusion that the Rmrp/ZFP36 axis promotes immune tolerance and glycolytic defects in AMs.

Finally, we examined the role of PFKFB3 in the ZFP36‐mediated regulation of immune responses and glycolysis. We overexpressed or knocked down ZFP36 or PFKFB3 in MH‐S cells, followed by their exposure to an in vitro immune tolerance model (Figure 6K). ZFP36 overexpression significantly inhibited LPS‐induced production of TNF‐α, IL‐6, IL‐1β, and lactate (Figure 6L), along with reduced CD86 and MHC‐II expression (Figure 6M; Figure S15F, Supporting Information) and glycolysis (Figure 6N; Figure S15G–I, Supporting Information) in MH‐S cells. Notably, the immune tolerance and glycolytic impairment induced by ZFP36 overexpression were markedly attenuated by PFKFB3 overexpression (Figure 6L–N; Figure S15F–I, Supporting Information). Conversely, silencing PFKFB3 abolished the enhancement of immune responses and glycolysis in MH‐S cells caused by ZFP36 knockdown (Figure 6L–N; Figure S15F–I, Supporting Information). These findings indicated that ZFP36 induces immune tolerance and glycolytic defects in AMs by decreasing PFKFB3 expression.

### 2.7 S100A4/STAT3 Axis Promotes Rmrp Expression in AEC‐II‐Derived Exosomes After Sepsis

Our previous findings demonstrated that AEC‐II‐derived exosomal Rmrp suppresses immune function in AMs through the ZFP36/PFKFB3 axis. However, the mechanisms underlying the elevated expression of exosomal Rmrp in AEC‐IIs after sepsis remain unclear. In a previous study, we showed that the S100 calcium binding protein A4 (S100A4)/signal transducer and activator of transcription 3 (STAT3) axis is activated after sepsis and plays a critical role in exacerbating sepsis‐induced severe lung injury.^[^ ^48^ ^]^ However, their roles in SII have not been investigated. To address this, we examined the dynamics of the S100A4/STAT3 axis in AEC‐IIs at different time points post‐CLP. Both S100A4 expression and STAT3 phosphorylation significantly increased after CLP, peaking at 48 h post‐CLP (Figure S16A, Supporting Information).

Next, we explored whether the S100A4/STAT3 axis regulates Rmrp expression in AEC‐IIs. The overexpression of S100A4 (Figure S16B, C, Supporting Information) markedly increased p‐STAT3 levels in MLE‐12 cells (Figure S16C, Supporting Information), accompanied by an increase in both cellular and exosomal Rmrp levels (Figure S16D, Supporting Information). Conversely, silencing S100A4 (Figure S16B,C, Supporting Information) suppressed STAT3 phosphorylation in MLE‐12 cells (Figure S16C, Supporting Information), leading to a reduction in both cellular and exosomal Rmrp levels (Figure S16D, Supporting Information). Furthermore, overexpression or knockdown of STAT3 in MLE‐12 cells (Figure S16E, F, Supporting Information) led to corresponding increases or decreases in Rmrp levels in both cells and exosomes (Figure S16G, Supporting Information).

To determine the underlying mechanism, we used the JASPAR database to predict a potential binding site for STAT3 in the Rmrp promoter, specifically in the +1217 to +1226 sequence (Figure S16H, Supporting Information). Chromatin immunoprecipitation (ChIP) assays revealed significant enrichment of the P3 region of the Rmrp promoter (+1000 to +1600) in the anti‐STAT3 group compared to that in the IgG control group (Figure S16I, Supporting Information). Additionally, luciferase assays showed that STAT3 overexpression or knockdown promoted or inhibited the activity of luciferase linked to the WT Rmrp promoter, respectively, whereas the activity of luciferase linked to the MUT Rmrp promoter was unaffected by STAT3 (Figure S16J, Supporting Information). DNA pull‐down assays were performed to confirm the direct interaction between STAT3 and the *Rmrp* promoter. STAT3 was successfully pulled down by a biotinylated DNA probe containing the P3 region of the WT *Rmrp* promoter, whereas STAT3 did not bind to the MUT DNA probe, suggesting that STAT3 binds directly to the +1217 to +1226 sequences of the P3 region of the *Rmrp* promoter (Figure S16K, L, Supporting Information). In addition, inS3‐54A18, an inhibitor that specifically targets the DNA‐binding domain and inhibits the DNA‐binding activity of STAT3,^[^ ^49^ ^]^ significantly inhibited the binding of STAT3 to the *Rmrp* promoter, as demonstrated by the ChIP assay (Figure S16M, Supporting Information), further corroborating the direct interaction between STAT3 and the *Rmrp* promoter.

Further experiments were performed to verify whether the S100A4/STAT3 axis in AEC‐IIs regulates Rmrp expression and AM function. Upon overexpression of S100A4, Rmrp levels in the cellular and exosomal components of MLE‐12 cells were significantly upregulated (Figure S17A, Supporting Information), and MLE‐12 cell‐derived exosomes notably inhibited the secretion of pro‐inflammatory factors and lactate (Figure S17B, C, Supporting Information) as well as the glycolytic activity of AMs (Figure S17B, D–G, Supporting Information). When MLE‐12 cells were treated with STAT3 inhibitors (inS3‐54A18 or Stattic), S100A4‐mediated upregulation of cellular and exosomal Rmrp was abrogated (Figure S17A, Supporting Information), as was the exosome‐induced impairment of immune tolerance and glycolysis in AMs (Figure S17B–G, Supporting Information). Additionally, LPS induced cellular and exosomal Rmrp upregulation in MLE‐12 cells (Figure S18A, Supporting Information). Exosomes derived from LPS‐challenged MLE‐12 cells impaired immune responses and glycolysis in AMs (Figure S18B–G, Supporting Information), and these effects were abolished after S100A4 knockdown in MLE‐12 cells (Figure S18A–G, Supporting Information).

Taken together, these results suggested that the +1217 to +1226 sequences within the P3 region of the Rmrp promoter mediate STAT3‐driven transcriptional activation. The S100A4/STAT3 axis promotes AEC‐II‐derived exosomal Rmrp expression and enhances the inhibitory effect of AEC‐II‐derived exosomes on the immune responses and glycolysis of AMs following sepsis.

### 2.8 Rmrp Depletion in AEC‐IIs or AMs Enhances Immune Responses and Alleviates SII and Secondary Pneumonia

**Figure 7:**
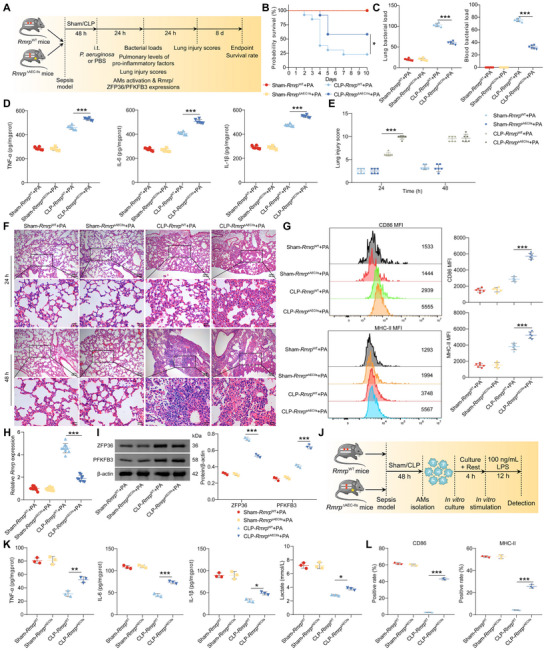
Rmrp depletion in AEC‐IIs or AMs enhances immune responses and alleviates SII and secondary pneumonia. A) Schematic overview of the experimental design for panels 7B–I. B) Survival rates of *Rmrp* ^WT^ and *Rmrp* ^∆AEC–IIs^ mice in the sham group or after CLP followed by *P. aeruginosa* challenge (n = 12/group). C) Bacterial loads in lung and blood samples from *Rmrp* ^WT^ and *Rmrp* ^∆AEC–IIs^ mice (n = 6/group). D) Measurement of TNF‐α, IL‐6, and IL‐1β levels in lung tissue samples (n = 6/group). F) Representative hematoxylin and eosin (HE) staining images of lung tissues. Scale bar: 100 µm. E) Lung injury scores based on HE staining (n = 6/group). G) Flow cytometry analysis of CD86 and MHC‐II expression in AMs from *Rmrp* ^WT^ and *Rmrp* ^∆AEC–IIs^ mice. Representative flow cytometry plots are shown (left). The MFI of CD86 and MHC‐II in AMs is plotted (right) (n = 6/group). H) RT‐qPCR analysis of Rmrp expression in AMs isolated from *Rmrp* ^WT^ and *Rmrp* ^∆AEC–IIs^ mice (n = 9/group). I) WB analysis of ZFP36 and PFKFB3 protein levels in AMs isolated from *Rmrp* ^WT^ and *Rmrp* ^∆AEC–IIs^ mice. Densitometric analysis of ZFP36 and PFKFB3 immunoblots is shown (right) (n = 3/group). J) Schematic overview of the experimental design for panels 7K, L, and S20A–C. (K) ELISA of TNF‐α, IL‐6, IL‐1β, and lactate concentrations in the supernatant of AMs isolated from *Rmrp* ^WT^ or *Rmrp* ^∆AEC–IIs^ mice and treated with LPS (n = 3/group). L) Percentages of CD86^+^ and MHC‐II^+^ AMs in LPS‐treated AMs from *Rmrp* ^WT^ and *Rmrp* ^∆AEC–IIs^ mice, as determined by flow cytometry (n = 3/group). Data are presented as mean ± SD. Statistical analysis was performed using the Log‐rank test (B), one‐way ANOVA followed by Tukey's post hoc test (C, D, G, H, K, and L), and two‐way ANOVA followed by Sidak's test (E, I). ^*^ *p* < 0.05, ^**^ *p* < 0.01, ^***^ *p* < 0.001.

To investigate the impact of AEC‐II‐derived Rmrp on AM glycolysis, SII, and secondary pneumonia in vivo, we generated AEC‐II‐specific *Rmrp* knockout (*Rmrp* ^∆AEC–IIs^) mice by crossing Rmrp floxed mice (*Rmrp* ^fl/fl^) with *Sftpc*‐Cre mice (*Sftpc* ^Cre^) (Figure S19A, B, Supporting Information). *Rmrp* ^WT^ (*Rmrp* ^fl/fl^) and *Rmrp* ^∆AEC–IIs^ (*Sftpc* ^Cre^;*Rmrp* ^fl/fl^) mice were subjected to CLP followed by intratracheal infection with *Pseudomonas aeruginosa* on day 2 after CLP (**Figure** **7**A). Compared to *Rmrp* ^WT^ mice, septic *Rmrp* ^∆AEC–IIs^ mice infected with *P. aeruginosa* exhibited significantly lower mortality (Figure 7B) and improved bacterial clearance from the lungs and blood (Figure 7C). Additionally, pulmonary levels of pro‐inflammatory factors (Figure 7D) and lung injury scores (Figure 7E,F) were markedly elevated in *Rmrp* ^∆AEC–IIs^ mice compared to *Rmrp* ^WT^ mice following secondary infection, indicating stronger lung immune responses in *Rmrp* ^∆AEC–IIs^ mice.

Furthermore, compared with *Rmrp* ^WT^ mice, AMs from *Rmrp* ^∆AEC–IIs^ mice exhibited increased expression of CD86 and MHC‐II (Figure 7G), accompanied by reduced Rmrp (Figure 7H) and ZFP36 levels, and higher PFKFB3 expression (Figure 7I) after *P. aeruginosa* challenge post‐CLP. We isolated AMs from septic *Rmrp* ^WT^ and *Rmrp* ^∆AEC–IIs^ mice and established an in vitro immune tolerance model (Figure 7J). Compared to *Rmrp* ^WT^ mice, AMs from septic *Rmrp* ^∆AEC–IIs^ mice exhibited enhanced immune responses (Figure 7K, L; Figure S20A, Supporting Information), reduced Rmrp (Figure S20B, Supporting Information) and ZFP36 (Figure S20C, Supporting Information) expression, and increased lactate production (Figure 7K) and PFKFB3 expression (Figure S20C, Supporting Information) in response to LPS challenge in vitro. These results suggest that Rmrp depletion in AEC‐IIs leads to stronger immune responses in both the lungs and AMs, ameliorating SII and providing protection against *P. aeruginosa*‐induced secondary pneumonia after sepsis.

To further investigate the role of exosomal Rmrp in the prognosis of SII and secondary pneumonia, we collected exosomes from in vitro‐cultured MLE‐12 cells and administered them intratracheally to sham and CLP mice, which were subsequently infected with *P. aeruginosa* 2 days after CLP (Figure S21A, Supporting Information). MLE‐12‐derived exosomes decreased the survival rate of septic mice with secondary infections (Figure S21B, Supporting Information) and increased the bacterial load in the lungs and blood (Figure S21C, Supporting Information). Additionally, MLE‐12‐derived exosomes downregulated lung tissue inflammatory factor levels (Figure S21D, Supporting Information) and lung injury scores (Figure S21E, F, Supporting Information) and suppressed CD86 and MHC‐II expression in AMs of *P. aeruginosa*‐challenged septic mice (Figure S21G, H, Supporting Information). Notably, the effects of MLE‐12‐derived exosomes were abolished when Rmrp was knocked down (Figure S21B–H, Supporting Information).

Next, we isolated AMs from mice that were intratracheally injected with MLE‐12‐derived exosomes and exposed to an in vitro immune tolerance model (Figure S22A, Supporting Information). AMs from CLP mice treated with MLE‐12‐derived exosomes secreted fewer pro‐inflammatory factors and lactate upon in vitro LPS stimulation (Figure S22B, Supporting Information) and expressed lower levels of CD86 and MHC‐II than those in the CLP group (Figure S22C, D, Supporting Information). However, the immune functions of AMs from CLP mice treated with Rmrp‐silenced MLE‐12‐derived exosomes were comparable to those of AMs from CLP mice (Figure S22B–D, Supporting Information). These results suggest that AEC‐II‐derived exosomal Rmrp exacerbates immune tolerance in AMs and promotes secondary infection after sepsis.

To further investigate the effects of Rmrp depletion on AM immune function, SII, and the prognosis of secondary pneumonia, we intratracheally injected mice with a macrophage‐specific adeno‐associated virus (AAV) harboring a synthetic F4/80 promoter, an Rmrp‐specific shRNA, and a cytomegalovirus promoter (Figure S23A, Supporting Information). Two weeks post‐injection, the mice were subjected to CLP and *P. aeruginosa* infection at 48 h post CLP for secondary infection (Figure S23A, Supporting Information). AAV‐F4/80‐sh‐Rmrp was successfully delivered to AMs and specifically reduced Rmrp expression in these cells (Figure S23B, C, Supporting Information). Compared to the AAV‐F4/80‐sh‐NC group, AAV‐F4/80‐sh‐Rmrp‐treated mice exhibited significantly lower mortality (Figure S23D, Supporting Information) and reduced bacterial loads in the lungs and blood (Figure S23E, Supporting Information) after *P. aeruginosa* infection. Additionally, AAV‐F4/80‐sh‐Rmrp treatment promoted the expression of pro‐inflammatory factors (Figure S23F, Supporting Information) and increased lung pathology scores (Figure S23G, H, Supporting Information), indicating enhanced immune responses in the lungs. Furthermore, AMs from septic mice treated with AAV‐F4/80‐sh‐Rmrp showed decreased Rmrp (Figure S23K, Supporting Information) and ZFP36 (Figure S23L, Supporting Information), whereas CD86, MHC‐II (Figure S23I, J, Supporting Information), and PFKFB3 (Figure S23L, Supporting Information) expression levels were elevated compared to the AAV‐F4/80‐sh‐NC group.

To further explore the effects of AAV‐F4/80‐sh‐Rmrp on immune function and glycolysis in AMs, we isolated AMs from CLP mice injected with either AAV‐F4/80‐sh‐NC or AAV‐F4/80‐sh‐Rmrp and established an in vitro immune tolerance model (Figure S24A, Supporting Information). Compared to the AAV‐F4/80‐sh‐NC group, AMs from AAV‐F4/80‐sh‐Rmrp‐injected mice secreted more pro‐inflammatory factors (Figure S24B, Supporting Information) and expressed higher levels of CD86, MHC‐II (Figure S24C, D, Supporting Information), and PFKFB3 (Figure S24F, Supporting Information); however, they expressed lower levels of Rmrp (Figure S24E, Supporting Information) and ZFP36 (Figure S24F, Supporting Information) following LPS stimulation. These results suggest that Rmrp depletion enhanced the immune responses and glycolysis in AMs, thereby alleviating SII and secondary pneumonia.

Furthermore, to validate the therapeutic feasibility of the exosome secretion inhibitor (GW4869) in animal models, we administered sham and CLP mice with GW4869 intraperitoneally, followed by *P. aeruginosa* secondary infection (Figure S25A, Supporting Information). GW4869 notably increased the survival of *P. aeruginosa*‐challenged septic mice (Figure S25B, Supporting Information) and decreased the bacterial load in the lungs and blood (Figure S25C, Supporting Information). GW4869 increased the pulmonary inflammatory factor levels (Figure S25D, Supporting Information) and lung injury scores (Figure S25E, F, Supporting Information) in septic mice with *P. aeruginosa* secondary infection. GW4869 also promoted the activation and PFKFB3 expression in AMs and inhibited the expression of ZFP36 and Rmrp (Figure S25G–I, Supporting Information). To further explore the effects of GW4869 on immune function and glycolysis in AMs after sepsis, we isolated AMs from CLP mice and exposed them to an in vitro immune tolerance model (Figure S25J, Supporting Information). Compared to the CLP group, AMs from GW4869‐treated CLP mice secreted more pro‐inflammatory factors and lactate (Figure S25K, Supporting Information) and expressed more PFKFB3 and less ZFP36 after LPS stimulation in vitro (Figure S25L, Supporting Information). These data suggest a potential therapeutic role for GW4869 in AM immune tolerance and secondary infections after sepsis.

### 2.9 Exosomal Rmrp Correlates with AM Immune Tolerance and Prognosis in Patients with Sepsis

**Figure 8:**
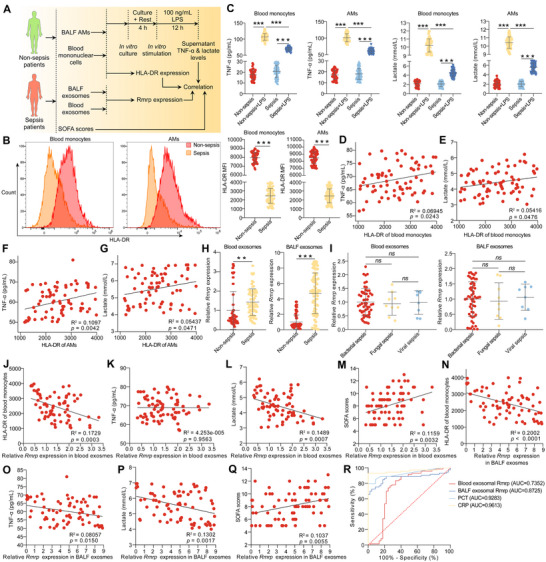
Exosomal Rmrp correlates with AM immune tolerance and prognosis of patients with sepsis. A) Schematic overview of the experimental design for 8B–R. B) Flow cytometry analysis of HLA‐DR expression in monocytes and BALF AMs. Representative flow cytometry plots are shown (left). The MFI of HLA‐DR in monocytes and BALF AMs is plotted (right) (n = 45 and 73 for non‐sepsis and sepsis group, respectively). C) ELISA of TNF‐α (left) and lactate (right) concentrations in the supernatant of monocytes and BALF AMs following LPS stimulation in vitro (n = 45 and 73 for non‐sepsis and sepsis group, respectively). Correlation analysis between HLA‐DR level with D) TNF‐α or E) lactate production in monocytes after LPS treatment in vitro (n = 73). Correlation analysis between HLA‐DR level with F) TNF‐α or G) lactate production in BALF AMs after LPS stimulation (n = 73). H) RT‐qPCR analysis of Rmrp levels in exosomes isolated from serum (left) and BALF (right) of patients with or without sepsis (n = 45 and 73 for non‐sepsis and sepsis group, respectively). I) Exosomes were isolated from serum (left) and BALF (left) of patients with sepsis infected with bacteria, fungi, or viruses. RT‐qPCR was used to assess the expression of Rmrp (n = 56, 9, and 8 for bacterial, fungal, and viral sepsis groups, respectively). Correlation analysis between blood exosomal Rmrp levels with J) HLA‐DR expression in monocytes, K) TNF‐α or L) lactate production in monocytes after LPS treatment in vitro, and M) SOFA scores of patients with sepsis (n = 73). Correlation analysis between BALF exosomal Rmrp levels with N) HLA‐DR expression in AMs, O) TNF‐α or P) lactate production in AMs after LPS treatment in vitro, and Q) SOFA scores of patients with sepsis (n = 73). R) ROC curve analysis of the diagnostic efficiency of blood exosomal Rmrp, BALF exosomal Rmrp, PCT, and CRP for sepsis. Data are presented as mean ± SD. Statistical analysis was performed using Student's *t*‐test (B, H), one‐way ANOVA followed by Tukey's post hoc test (C, I), and Pearson correlation analysis (D–G, J–Q). ^**^ *p* < 0.01, ^***^ *p* < 0.001, ns: not significant.

Previous studies have elucidated the pivotal role of AEC‐II‐derived exosomal Rmrp in the regulation of glycolysis and immune functions in AMs following sepsis. To further investigate whether exosomal Rmrp correlates with immune tolerance in AMs and the prognosis of patients with sepsis, we collected peripheral blood and BALF from patients with and without sepsis and isolated serum and BALF exosomes as well as peripheral blood monocytes and BALF AMs for further detection (**Figure** **8**A). Compared with patients without sepsis, peripheral blood monocytes and BALF AMs from patients with sepsis exhibited lower human leukocyte antigen‐D related (HLA‐DR) expression (Figure 8B) and lower TNF‐α and lactate production after in vitro LPS stimulation (Figure 8C). Concurrently, HLA‐DR expression in monocytes or AMs from patients with sepsis was positively correlated with TNF‐α and lactate synthesis after LPS treatment in vitro (Figure 8D–G). In addition, exosomal Rmrp levels were strikingly upregulated in the serum and BALF after sepsis (Figure 8H), although no significant differences in exosomal Rmrp levels were observed among the viral, bacterial, and fungal sepsis groups (Figure 8I). Furthermore, exosomal Rmrp levels in patients with sepsis were negatively correlated with HLA‐DR expression of monocytes or AMs and TNF‐α or lactate production after LPS stimulation in vitro (Figure 8J–L,N–P), whereas they were positively correlated with patients’ sequential organ failure assessment (SOFA) scores (Figure 8M,Q). Moreover, we evaluated the diagnostic efficacy of exosomal Rmrp for sepsis using receiver operating characteristic (ROC) curve analysis. ROC analysis showed that serum exosomal Rmrp and BALF exosomal Rmrp were effective in differentiating patients with sepsis from non‐septic controls (Figure 8R). Although not as good as procalcitonin (PCT) or C‐reactive protein (CRP), exosomal Rmrp still showed good diagnostic performance for sepsis (Figure 8R), suggesting that exosomal Rmrp may serve as a potential biomarker for sepsis diagnosis.

Collectively, these data indicate that exosomal Rmrp is closely associated with AM immune tolerance and the prognosis of patients with sepsis, highlighting its potential as a biomarker of immune dysfunction and disease severity in sepsis.

## 3 Discussion

This study highlights a previously unrecognized role of AEC‐II‐derived exosomal Rmrp in regulating AM glycolysis and immune function after sepsis. Our findings demonstrate that exosomal Rmrp secreted by AEC‐IIs inhibits glycolytic activity and pro‐inflammatory responses in AMs, exacerbating SII and secondary pneumonia. Mechanistically, Rmrp promoted *Pfkfb3* mRNA degradation by upregulating ZFP36, an RBP that destabilizes *Pfkfb3* transcripts. Rmrp stabilizes ZFP36 and inhibits its ubiquitination. Importantly, the depletion of Rmrp in either AEC‐IIs or AMs restored AM glycolysis, enhanced immune responses, improved bacterial clearance, and mitigated *P. aeruginosa*‐induced secondary pneumonia in septic mice. Furthermore, elevated levels of exosomal Rmrp in the serum and BALF correlated with impaired AM immune function and poor prognosis in patients with sepsis, suggesting its potential as a biomarker for disease severity and progression.

Sepsis is characterized by complex immune dysregulation, transitioning from an initial hyperinflammatory phase to a state of profound immunosuppression, which can persist long after hospital discharge.^[^ ^50^ ^]^ This immunosuppressive state renders patients highly susceptible to secondary infections, recurrent inflammation, and prolonged organ dysfunction, collectively known as persistent inflammation, immune suppression, and catabolism syndrome.^[^ ^51^, ^52^ ^]^ Secondary pneumonia is one of the most common clinical manifestations of SII, driven primarily by dysfunctional AMs, which are essential for lung immune homeostasis, pathogen clearance, and resolution of inflammation.^[^ ^53^, ^54^ ^]^ Although glycolysis has been established as the central metabolic pathway supporting AM immune responses, its disruption after sepsis significantly impairs macrophage function.^[^ ^55^, ^56^ ^]^ Our study contributes to the growing body of knowledge by identifying AEC‐IIs as key modulators of AM glycolysis through the secretion of exosomal Rmrp. These findings underscore the importance of the alveolar microenvironment in shaping AM metabolism and immune responses during sepsis.

LncRNAs have emerged as critical regulators of immune and metabolic pathways. Rmrp has been implicated in developmental processes, autoimmune diseases, and malignancies,^[^ ^57^, ^58^, ^59^, ^60^, ^61^ ^]^ but its role in immune cells, particularly in the context of sepsis, remains unexplored. Here, we demonstrated that Rmrp levels were significantly elevated in AEC‐IIs, AMs, and BALF exosomes after sepsis. Gain‐ and loss‐of‐function experiments revealed that Rmrp is a key mediator of AM glycolytic defects and immune tolerance via its effects on PFKFB3, a rate‐limiting enzyme in glycolysis. While previous studies have identified upstream regulators of PFKFB3, such as HIF‐1α and NLRP3,^[^ ^62^, ^63^, ^64^ ^]^ our data highlight a novel regulatory axis in which Rmrp promotes ZFP36‐mediated degradation of *Pfkfb3* mRNA. This axis links lncRNA signaling to macrophage metabolism and immune function, offering new insights into the regulation of macrophage bioenergetics in SII.

Previous studies have confirmed that Rmrp affects RBP function. In platinum‐resistant ovarian cancer, Rmrp promotes mRNA translation by recruiting DEAD‐Box Helicase 3 X‐Linked to enrich *PHGDH* mRNA.^[^ ^65^ ^]^ In temozolomide‐resistant glioma, Rmrp interacts with insulin‐like growth factor 2 mRNA‐binding protein 3 (IGF2BP3) and *ZNRF3* mRNA to inhibit IGF2BP3‐mediated *ZNRF3* mRNA stability.^[^ ^61^ ^]^ Rmrp affects metabolic processes through a competitive endogenous RNA mechanism. Rmrp sponges miR‐206 to deregulate its inhibition of protein tyrosine phosphatase non‐receptor type 1 (PTPN1), activating the PTPN1–sterol regulatory element binding transcription factor 1c pathway, upregulating fatty acid synthase expression, and exacerbating lipid accumulation in the liver.^[^ ^66^ ^]^ In tumor metabolic reprogramming, Rmrp upregulates mitochondrial calcium uptake 1 by sponging miR‐580‐3p, which in turn accelerates ATP synthesis and glycolysis and drives ovarian cancer chemoresistance.^[^ ^67^ ^]^ In the present study, we demonstrated that Rmrp stabilizes ZFP36 by binding to ZFP36, which, in turn, promotes ZFP36‐mediated *Pfkfb3* mRNA degradation and glycolytic inhibition, elucidating the critical role of the Rmrp/ZFP36/PFKFB3 axis in modulating macrophage immune functions and glycolysis.

In addition to the AEC‐II‐derived exosomal Rmrp, other exosomal RNAs and AEC‐II‐derived factors affect AM function. AEC‐II‐derived exosomal miR‐223 and miR‐27b‐3p regulate pro‐ and anti‐inflammatory, damage‐ and repair‐associated, and pro‐ and anti‐fibrotic phenotypes of Ams.^[^ ^68^ ^]^ Feng et al. revealed that AEC‐IIs influence glycolysis in tissue‐resident AMs and further affect the prognosis of ARDS and IPF through the exosomal STIM‐activating enhancer protein.^[^ ^31^ ^]^ Moreover, exosomal miR‐30d‐5p^[^ ^69^ ^]^ MEG3,^[^ ^70^ ^]^ miR‐331,^[^ ^71^ ^]^ and other miRNAs are involved in regulating macrophage function. Although the disease models studied above are not directly related to SII, given its complexity, it is likely that these exosomal RNAs and AEC‐II‐derived factors are aberrantly expressed after SII and regulate AM dysfunction. Further exploration of factors beyond AEC‐II‐derived exosomal Rmrp is required to gain a clearer understanding of the mechanisms underlying immunomodulation after SII.

Beyond providing mechanistic insights, our study has important clinical implications. Elevated exosomal Rmrp levels in serum and BALF were strongly associated with impaired AM immune responses and poor clinical outcomes in patients with sepsis, suggesting that exosomal Rmrp could serve as a biomarker for SII and secondary pneumonia. Although current biomarkers for sepsis primarily focus on systemic inflammation, exosomal Rmrp reflects a more specific aspect of immune dysfunction at the cellular and microenvironmental levels, potentially providing a more targeted approach for patient stratification and management.

In summary, we identified the AEC‐II‐derived exosomal Rmrp as a critical regulator of glycolytic defects and immune tolerance in AMs following sepsis. By promoting ZFP36‐mediated degradation of *Pfkfb3* mRNA, Rmrp inhibits PFKFB3 expression, thereby impairing AM immune function and driving immunosuppression. Elevated exosomal Rmrp levels correlate with poor prognosis in patients with sepsis, highlighting its potential as both a biomarker and a therapeutic target. Targeted inhibition of Rmrp in AEC‐II or AMs offers a promising strategy for alleviating SII and preventing secondary pneumonia, paving the way for novel therapeutic interventions in sepsis management.

## 4 Experimental Section

### Mice

C57BL/6J mice (22–28 g, 8–10 weeks old) were purchased from Hunan SJA Laboratory Animal Co., Ltd. Rmrp floxed mice (*Rmrp* ^fl/fl^) with a C57BL/6J background, harboring loxP sequences flanking exon 1 of the *Rmrp*‐202 transcript (ENSMUST00000226420.2), were generated by GemPharmatech (Shanghai, China) using CRISPR–Cas9 technology (Strain #T066710). Rmrp floxed mice were crossed with *Sftpc*‐Cre mice (Strain #T004715, GemPharmatech, Shanghai, China) to obtain AEC‐II‐specific Rmrp conditional knockout mice (*Sftpc* ^Cre^;*Rmrp* ^fl/fl^). All the mice were housed in a specific pathogen‐free environment and provided with standard laboratory food and sterile water. The animal room was maintained at 22 ± 2 °C with a 12 h light/dark cycle. All animal procedures were approved by the Experimental Animal Ethics Committee of the University of South China (Approval #2024491) and conducted in accordance with the institutional guidelines.

### Cell Culture and Treatment

Primary AEC‐IIs^[^ ^72^ ^]^ and AMs^[^ ^73^ ^]^ were isolated as described previously. For AEC‐II isolation, the mice were sacrificed, and after skin disinfection, the lungs and heart were exposed via the chest cavity. A 25G needle was inserted into the right ventricle for DPBS instillation until the lungs turned white. Following tracheal exposure, a 20G angiocatheter was inserted, and dispase (Sigma‐Aldrich, #4942078001) and 1% low‐melt agarose (Yeasen, #10214ES25) were sequentially instilled. The lungs were covered with ice to allow the agarose to solidify. Lung tissues were isolated, washed, and digested with dispase, followed by incubation with DMEM (Gibco, #11965092) or DNase (Sigma–Aldrich, #D5025). A single‐cell lung suspension was obtained through filtration and incubated with biotin‐labeled antibodies against anti‐CD45 (eBioscience, #13‐0451‐85), anti‐CD16/32 (BD Biosciences, #553143), anti‐CD31 (BioLegend, #102504), anti‐Ter119 (eBioscience, #13‐5921‐85), and anti‐integrin β4 (BioLegend, #12603). Lineage‐positive cells were removed using magnetic bead sorting, and lineage‐negative cells were further incubated with anti‐EpCAM‐APC (eBioscience, #17‐5791‐82), followed by flow cytometry to isolate EpCAM+ AEC‐IIs. AEC‐IIs were cultured in keratinocyte‐SFM (Gibco, #17005042) supplemented with 1 mM isoproterenol (Selleck, #S2566), 10 µg mL^−1^ fibronectin (Sorlabio, #F8180), 30 µg mL^−1^ vitamins (Procell, #PB180426), and 10 µg mL^−1^ bovine serum albumin (BSA; Sorlabio, #H1130).

For AM isolation, BALF was incubated with anti‐CD45‐FITC (BioLegend, #157607), anti‐CD11c‐APC (BioLegend, #117309), and anti‐SiglecF‐PE (BioLegend, #155505). AMs (CD45^+^CD11c^+^SiglecF^+^) were isolated using flow cytometry and were cultured in DMEM containing 10% FBS, 100 U mL^−1^ recombinant M‐CSF (PeproTech, #315‐02), 1 mM sodium pyruvate (Solarbio, #P8380), 10 mM HEPES (Solarbio, #H8090), and 1% penicillin–streptomycin (GE Healthcare Life Sciences, #SV30010).

For other cell types, NK cells (CD3^−^ NK1.1^+^), neutrophils (Ly‐6G^+^), and AEC‐Is (T1α^+^) were isolated from the lung single‐cell suspension using flow cytometry. Antibodies used include anti‐CD3‐FITC (BD Biosciences, #561798), anti‐NK1.1‐APC (BD Biosciences, #561117), anti‐Ly‐6G‐APC (BioLegend, #127613), and anti‐T1α‐FITC (BioLegend, #127415).

MH‐S cells (Changsha Abiowell Biotechnology, #AW‐CNM405) and MLE‐12 cells (Shanghai Zhong Qiao Xin Zhou Biotechnology, #ZQ0470) were cultured in RPMI‐1640 (Procell, #PM150110) with 10% FBS and 1% penicillin–streptomycin and DMEM with 10% FBS and 1% penicillin–streptomycin, respectively. For knockdown or overexpression experiments, shRNA and overexpression plasmids were constructed by HonorGene (Changsha, China) and transfected using Lipofectamine 3000 reagent (Invitrogen, #L3000150) according to the manufacturer's protocols.

An in vitro immune tolerance model for AMs and MH‐S cells was established as previously described.^[^ ^16^ ^]^ Briefly, AMs or MH‐S cells were cocultured with CM or exosomes. After washing with PBS, the cells were cultured in fresh medium and allowed to rest for 4 h. Subsequently, the cells were re‐challenged with 100 ng mL^−1^ LPS (Selleck, #S7850) for 12 h. MLE‐12 cells were stimulated with LPS (100 ng mL^−1^) for 24 h.

### Sepsis and Secondary Pneumonia Model

A CLP‐induced sepsis model was established as previously described.^[^ ^74^ ^]^ Briefly, the mice were anesthetized, and their abdominal hair was removed, followed by skin disinfection. A midline abdominal incision was made, and the abdominal muscles were bluntly separated to expose the abdominal cavity. The cecum was then externalized, and 50% of the cecum was ligated using surgical sutures. A 21G hollow‐bore needle was used to puncture the ligated cecum and extract small amounts of feces. Subsequently, the cecum was returned to the abdominal cavity. Mice in the sham group underwent the same procedure, excluding the cecal puncture. After the abdominal cavity was closed, 1 mL of 37 °C saline was injected intraperitoneally for resuscitation. The mice were returned to their cages with ad libitum access to food and water.

For exosome‐injection experiments, MLE‐12 cell‐derived exosomes (7.5 µg g^−1^ body weight) were suspended in 20 µL of PBS and intratracheally administered to mice once per day. To model secondary pneumonia, mice were anesthetized on day 2 post‐CLP and intratracheally injected with 20 µL (3 × 10⁹ CFU mL^−1^) of *P. aeruginosa* as described previously.^[^ ^16^ ^]^ Mice in the control group received 20 µL of PBS intratracheally. The mice were euthanized at various time points following CLP or *P. aeruginosa* challenge, and lung and blood samples were collected for further analysis.

To inhibit Rmrp in AMs, Hanbio Biotechnology Co., Ltd. (Shanghai, China) was commissioned to construct a macrophage‐specific AAV containing a synthetic F4/80 promoter, an Rmrp shRNA sequence, and a cytomegalovirus promoter. Mice were intratracheally injected with AAV‐F4/80‐sh‐NC or AAV‐F4/80‐sh‐Rmrp at a dose of 1 × 10^11^ viral particles in 20 µL per mouse. CLP was performed 28 days after the injection.

### ELISA

Cell culture supernatants were collected and centrifuged at 1000 ×*g* for 15 min to remove cellular debris. Lung tissue samples were homogenized, and the homogenates were centrifuged at 5000 ×*g* for 5 min to collect the supernatant. Concentrations of TNF‐α (Proteintech, #KE10002), IL‐6 (Proteintech, #KE10007), IL‐1β (Proteintech, #KE10003), and lactate (Nanjing Jiancheng Bioengineering Institute, #A019‐2‐1) were measured in these samples using commercial ELISA kits, following the manufacturer‘s instructions.

### Flow Cytometry

The cells were harvested after the different treatments and incubated with anti‐CD86 (eBioscience, #25‐0862‐82), anti‐MHC‐II (eBioscience, #25‐5321‐82), or anti‐HLA‐DR (eBioscience, #11‐9956‐42) antibodies for 15 min at room temperature in the dark. After incubation, cells were washed with PBS and centrifuged at 300 ×*g* for 5 min. The supernatant was discarded, and the cells were resuspended in PBS for flow cytometric analysis. Data were acquired using a flow cytometer and analyzed using the FlowJo software (BD, USA).

### Seahorse Extracellular Flux Analysis

Seahorse extracellular flux analysis was performed to determine the ECAR of AMs and MH‐S cells. Briefly, cells were cultured overnight in Seahorse XF culture plates, whereas sensor probe plates were hydrated overnight at 37 °C in a CO_2_‐free environment. On the day of the experiment, the medium was replaced with buffer‐free, bicarbonate‐free Seahorse XF assay medium and equilibrated for 1 h at 37 °C in a CO_2_‐free incubator. The test compounds (glucose, oligomycin, and 2‐DG) were sequentially preloaded onto the probe‐plate drug injection port. The equilibrated cell plates and loaded probe plates were placed in the Seahorse XF analyzer, and the instrument automatically executed the program. Baseline ECAR measurements were performed first, followed by injection of drugs in a preset order, and multiple cycles were performed after each injection to monitor changes in cellular glycolytic flux in real time. The entire experiment was performed at a constant temperature of 37 °C.

### Detection of Glucose Intake, G6P, and Pyruvate

The AMs and MH‐S cells were subjected to various treatments. The glucose uptake assay kit (Beyotime, #S0554S), G6P assay kit (Beyotime, #S0185), and Amplex Red pyruvate assay kit (Beyotime, #S0299S) were used to measure the glucose uptake, G6P levels, and pyruvate levels, respectively, following the manufacturer's instructions.

### Exosome Isolation, Identification, and Quantification

Exosomes were isolated using ultracentrifugation as previously described.^[^ ^75^ ^]^ Briefly, the collected cell supernatant, BALF, or serum was first centrifuged at 300 ×*g* for 10 min at 4 °C to remove cellular debris. The supernatant was then centrifuged at 2000 ×*g* for 10 min at 4 °C, followed by a 30 min centrifugation at 10000 ×*g* at 4 °C. Afterward, the supernatant was centrifuged at 100 000 ×*g* for 70 min at 4 °C to pellet the exosomes. The pellet was resuspended in PBS and subjected to another ultracentrifugation at 100 000 ×*g* for 70 min at 4 °C to further purify the exosomes. The resulting exosomes were either stored at −80 °C or resuspended in PBS for subsequent use in cell treatments.

Exosome morphology was examined using TEM. Exosomes were deposited onto a copper grid and stained with 2% phosphotungstic acid, followed by examination under TEM. NTA was used to assess the particle size distribution and concentration of exosomes using NanoSight NS300 (Malvern Instruments, UK). WB analysis was performed to detect the expression of exosomal markers, including CD9, CD63, CD81, TSG101, and Alix, as well as the endoplasmic reticulum marker GRP94.

### WB Analysis

The cells or exosomes were lysed using RIPA lysis buffer (Abiowell, #AWB0136). The protein concentrations were determined using a bicinchoninic acid protein assay kit (Abiowell, #AWB0104). Proteins were separated using sodium dodecyl sulfate‐polyacrylamide gel electrophoresis and transferred to polyvinylidene fluoride membranes (Bio‐Rad, #1620177). Membranes were blocked with 5% BSA (Solarbio, #SW3015) and incubated overnight at 4 °C with primary antibodies. The antibodies used were anti‐CD9 (Abcam, #ab307085), anti‐CD63 (Abcam, #ab315108), anti‐CD81 (Abcam, #ab109201), anti‐TSG101 (Proteintech, #28283‐1‐AP), anti‐Alix (Abcam, #ab275377), anti‐GRP94 (Proteintech, #14700‐1‐AP), and other metabolic and immune‐related markers, such as anti‐HK1 (, #19662‐1‐AP), anti‐PFKFB3 (Proteintech, #13763‐1‐AP), and anti‐ZFP36 (Proteintech, #12737‐1‐AP). After incubation with the primary antibodies, the membranes were incubated with horseradish peroxidase‐conjugated secondary antibodies (Proteintech, #SA00001‐2) at room temperature for 1 h. Signals were visualized using chemiluminescence reagents (Abiowell, #AWB0005), and images were acquired and analyzed.

### Fluorescence Labeling and Tracing of Exosomes or Exosomal RNAs

Exosomes were labeled with the PKH67 dye (Solarbio, #D0031) by incubating them with the dye for 20 min at 37 °C, protected from light. Following incubation, the exosomes were washed with PBS and centrifuged at 10 000 ×*g* for 70 min at 4 °C. The supernatant was discarded, and PKH67‐labeled exosomes were resuspended in PBS before being added to the culture medium of AMs. For exosomal RNA tracing, Cy3‐labeled Rmrp (HonorGene, China) was transfected into MLE‐12 cells. Exosomes were isolated via ultracentrifugation and subsequently used to treat AMs. AMs treated with the labeled exosomes were fixed with 4% paraformaldehyde (Beyotime, #P0099) and permeabilized with 0.1% Triton X‐100 (Beyotime, #P0096). After nuclear staining with 4′,6‐diamidino‐2‐phenylindole (Biosharp, #BS097), the fluorescence intensity in AMs was observed under a fluorescence microscope to assess the uptake of exosomes or exosomal Rmrp.

### RT‐qPCR

Total RNA was extracted from cells, exosomes, or supernatants using TRIzol reagent (Thermo Fisher Scientific, #15596026). RNA was then reverse transcribed into cDNA using a HiFiScript cDNA Synthesis Kit (CWBiotech, #CW2569), according to the manufacturer's instructions. qPCR was performed on a PCR instrument (Thermo Fisher Scientific, #PIKOREAL96) using the UltraSYBR Mixture (CWBbiotech, #CW2601). mRNA levels or cellular lncRNA levels were normalized to β‐actin, whereas exosomal or supernatant lncRNA levels were normalized to the exogenous cel‐miR‐39‐3p control. The 2^−ΔΔCt^ method was used to calculate relative gene expression. Primers used for the assays are listed in Table S1 (Supporting Information).

### RIP Assay

RIP assays were performed as previously described.^[^ ^76^ ^]^ Briefly, cells were lysed using cold polysome lysis buffer (100 mm KCl, 5 mm MgCl_2_, 10 mM HEPES–NaOH, pH 7.0, and 0.5% NP‐40) supplemented with protease inhibitors, RNase inhibitors, and dithiothreitol. Protein A/G magnetic beads (GenScript, #L00277) were resuspended in 100 µL NT‐2 buffer (50 mm Tris‐HCl, pH 7.4, 150 mm NaCl, 1 mm MgCl_2_, 0.05% NP‐40) and incubated with anti‐PFKFB3, anti‐ZFP36, anti‐MBNL1, anti‐Flag, or IgG antibodies for 1 h at room temperature with rotation. After centrifugation and washing, the antibody‐coated beads were resuspended in NT‐2 buffer and incubated with cell lysates at 4 °C overnight. The following day, the samples were placed on a magnetic separation rack, and the supernatant was removed. The beads were washed with cold NT‐2 buffer and resuspended. Protein complexes were eluted using Proteinase K buffer, and the expression levels of Rmrp or *Pfkfb3* mRNA in the eluates were assessed using RT‐qPCR, as described previously.

### RNA Pull‐Down Assay

In vitro transcription of Rmrp or *Pfkfb3* mRNA fragments was performed using the MEGAscript T7 Transcription Kit (Invitrogen, #AM1333). RNA biotinylation was performed using the RNA 3′‐End Biotinylation Kit (Thermo Fisher Scientific, #20160). Biotinylated RNA was captured using streptavidin magnetic beads, according to the manufacturer's instructions for the Pierce Magnetic RNA–Protein Pull‐Down Kit (Thermo Fisher Scientific, #20164). The captured RNA was incubated with cell lysates at 4 °C for 6 h. Following incubation, the protein complexes were eluted for MS or WB analysis of ZFP36 or Flag‐ZFP36.

### RNA Stability Assay

To assess mRNA stability, cells were treated with 5 µg mL^−1^ actinomycin D (Selleck, #S8964) to inhibit endogenous RNA synthesis. Cell samples were collected at 0, 2, 4, 6, and 8 h after Actinomycin D treatment, and the expression levels of *Pfkfb3* or luciferase mRNA were determined using RT‐qPCR, as described previously, to evaluate mRNA stability.

### Polyubiquitination Analysis

Polyubiquitination was analyzed as described previously.^[^ ^77^ ^]^ Briefly, cells were pre‐treated with 10 µm proteasome inhibitor MG132 (Selleck, #S2619) for 6 h before sample collection. Cells were lysed using RIPA buffer containing 10 µm MG132 and 10 mM N‐Ethylmaleimide (Selleck, #S3692) after washing with PBS. The lysates were then sonicated, boiled, diluted, and centrifuged. The supernatant was incubated with anti‐ZFP36 antibodies and protein A‐Sepharose (Thermo Fisher Scientific, #101090) for 3 h at 4 °C. After washing, the proteins were eluted using 2× SDS sample buffer (Thermo Fisher Scientific, #LC1676), and polyubiquitination was detected by WB using an anti‐ubiquitin antibody (Proteintech, #10201‐2‐AP).

### Dual‐Luciferase Reporter Assays

To assess the effect of ZFP36 on *Pfkfb3* mRNA stability, the *Pfkfb3* 3′ UTR sequence was cloned into a pmirGLO reporter vector (HonorGene, China). To evaluate STAT3's effect on the transcriptional activity of Rmrp, WT or MUT Rmrp promoter sequences were inserted into the pGL3 luciferase reporter vector (HonorGene, China). Cells were cotransfected with reporter vectors and pRL Renilla luciferase control vectors along with ZFP36 or STAT3 overexpression or knockdown constructs. Relative luciferase activity (firefly/Renilla ratio) was measured using a luminometer (GloMax 20/20, Promega Madison, WI, USA) 48 h after transfection.

### ChIP Assay

ChIP assays were performed using a commercial ChIP assay kit (Beyotime, #P2078). Briefly, chromatin was cross‐linked and sonicated into fragments. Chromatin complexes were immunoprecipitated using anti‐STAT3 antibodies or IgG‐coated magnetic beads. Enrichment of the Rmrp promoter region in the purified DNA was determined using qPCR.

### DNA Pull‐Down Assay

Biotinylated DNA probes spanning the *Rmrp* promoter‐binding site for STAT3 were synthesized (HonorGene, China), and streptavidin‐coated magnetic beads were prepared. The magnetic beads were washed with the binding buffer and coupled with biotinylated DNA probes by incubation at room temperature. The unbound probe was removed with a washing buffer to obtain the DNA‐magnetic bead complex. The complexes were incubated with cell lysates of MLE‐12 cells in binding buffer for 1 h at 4 °C with rotation. The magnetic beads were subsequently washed with wash buffer to remove nonspecific binding proteins. Finally, the binding proteins were eluted with SDS‐containing elution buffer, and the eluate was collected for WB.

### Bacterial Counts

Bacterial loads in the lungs and blood samples were measured as previously described.^[^ ^78^ ^]^ Lung samples were perfused with PBS containing 5 mM EDTA and homogenized in 1 mL of sterile saline using sonication. The homogenates were serially diluted (1:5), and 10 µL of each dilution was plated on blood agar to determine colony‐forming units (CFUs). Blood samples were collected from the right ventricle of the mice using a sterile heparin syringe and serially diluted (1:2). Similar dilutions were plated on blood agar for bacterial quantification.

### HE Staining

Paraffin sections of lung tissue were prepared, deparaffinized, and rehydrated. The sections were stained with HE and rinsed with distilled water. After dehydration, the sections were mounted with neutral gum. The degree of lung injury was evaluated and scored according to the previously reported criteria.^[^ ^79^ ^]^

### Patient Information and Tissue Specimens

This study included 30 patients with sepsis and 30 patients without sepsis. Patients with sepsis were diagnosed according to the Sepsis‐3 criteria^[^ ^80^ ^]^ and hospitalized at the Second Affiliated Hospital of the University of South China from March 2023 to May 2024. Patients without sepsis, matched for age and sex, underwent bronchoscopy for evaluation of pulmonary nodules detected during routine physical examinations. The exclusion criteria were pregnancy, lactation, use of immunosuppressive drugs, AIDS, cancer, autoimmune diseases, organ transplantation, and blood transfusion within 24 h. The clinical characteristics of the patients are presented in Table S2 (Supporting Information). This study was approved by the Clinical Research Ethics Review Committee of the Second Affiliated Hospital of the University of South China (approval #2024077), and informed consent was obtained from all participants.

BALF and peripheral blood samples were collected from patients with sepsis within 24 h of diagnosis. AMs were isolated from the BALF using flow cytometry, as previously described.^[^ ^73^ ^]^ Briefly, BALF was centrifuged, and the precipitate was resuspended in PBS. Cell suspensions were incubated with anti‐CD45‐AF488 (BioLegend, #304017), anti‐CD11b‐PE/Cy7 (Biolegend, #101216), anti‐HLA‐DR‐APC/Cy7 (BioLegend, #307618), anti‐CD163‐BV421 (Biolegend, #333612), anti‐CD169‐APC (BioLegend, #346008), anti‐CD206‐PE (BioLegend, #321106), and BALF AMs (CD45^+^CD11b^+^HLA‐DR^+^CD163^+^CD169^+^CD206^+^) were sorted using flow cytometry. Blood CD14+ monocytes were isolated using a commercial human CD14+ monocyte‐positive selection kit (IPHASE, #071A105.11) according to the manufacturer's instructions.

### Quantification and Statistical Analysis

GraphPad Prism 7 software was used for statistical analysis and graph generation. The difference between the two groups was assessed using Student's *t*‐test, and differences among three or more groups were analyzed using one‐way ANOVA with Tukey's post hoc test or two‐way ANOVA followed by Sidak's test. Kaplan–Meier survival analysis was performed using the log‐rank test. Pearson correlation analysis was used to evaluate the relationship between two variables. All in vitro experiments were performed in triplicate. For in vivo experiments, n = 6–12 biological replicates per group were used. Data were presented as mean ± SD, and *p* < 0.05 was considered statistically significant.

### Ethics Approval

The study was approved by the Clinical Research Ethics Review Committee of the Second Affiliated Hospital of the University of South China (2024077), and all participants signed an informed consent form. All animal experiments were approved by the Experimental Animal Ethics Committee of the University of South China (2024491) and were performed according to institutional guidelines.

## Conflict of Interest

The authors declare no conflict of interest.

## Author Contributions

Chengxi Liu, Weixia Xuan, Song Cao and Huayun Jia share the co‐first authorship of this work. C.L. and S.C. contributed to formal analysis, investigation, conceptualization, data curation, and wrote, reviewed, and edited the final manuscript. W.L. contributed to resources, validation, investigation, funding acquisition, project administration, supervision, and review. H.J., W.X., Q.W., and X.W. contributed to conceptualization, data curation, and writing of the original draft. Q.W., X.T., L.D., Y.X., M.Z., L.Z., Y.X., J.T., and R.L. contributed to the investigation, methodology, and validation of the original draft. X.Z., X.L., and X.W. contributed to funding acquisition, project administration, supervision, and review. All authors contributed to the article and approved the submitted version.

## Supporting information



Supporting Information

Supporting Information

Supporting Information

## Data Availability

The data that support the findings of this study are available from the corresponding author upon reasonable request.
