# Supplementary material for: Type II Alveolar Epithelial Cells Promote Sepsis‐Induced Immunosuppression in Alveolar Macrophages via Exosomal lncRNA Rmrp Release
Source: Adv Sci (Weinh). 2025 Nov 3;13(1):e00376. doi: 10.1002/advs.202500376 (PMC12766999; doi:10.1002/advs.202500376)
Supplement: Supplementary file 1 — Supporting Information [file ADVS-13-e00376-s001.docx]

Supporting Information

Type II Alveolar Epithelial Cells Promote Sepsis-Induced Immunosuppression in Alveolar Macrophages via Exosomal lncRNA Rmrp Release

Chengxi Liu^1#^, Weixia Xuan^2#^, Song Cao^3#^, Huayun Jia^4#^, Qian Wu^5^, Xiaowu Tan^6^, Qijie Wang^7^, Xiaojun Li^4^, Lisha Ding^4^, Yaru Xiong^4^, Meiyun Zhao^6^, Longcheng Zheng^2^, Yunzhu Xi^6^, Jianhua Tan^6^, Rong Li^6^, Xulong Zhang^8*^, Wenjie Liu^1*^, Xu Wu^6*^


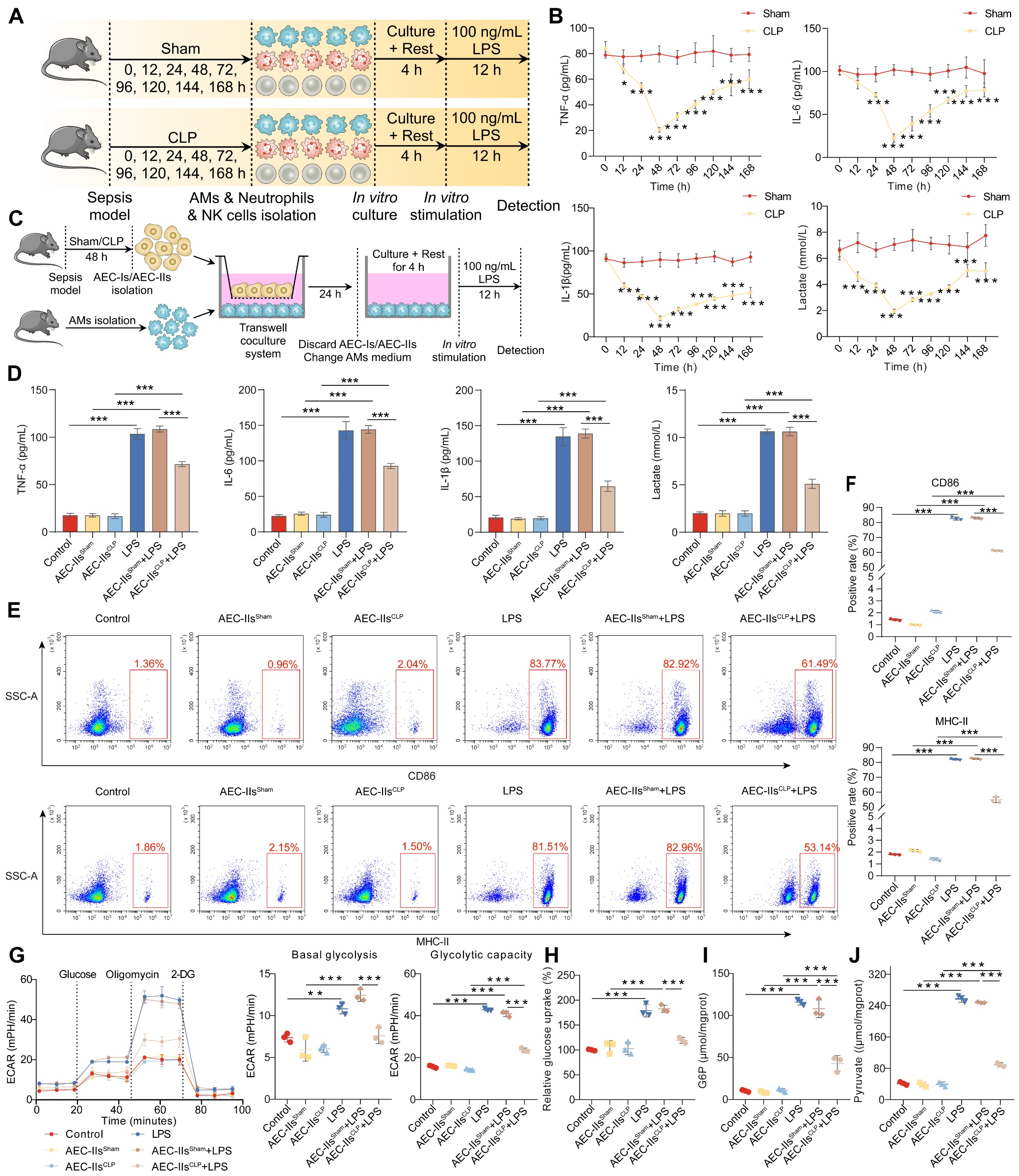


**Figure S1. AEC-IIs inhibits immune responses and glycolysis of AMs post sepsis.** (A) Schematic overview of experimental design for panels S1B and S3. (B) ELISA was used to detect the concentrations of TNF-α, IL-6, IL-1β, and lactate in the supernatant of AMs post LPS stimulation (n = 6/group). (C) Treatment schematic for panels S1D–J and S4. (D) ELISA was used to assess the supernatant TNF-α, IL-6, IL-1β, and lactate contents of AMs after coculture with AEC-IIs and subsequent LPS treatment (n = 3/group). (E) Expression of CD86 and MHC-II in AMs were analyzed using flow cytometry. Representative flow cytometry plots are shown. (F) Percentages of CD86^+^ and MHC-II^+^ AMs in S1E were determined (n = 3/group). (G) Seahorse extracellular flux analysis was performed to determine the ECAR of AMs after coculture with AEC-IIs and subsequent LPS treatment, and the basal glycolysis and glycolytic capacity were calculated (n = 3/group). (H) Glucose uptake, (I) cellular G6P, and (J) cellular pyruvate levels were determined in AMs after coculture with AEC-IIs and follow-up LPS challenge (n = 3/group). Data are presented as mean ± SD. Two-way ANOVA followed by Sidak’s test (B) or one-way ANOVA followed by Tukey’s test (D, F–J) was used for statistical analysis. **p* < 0.05, ****p* < 0.001.


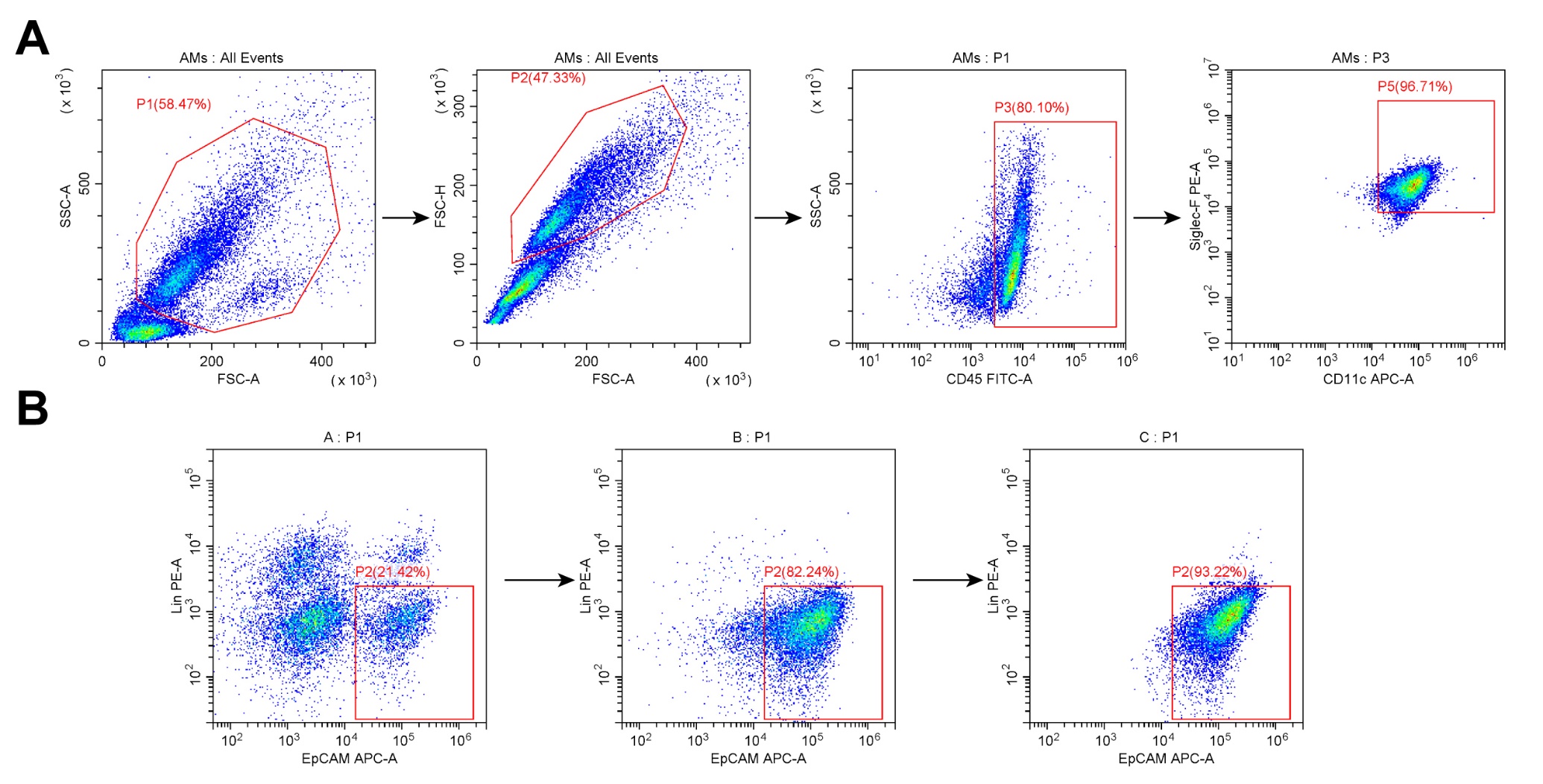


**Figure S2. Flow cytometry sorting strategy.** Flow cytometry sorting strategy for (A) AMs (CD45^+^CD11c^+^SiglecF^+^) and (B) AEC-IIs (Lin^−^EpCAM^+^).


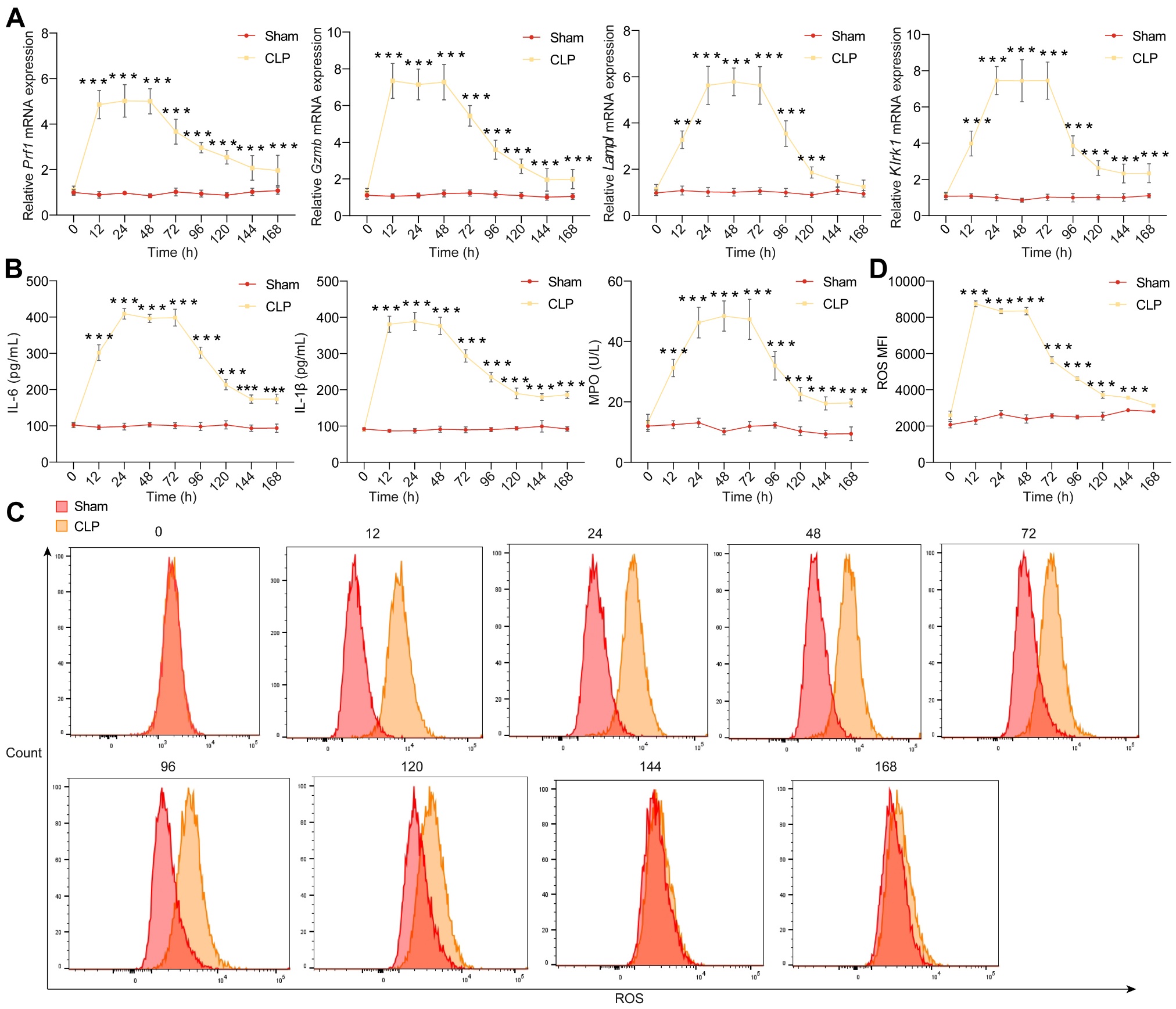


**Figure S3. Immune responses of neutrophils and NK cells against LPS was enhanced after sepsis.** (A) RT-qPCR analysis of *Prf1* (encodes Perforin), *Gzmb* (encodes Granzyme-B), *Lamp1* (encodes CD107a), and *Klrk1* (encodes NKG2D) mRNA levels in NK cells after LPS treatment (n = 9/group). (B) ELISA of IL-6, IL-1β, and MPO in the supernatant of neutrophils post LPS stimulation (n = 6/group). (C) DCFH-DA assay was used to detect the ROS production of neutrophils after treating with LPS. Representative flow cytometry plots are shown. (D) Median fluorescence intensity (MFI) of ROS in S3C were analyzed (n = 3/group). Data are presented as mean ± SD. Two-way ANOVA followed by Sidak’s test was used for statistical analysis. ****p* < 0.001.


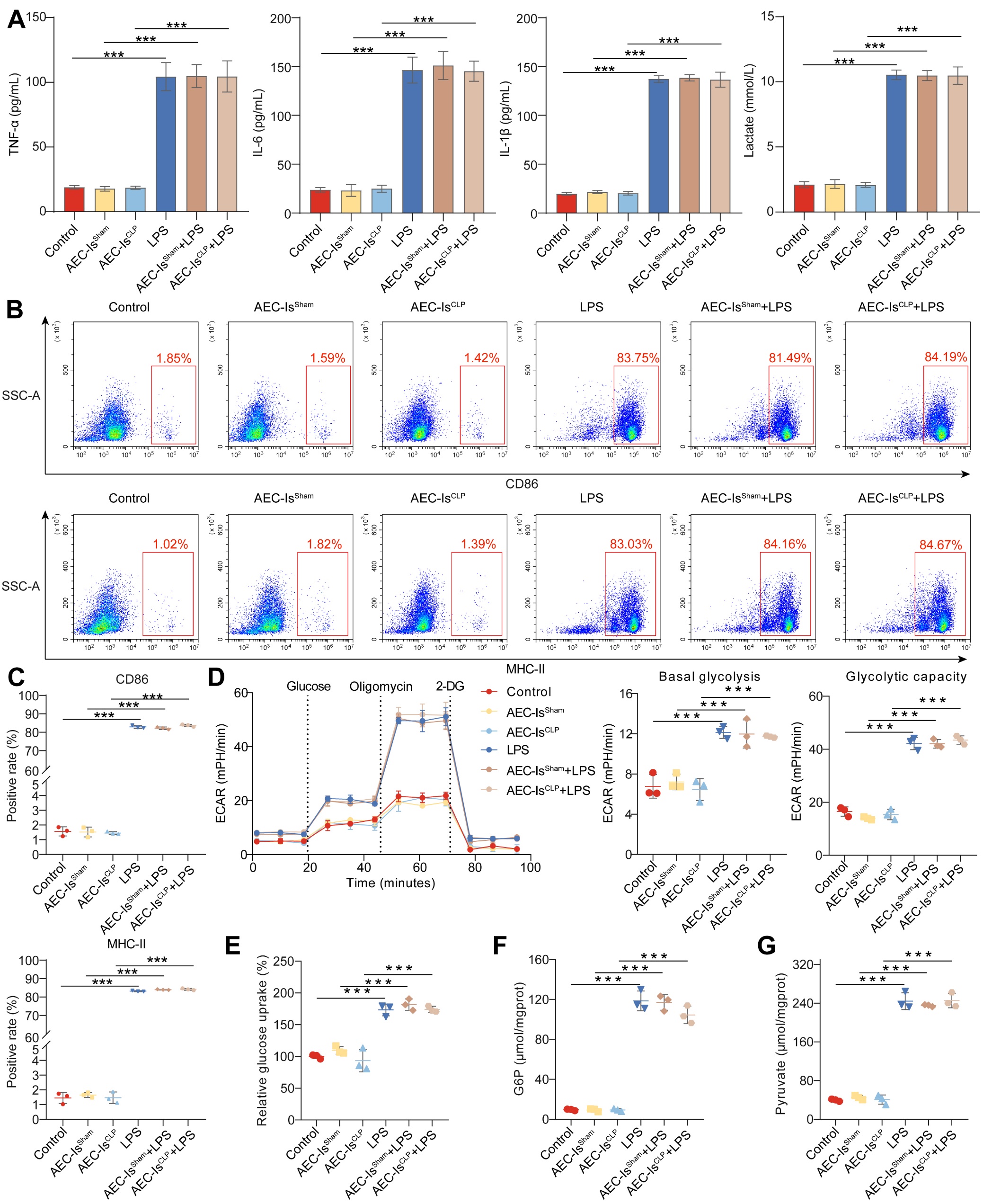


**Figure S4. AEC-Is do not affect the immune responses and glycolysis of AMs.** (A) The supernatant concentrations of TNF-α, IL-6, IL-1β, and lactate of AMs after coculture with AEC-Is and subsequent LPS treatment were determined using ELISA (n = 3/group). (B) Flow cytometry was applied to determine CD86 and MHC-II expression in AMs. Representative flow cytometry plots are presented. (C) Percentages of CD86^+^ and MHC-II^+^ AMs in S4B were evaluated (n = 3/group). (D) Seahorse extracellular flux analysis of ECAR in AMs after coculture with AEC-Is and subsequent LPS stimulation, and the basal glycolysis and glycolytic capacity were assessed (n = 3/group). (E) Glucose uptake, (F) cellular G6P, and (G) cellular pyruvate levels were examined in AMs after coculture with AEC-Is and follow-up LPS treatment (n = 3/group). Data are presented as mean ± SD. One-way ANOVA followed by Tukey’s test was used for statistical analysis. ****p* < 0.001.


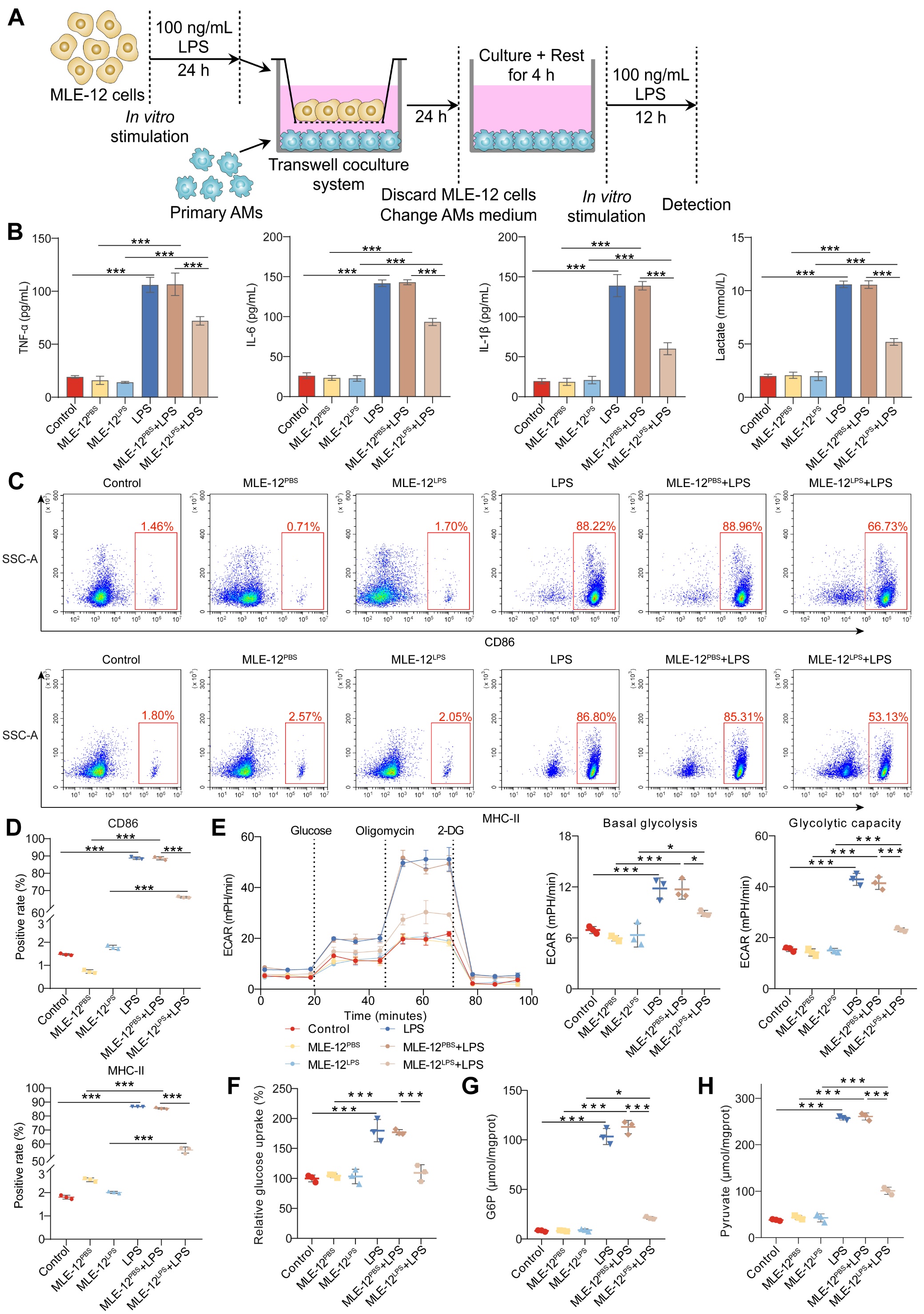


**Figure S5. LPS-stimulated MLE-12 cells impairs the immune responses and glycolysis of AMs.** (A) Experimental scheme for panels S5B–H. (B) ELISA was used to detect the supernatant TNF-α, IL-6, IL-1β, and lactate concentrations of AMs after coculture with MLE-12 cells and follow-up LPS stimulation (n = 3/group). (C) Expression of CD86 and MHC-II in AMs were assessed using flow cytometry. Representative flow cytometry plots are shown. (D) Percentages of CD86^+^ and MHC-II^+^ AMs in S5C were determined (n = 3/group). (E) Seahorse extracellular flux analysis of ECAR in AMs after coculture with MLE-12 cells and follow-up LPS stimulation, and the basal glycolysis and glycolytic capacity were calculated (n = 3/group). (F) Glucose uptake, (G) cellular G6P, and (H) cellular pyruvate levels were evaluated in AMs upon coculture with MLE-12 cells and follow-up LPS challenge (n = 3/group). Data are presented as mean ± SD. One-way ANOVA followed by Tukey’s test was used for statistical analysis. **p* < 0.05, ****p* < 0.001.


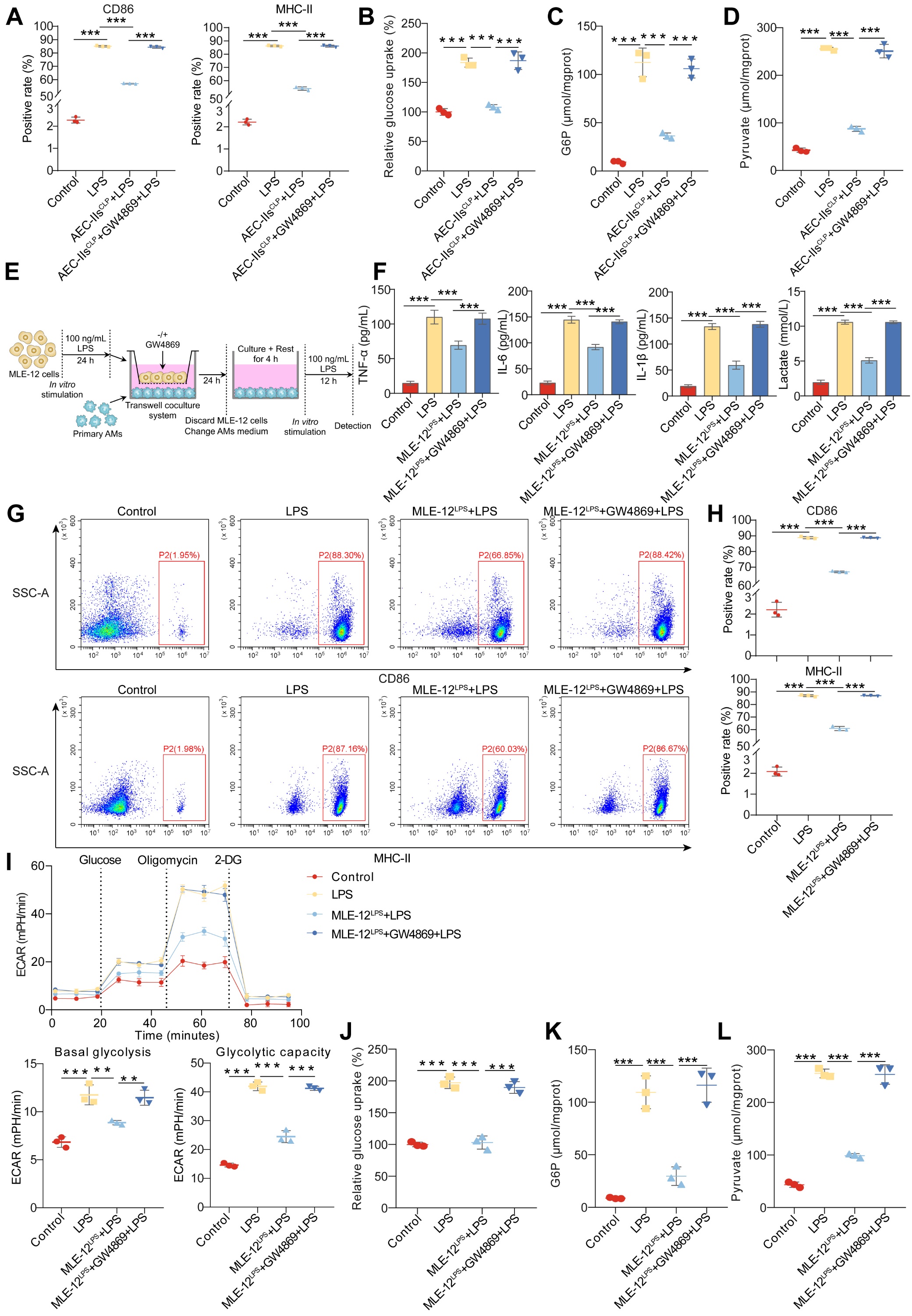


**Figure S6. LPS-stimulated MLE-12 cells defect the immune responses and glycolysis of AMs via exosomes.** (A) Percentages of CD86^+^ and MHC-II^+^ AMs in 1C were analyzed (n = 3/group). (B) Glucose uptake, (C) cellular G6P, and (D) cellular pyruvate levels were determined in AMs after coculture with AEC-IIs and follow-up LPS challenge (n = 3/group). (E) Treatment schematic for panels S6C–L. (F) ELISA for supernatant TNF-α, IL-6, IL-1β, and lactate contents of AMs cocultured with MLE-12 cells and subsequent LPS treatment (n = 3/group). (G) Flow cytometry analysis of CD86 and MHC-II levels in AMs. Representative flow cytometry plots are shown. (H) Percentages of CD86^+^ and MHC-II^+^ AMs in S6G were evaluated (n = 3/group). (I) Seahorse extracellular flux analysis of ECAR in AMs after coculture with MLE-12 cells and subsequent LPS stimulation, and the basal glycolysis and glycolytic capacity were assessed (n = 3/group). (J) Glucose uptake, (K) cellular G6P, and (L) cellular pyruvate levels were examined in AMs after coculture with MLE-12 cells and follow-up LPS treatment (n = 3/group). One-way ANOVA followed by Tukey’s test was used for statistical analysis. ***p* < 0.01, ****p* < 0.001.


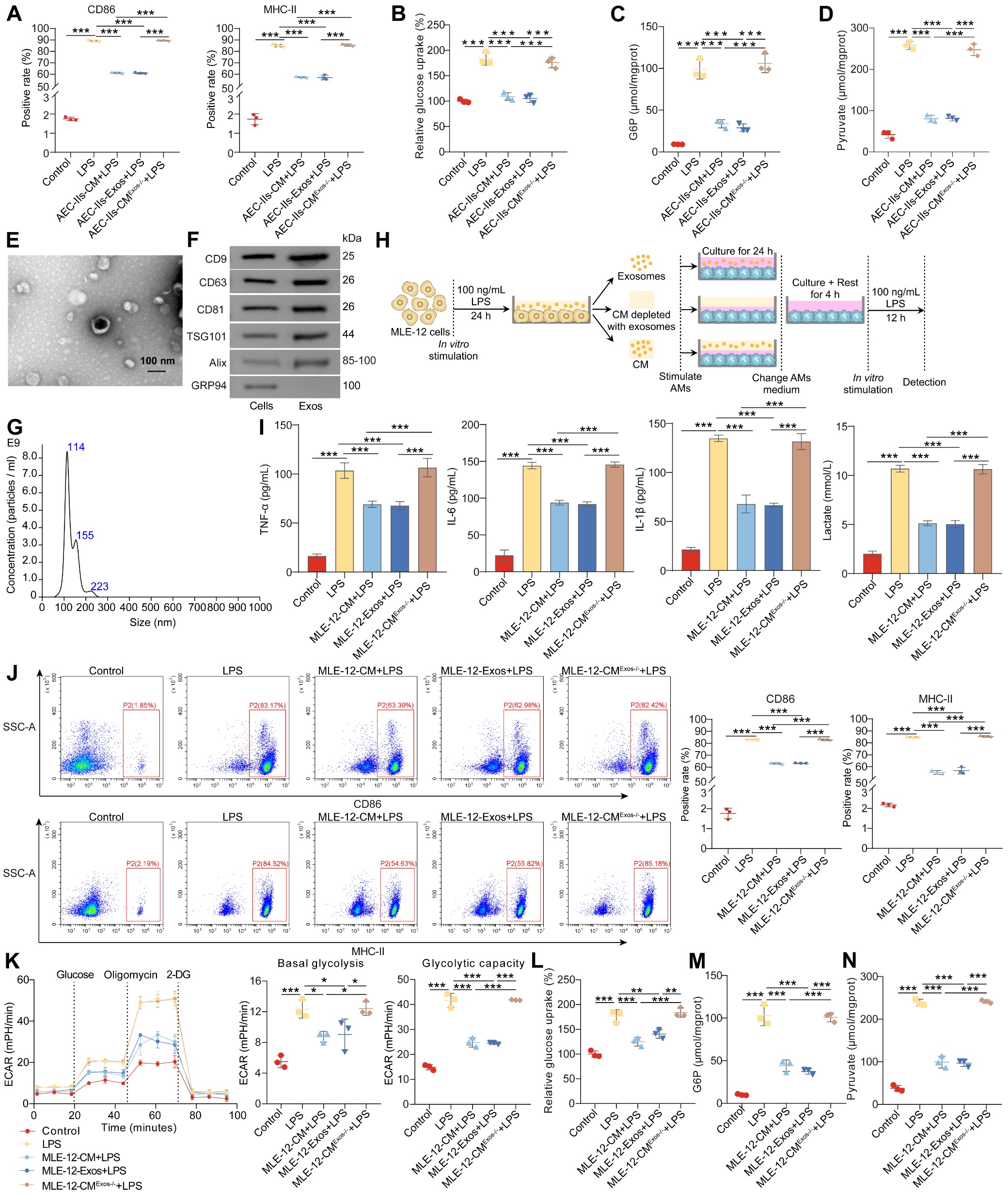


**Figure S7. Exosomes derived from LPS-stimulated MLE-12 cells impair the immune responses and glycolysis of AMs.** (A) Percentages of CD86^+^ and MHC-II^+^ AMs in 1J were determined (n = 3/group). (B) Glucose uptake, (C) cellular G6P, and (D) cellular pyruvate levels were determined in AMs following treatment with AEC-II-CM or AEC-II-derived exosomes (n = 3/group). (E) Representative TEM image of exosomes from MLE-12 cells. Scale bar: 100 nm. (F) WB analysis of exosomal proteins (CD9, CD63, CD81, TSG101, and Alix) and cellular proteins GRP94 in MLE-12 cells or exosomes from MLE-12 cells. (G) NTA analysis of size distribution and the number of MLE-12 cell-derived exosomes. (H) Experimental scheme for panels S7I–N. (I) ELISA for TNF-α, IL-6, IL-1β, and lactate in the supernatant of AMs treated with CM or exosomes from MLE-12 cells (n = 3/group). (J) Levels of CD86 and MHC-II were analyzed in AMs using flow cytometry. Representative flow cytometry plots are shown (left). And the percentages of CD86^+^ and MHC-II^+^ AMs in S7J were determined (right) (n = 3/group). (K) Seahorse extracellular flux analysis of ECAR in AMs treated with CM or exosomes from MLE-12 cells, and the basal glycolysis and glycolytic capacity were assessed (n = 3/group). (L) Glucose uptake, (M) cellular G6P, and (N) cellular pyruvate levels were examined in AMs after treated with CM or exosomes from MLE-12 cells (n = 3/group). Data are presented as mean ± SD. One-way ANOVA followed by Tukey’s test was used for statistical analysis. **p* < 0.05, ***p* < 0.01, ****p* < 0.001.


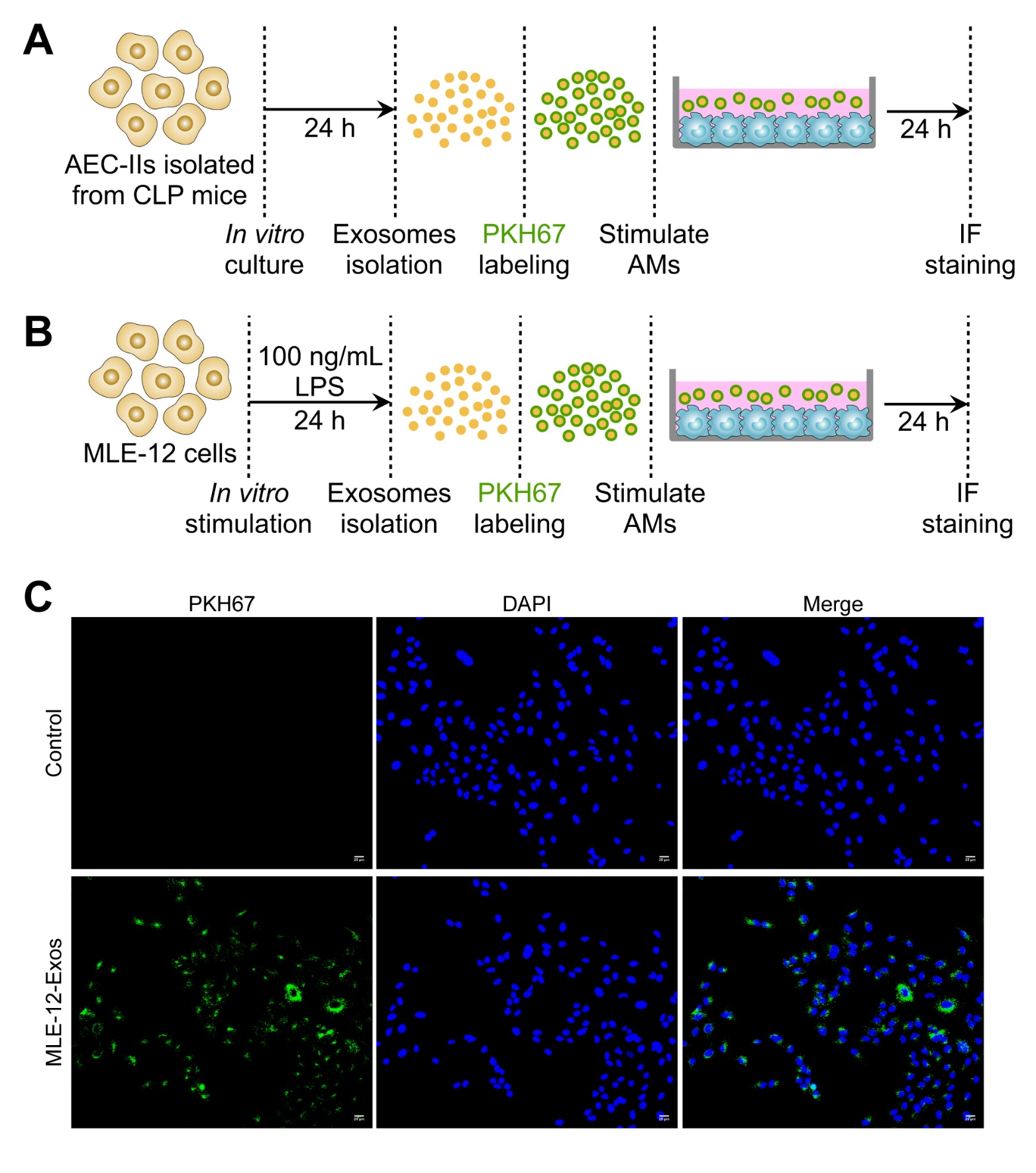


**Figure S8. MLE-12 cell-derived exosomes were internalized by AMs.** (A) Experimental scheme for 1L. (B) Experimental scheme for panel S8C. (C) Representative fluorescent images of AMs incubated with PKH67-labeled exosomes from MLE-12 cells. Scale bar: 25 μm.


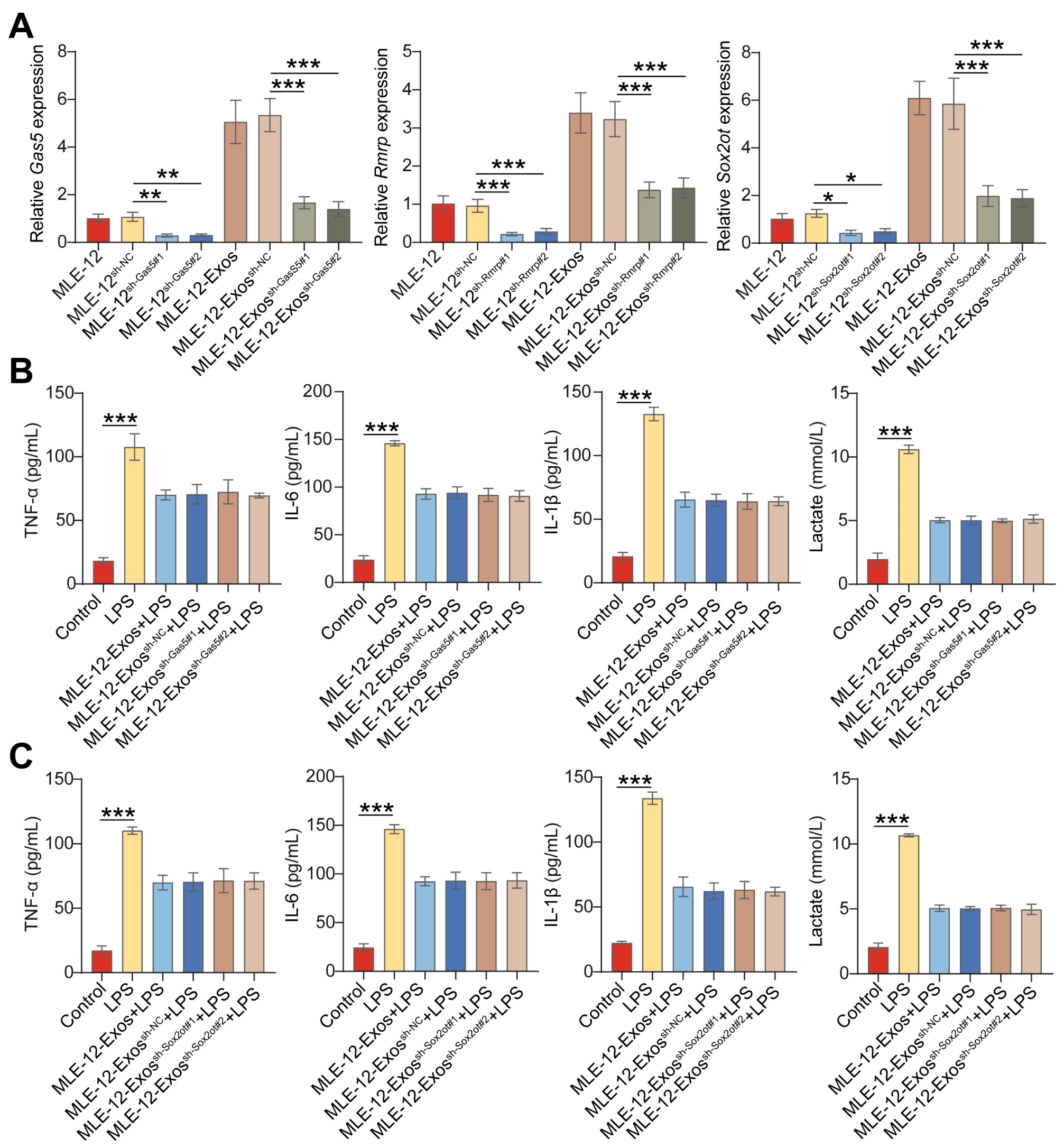


**Figure S9. AEC-II-derived exosomal Gas5 or Sox2ot shows no effect on the immune responses and glycolysis in AMs post sepsis.** (A) RT-qPCR analysis of Gas5, Rmrp, or Sox2ot expression in MLE-12 cells after transfection with gene-specific shRNA (n = 3/group). ELISA for supernatant TNF-α, IL-6, IL-1β, and lactate contents of AMs treated with exosomes from Gas5 (B) or Sox2ot (C) knockdown MLE-12 cells and follow-up LPS stimulation (n = 3/group). Data are presented as mean ± SD. One-way ANOVA followed by Tukey’s test was used for statistical analysis. **p* < 0.05, ***p* < 0.01, ****p* < 0.001.


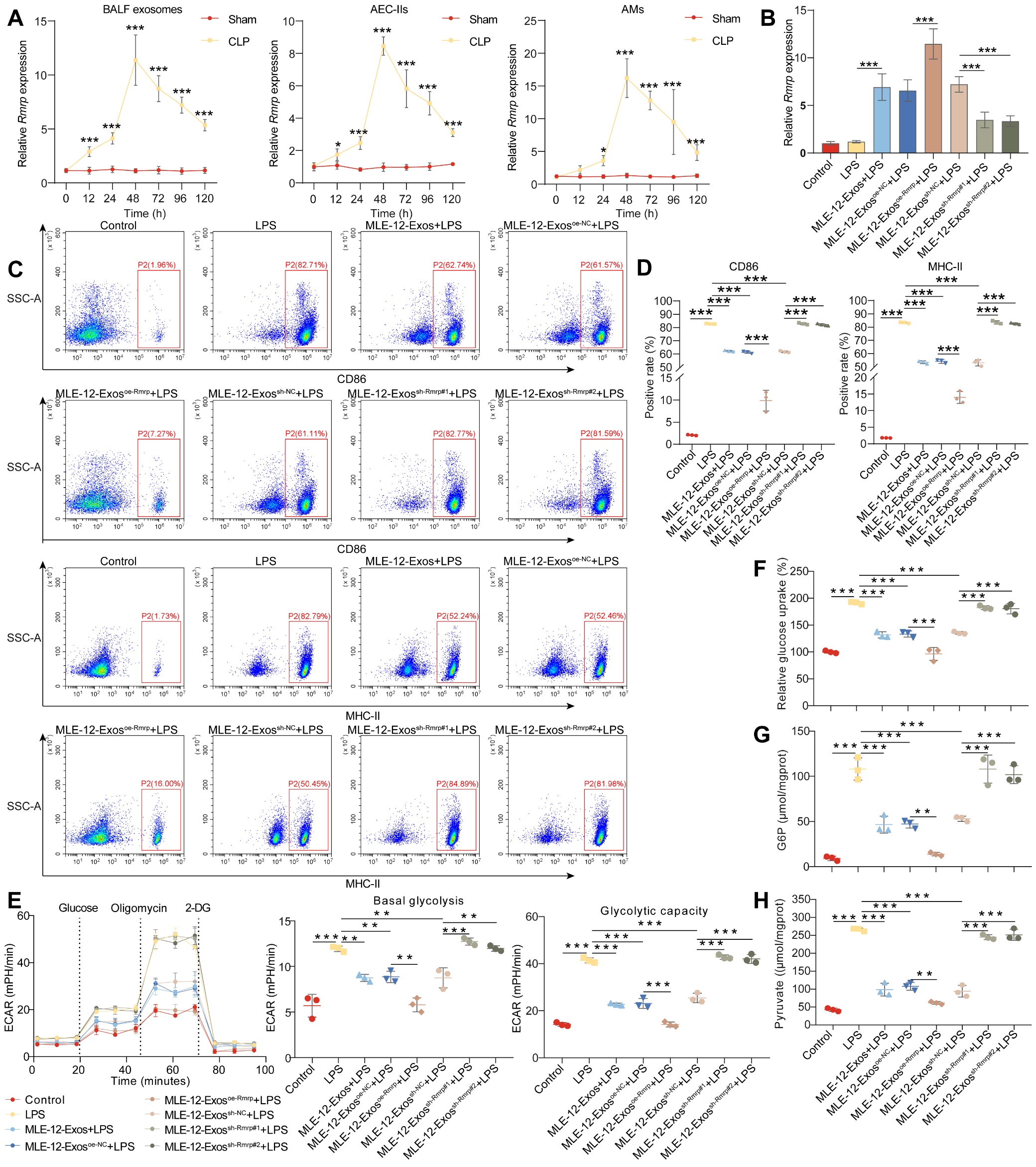


**Figure S10. AEC-II-derived exosomal Rmrp inhibits immune responses and glycolysis of AMs after sepsis.** (A) RT-qPCR analysis of Rmrp in BALF exosomes, AEC-IIs, or AMs extracted from sham or CLP mice (n = 9/group). (B) RT-qPCR analysis of Rmrp level in AMs incubated with MLE-12 cell-derived exosomes (n = 9/group). (C) Flow cytometry analysis of CD86 and MHC-II in AMs. Representative flow cytometry plots are shown. (D) Percentages of CD86^+^ and MHC-II^+^ AMs (n = 3/group). (E) Seahorse extracellular flux analysis was performed to determine the ECAR of AMs after coculture with MLE-12 cell-derived exosomes and subsequent LPS stimulation, and the basal glycolysis and glycolytic capacity were calculated (n = 3/group). (F) Glucose uptake, (G) cellular G6P, and (H) cellular pyruvate levels were determined in AMs after coculture with MLE-12 cell-derived exosomes and follow-up LPS challenge (n = 3/group). Data are presented as mean ± SD. Two-way ANOVA followed by Sidak’s test (A) or one-way ANOVA followed by Tukey’s test (B, D–H) was used for statistical analysis. **p* < 0.05, ***p* < 0.01, ****p* < 0.001.


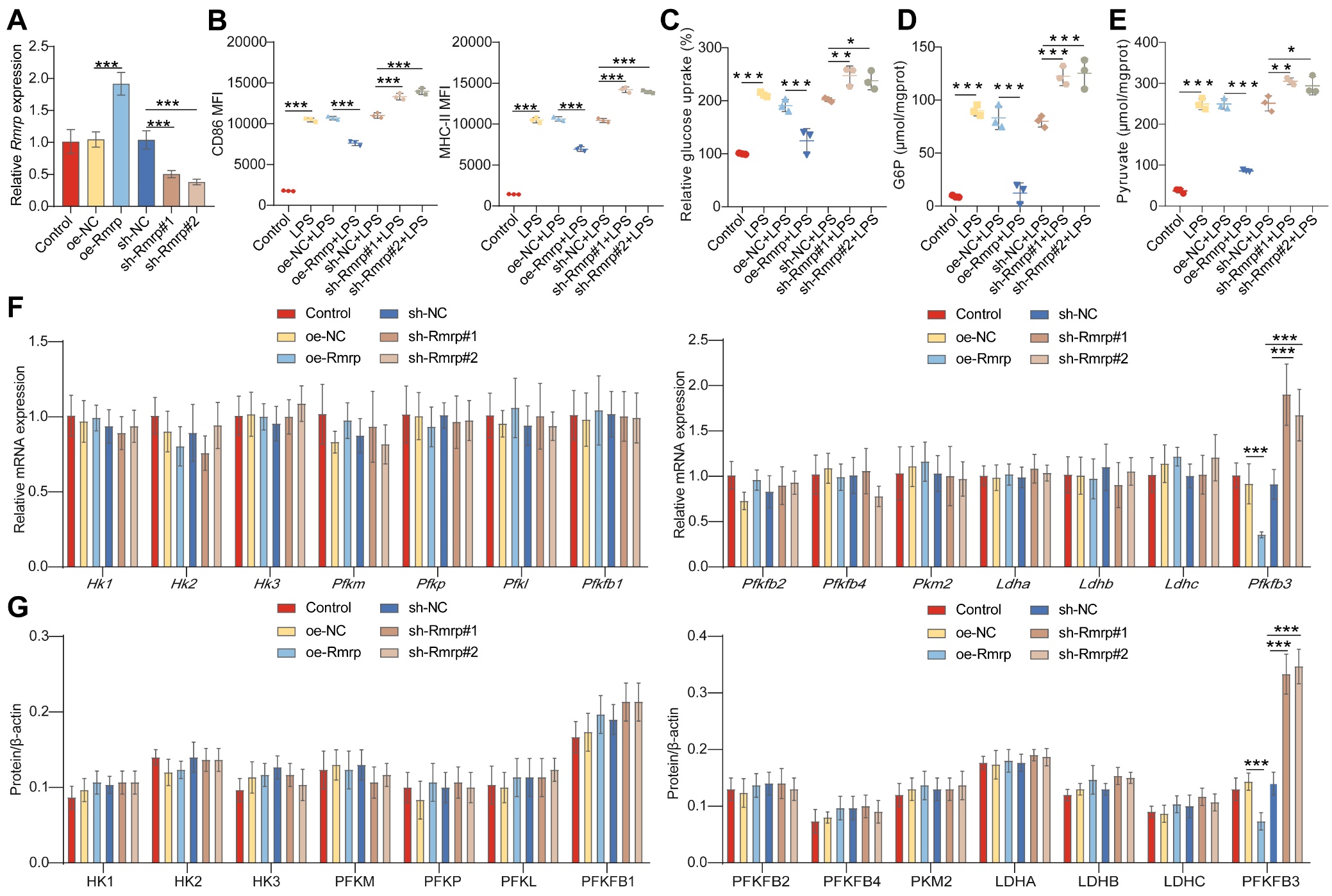


**Figure S11. Rmrp inhibits PFKFB3 expression in MH-S cells.** (A) RT-qPCR analysis of Rmrp in MH-S cells transfected with Rmrp expression vector or Rmrp-specific shRNA (n = 3/group). (B) MFI of CD86 and MHC-II of MH-S cells in 3C were analyzed (n = 3/group). (C) Glucose uptake, (D) cellular G6P, and (E) cellular pyruvate levels were determined in MH-S cells (n = 3/group). (F) RT-qPCR analysis of mRNA levels of glycolytic rate-limiting enzymes in MH-S cells upon Rmrp overexpression or knockdown (n = 3/group). (G) Densitometric analysis of the immunoblot of glycolytic rate-limiting enzymes presented in 3F (n = 3/group). Data are presented as mean ± SD. One-way ANOVA followed by Tukey’s test (A–E) or two-way ANOVA followed by Sidak’s test (F, G) was used for statistical analysis. **p* < 0.05, ***p* < 0.01, ****p* < 0.001.


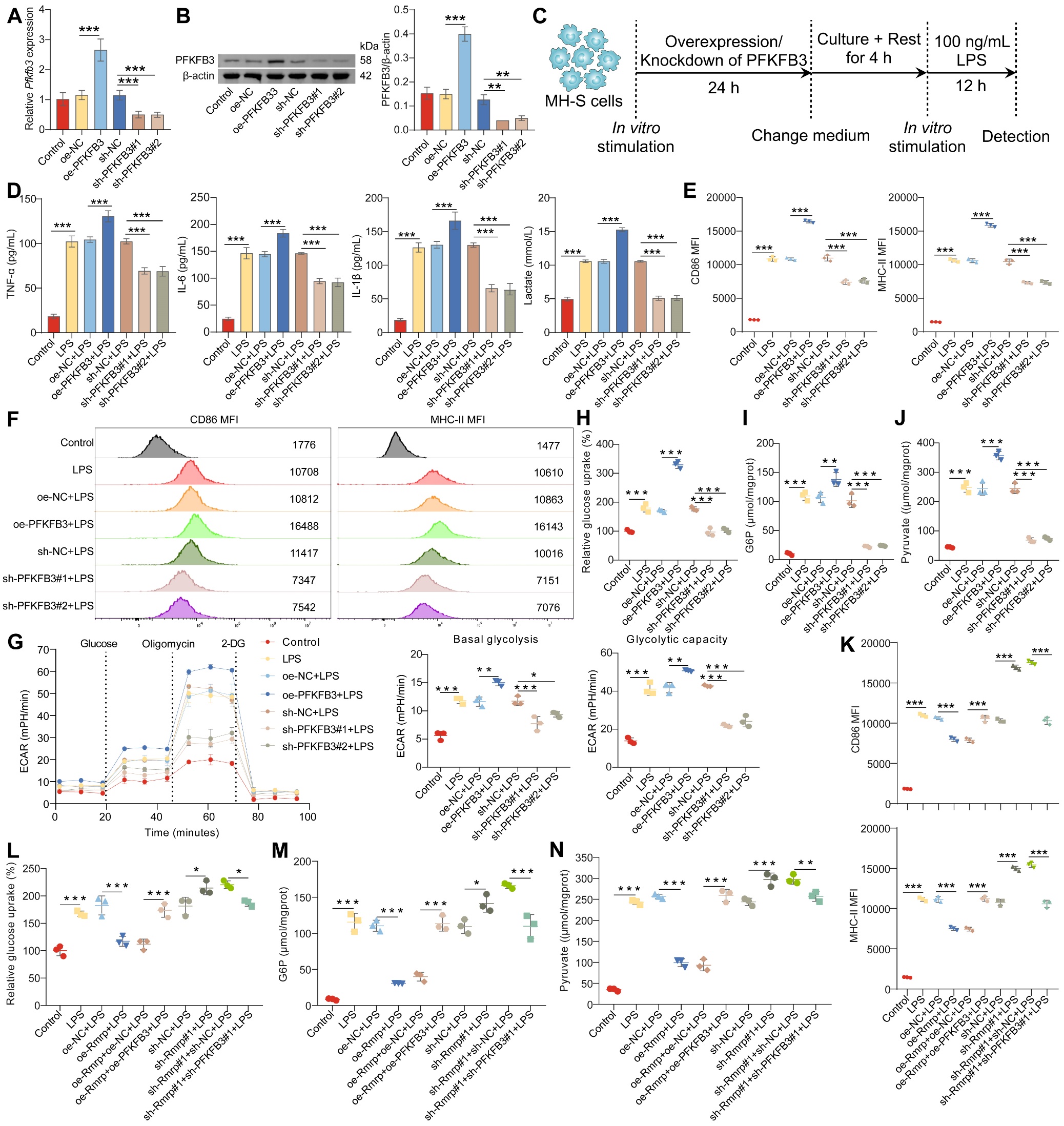


**Figure S12. PFKFB3 maintains glycolysis and immune responses and Rmrp impairs immune responses and glycolysis in MH-S cells by downregulating PFKFB3.** (A) RT-qPCR analysis of mRNA levels and (B) WB analysis of protein levels of PFKFB3 in MH-S cells upon PFKFB3 overexpression or silence (n = 3/group). (C) Experimental scheme for panels S12D–J. (D) ELISA for supernatant TNF-α, IL-6, IL-1β, and lactate contents of MH-S cells after PFKFB3 overexpression or silence and following LPS stimulation (n = 3/group). (E) MFI of CD86 and MHC-II of MH-S cells in S12F were analyzed (n = 3/group). (F) Flow cytometry analysis of CD86 and MHC-II in MH-S cells. Representative flow cytometry plots are presented. (G) Seahorse extracellular flux analysis was performed to determine the ECAR of MH-S cells after PFKFB3 overexpression or silence and following LPS stimulation, and the basal glycolysis and glycolytic capacity were calculated (n = 3/group). (H) Glucose uptake, (I) cellular G6P, and (J) cellular pyruvate levels were determined in MH-S cells (n = 3/group). (K) MFI of CD86 and MHC-II of MH-S cells in 3I were determined (n = 3/group). (L) Glucose uptake, (M) cellular G6P, and (N) cellular pyruvate levels were determined in MH-S cells (n = 3/group). Data are presented as mean ± SD. One-way ANOVA followed by Tukey’s test was used for statistical analysis. **p* < 0.05, ***p* < 0.01, ****p* < 0.001.


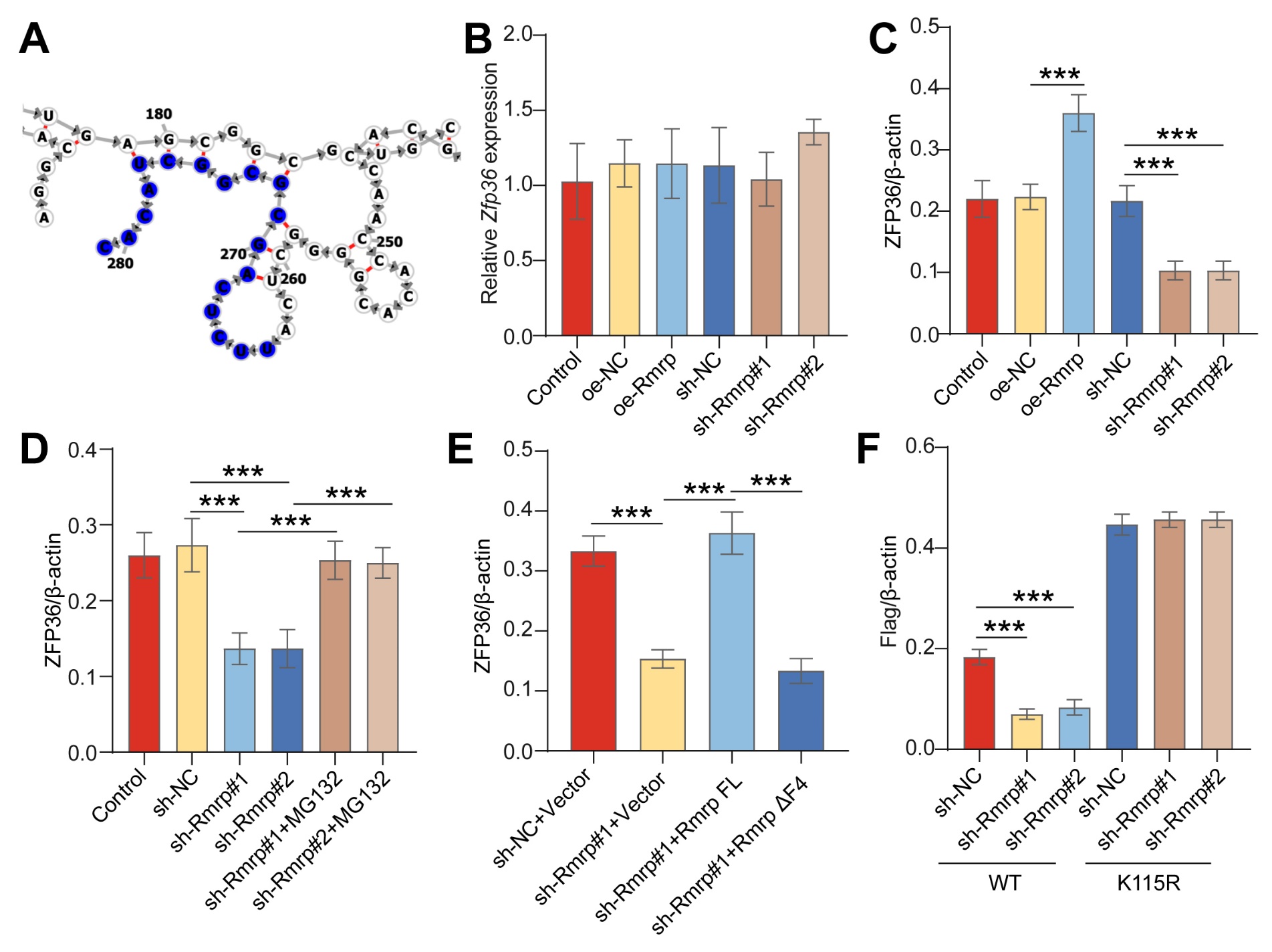


**Figure S13. Rmrp inhibits UPS‑dependent degradation of ZFP36 by binding to it.** (A) AnnoLnc2 database was used to predict the binding sites on Rmrp responsible for interacting with ZFP36. (B) RT-qPCR analysis of mRNA levels of ZFP36 in MH-S cells after Rmrp overexpression or knockdown (n = 3/group). Densitometric analysis of the immunoblot of ZFP36 is shown in (C) 4L, (D) 4N, (E) 4P, and (F) 4S (n = 3/group). Data are presented as mean ± SD. One-way ANOVA followed by Tukey’s test (B–E) or two-way ANOVA followed by Sidak’s test (F) was used for statistical analysis. ****p* < 0.001.


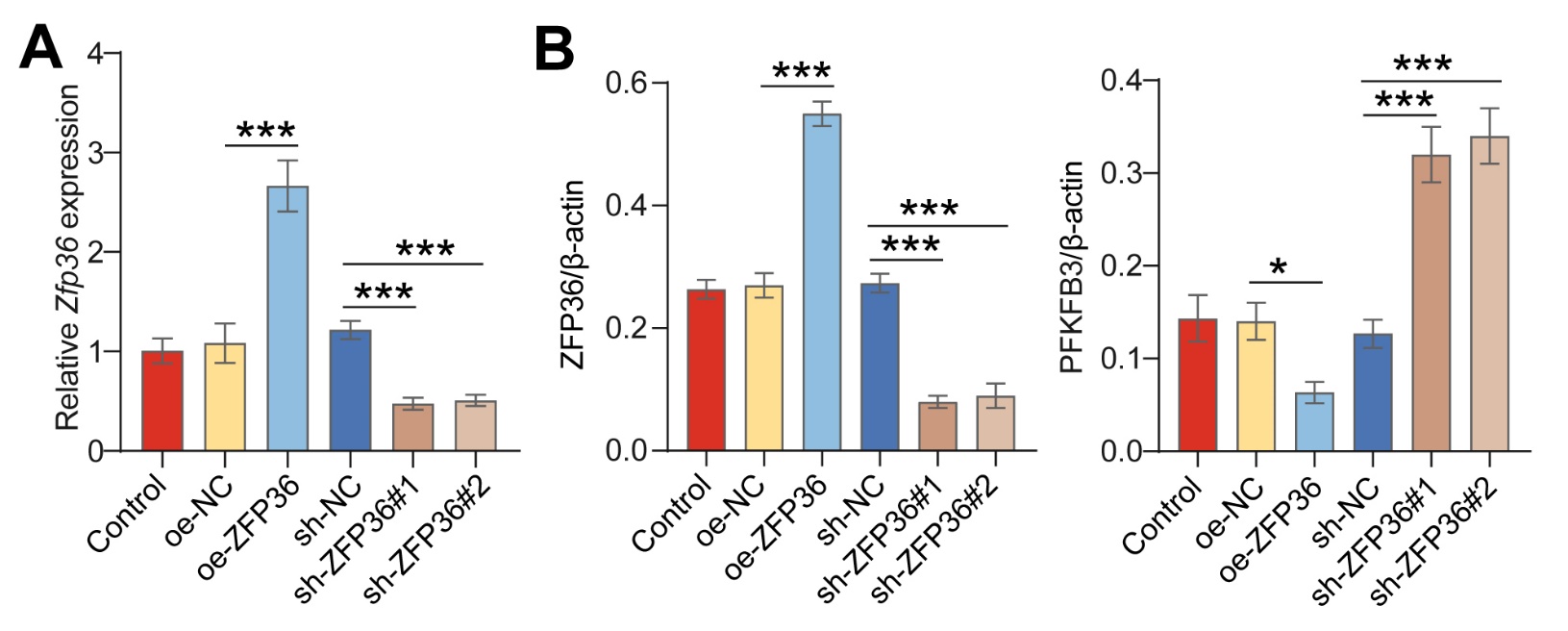


**Figure S14. ZFP36 inhibits PFKFB3 expression in MH-S cells.** (A) RT-qPCR analysis of *Zfp36* mRNA levels in MH-S cells upon ZFP36 overexpression or silence (n = 3/group). (B) Densitometric analysis of the immunoblot of ZFP36 and PFKFB3 is shown in 5E (n = 3/group). Data are presented as mean ± SD. One-way ANOVA followed by Tukey’s test was used for statistical analysis. **p* < 0.05, ****p* < 0.001.


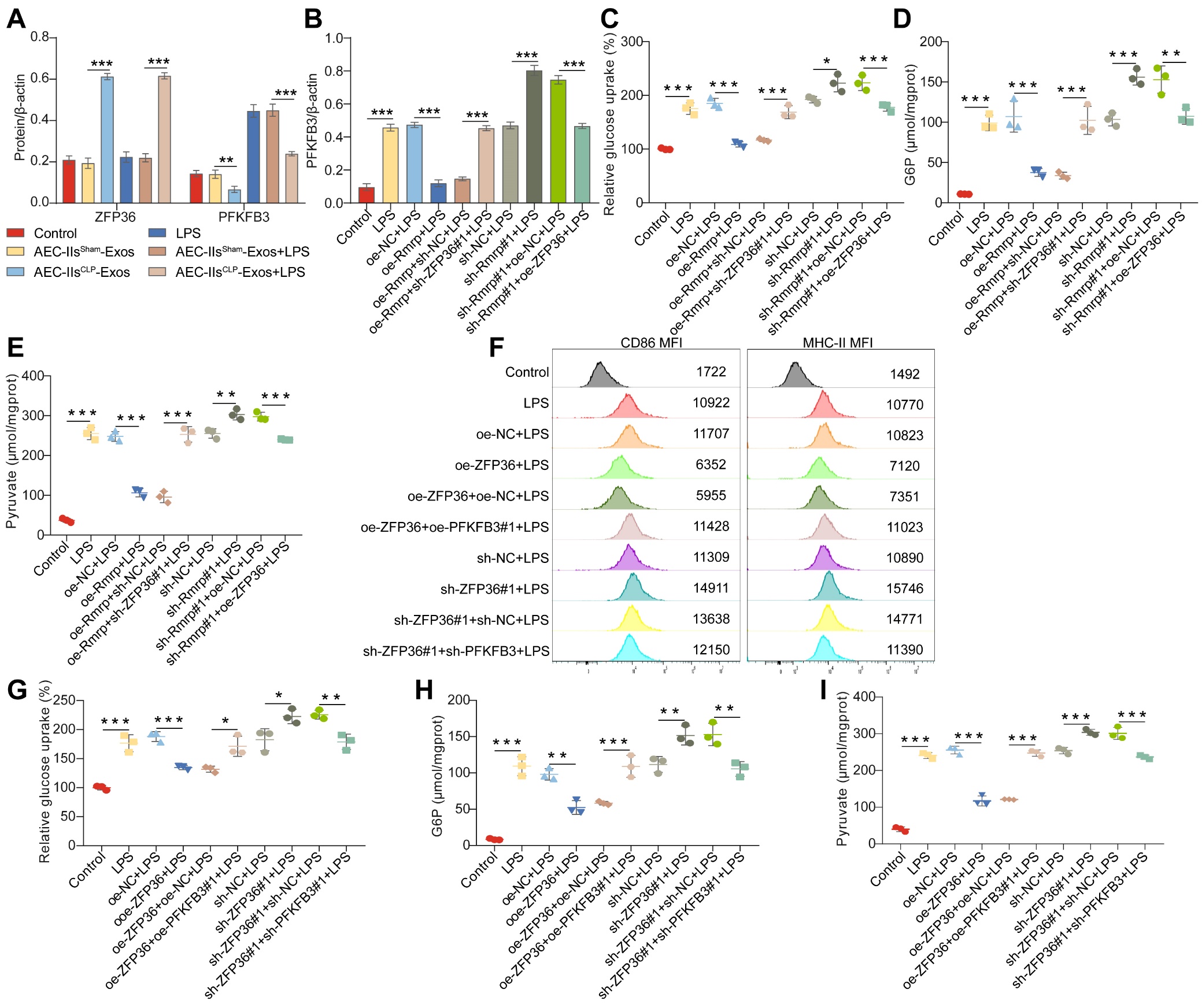


**Figure S15.** Densitometric analysis of the immunoblot of (A) ZFP36 and PFKFB3 is shown in 6C and the (B) PFKFB3 immunoblot is shown in 6F (n = 3/group). (C) Glucose uptake, (D) cellular G6P, and (E) cellular pyruvate levels were determined in MH-S cells (n = 3/group). (F) Flow cytometry was used to measure the expression of CD86 and MHC-II in MH-S cells after ZFP36/PFKFB3 overexpression or knockdown and follow-up LPS stimulation. Representative flow cytometry plots are shown. (G) Glucose uptake, (H) cellular G6P, and (I) cellular pyruvate levels were determined in MH-S cells (n = 3/group). Data are presented as mean ± SD. Two-way ANOVA followed by Sidak’s test (A) or one-way ANOVA followed by Tukey’s test (B–E, G–I) was used for statistical analysis. **p* < 0.05, ***p* < 0.01, ****p* < 0.001.


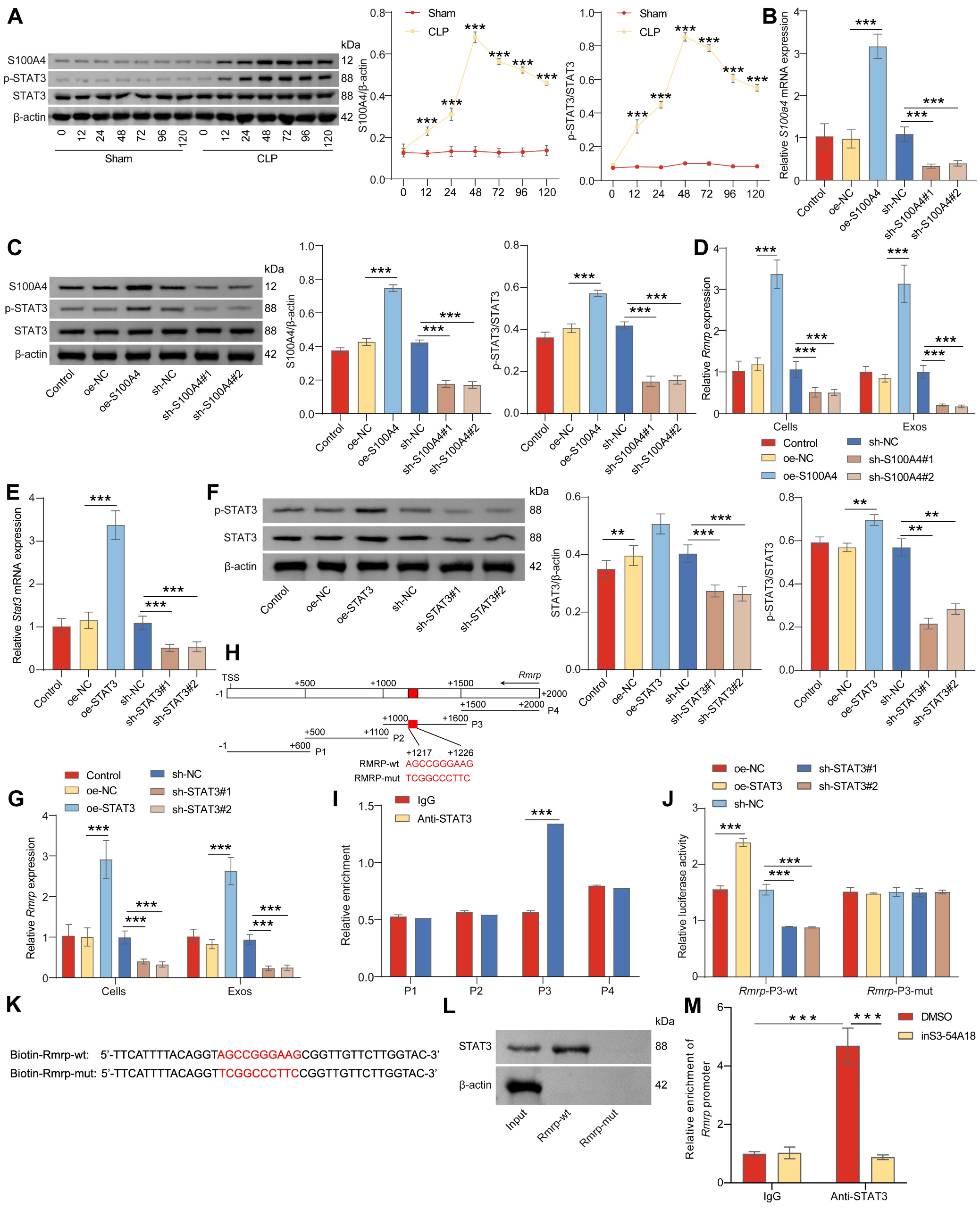


**Figure S16. S100A4/STAT3 axis promotes Rmrp expression in AEC-II-derived exosomes after sepsis.** (A) WB analysis of S100A4 and STAT3 phosphorylation in AEC-IIs isolated from sham or CLP mice (left). Densitometric analysis of the S100A4 (middle) and phosphorylated (p-)STAT3 (right) are shown (n = 3/group). (B) RT-qPCR analysis of *S100a4* mRNA in MLE-12 cells transfected with S100A4 expression vector or S100A4-specific shRNA (n = 9/group). (C) WB analysis of protein levels of S100A4, p-STAT3, and STAT3 in MLE-12 cells upon S100A4 overexpression or silence (left). Densitometric analysis of the immunoblot of S100A4 (middle) and p-STAT3 (right) are shown (n = 3/group). (D) Cellular and exosomal Rmrp levels in MLE-12 cells were detected using RT-qPCR after overexpression or knockdown of S100A4 (n = 9/group). (E) RT-qPCR was applied to detect the mRNA level of STAT3 in MLE-12 cells after STAT3 overexpression or knockdown (n = 9/group). (F) Protein levels of p-STAT3 and STAT3 in MLE-12 cells were measured using WB assay after STAT3 overexpression or silence (left). Densitometric analysis of the immunoblot of p-STAT3 (middle) and STAT3 (right) are presented (n = 3/group). (G) RT-qPCR analysis of cellular and exosomal Rmrp expression in MLE-12 cells upon overexpression or knockdown of STAT3 (n = 9/group). (H) Potential *Rmrp* promoter binding site for STAT3 was predicted using the JASPAR database. (I) ChIP assay was used to measure the enrichment of *Rmrp* promoter region in IgG and anti-STAT3 group (n = 3/group). (J) The relative luciferase activity in MLE-12 cells transfected with the indicated reporters and STAT3 expression vector or STAT3-specific shRNA (n = 3/group). (K) Biotinylated DNA probes spanned the *Rmrp* promoter binding site for STAT3. (L) The DNA–protein complexes were pulled down using streptavidin beads for immunoblot detection for STAT3 and β-actin. (M) MLE-12 cells were treated with DMSO or inS3-54A18. ChIP assay was used to measure the enrichment of *Rmrp* promoter in IgG and anti-STAT3 group (n = 3/group). Data are presented as mean ± SD. Two-way ANOVA followed by Sidak’s test (A, D, G, I, J, M) or one-way ANOVA followed by Tukey’s test (B, C, E, F) was used for statistical analysis. ***p* < 0.01, ****p* < 0.001.


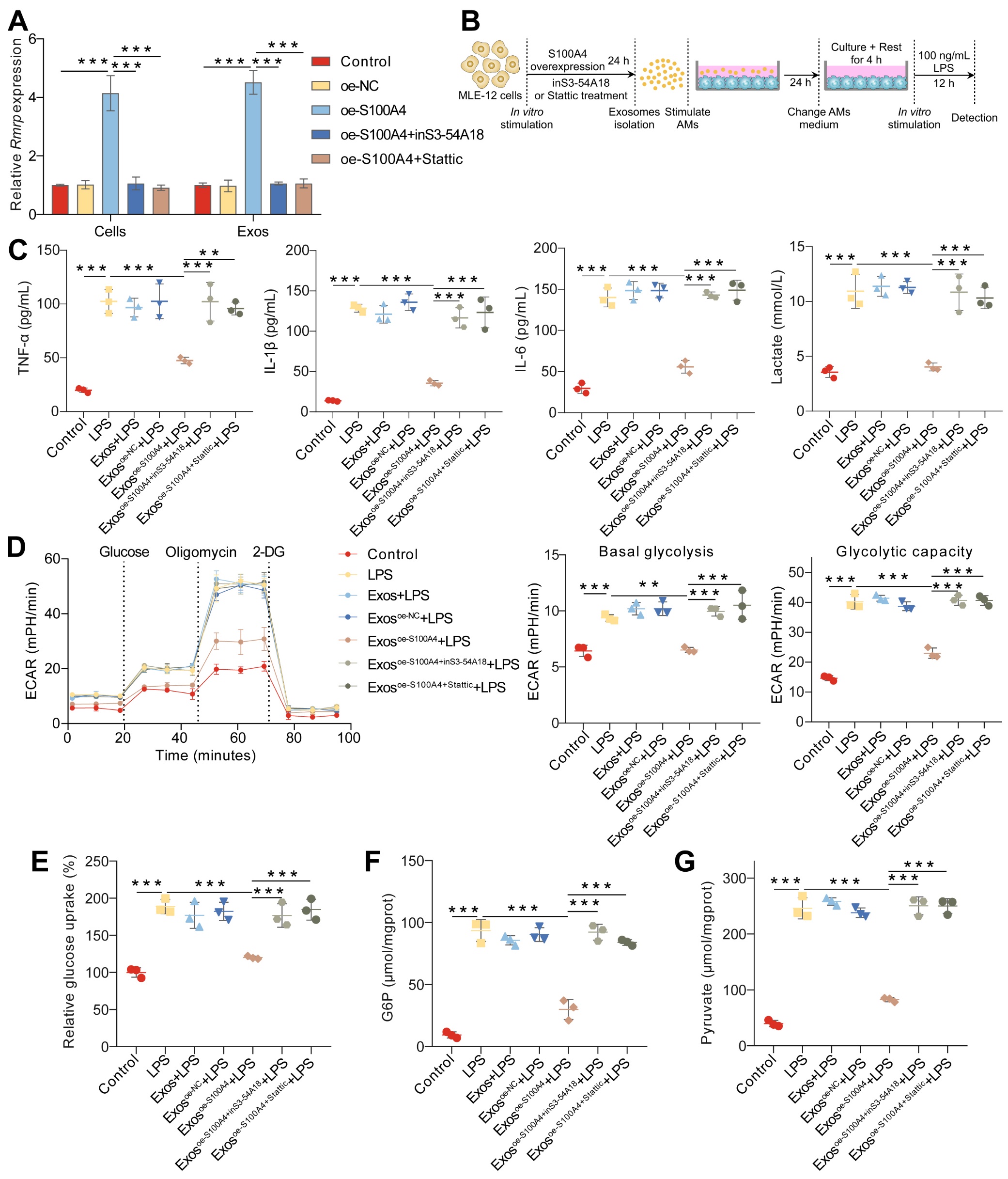


**Figure S17. S100A4 activates STAT3 to upregulate Rmrp and promote immune tolarance and glycolytic defects mediated by AEC-II-derived exosomes.** (A) Cellular and exosomal Rmrp levels in MLE-12 cells were detected using RT-qPCR after S100A4 overexpression and inS3-54A18 or Stattic treatment (n = 3/group). (B) Schematic overview of the experimental design of S17C–G. (C) ELISA of TNF-α, IL-1β, IL-6, and lactate concentrations in the supernatant of AMs after coculture with MLE-12 cell-derived exosomes and subsequent LPS stimulation (n = 3/group). (D) Seahorse extracellular flux analysis was performed to determine the ECAR of AMs (n = 3/group). (E) Glucose uptake, (F) cellular G6P, and (G) cellular pyruvate levels were assessed in AMs after treatment with MLE-12 cell-derived exosomes and follow-up LPS (n = 3/group). Data are presented as mean ± SD. Two-way ANOVA followed by Sidak’s test (A) or one-way ANOVA followed by Tukey’s post hoc test (C–G) were used for statistical analysis. ***p* < 0.01, ****p* < 0.001.


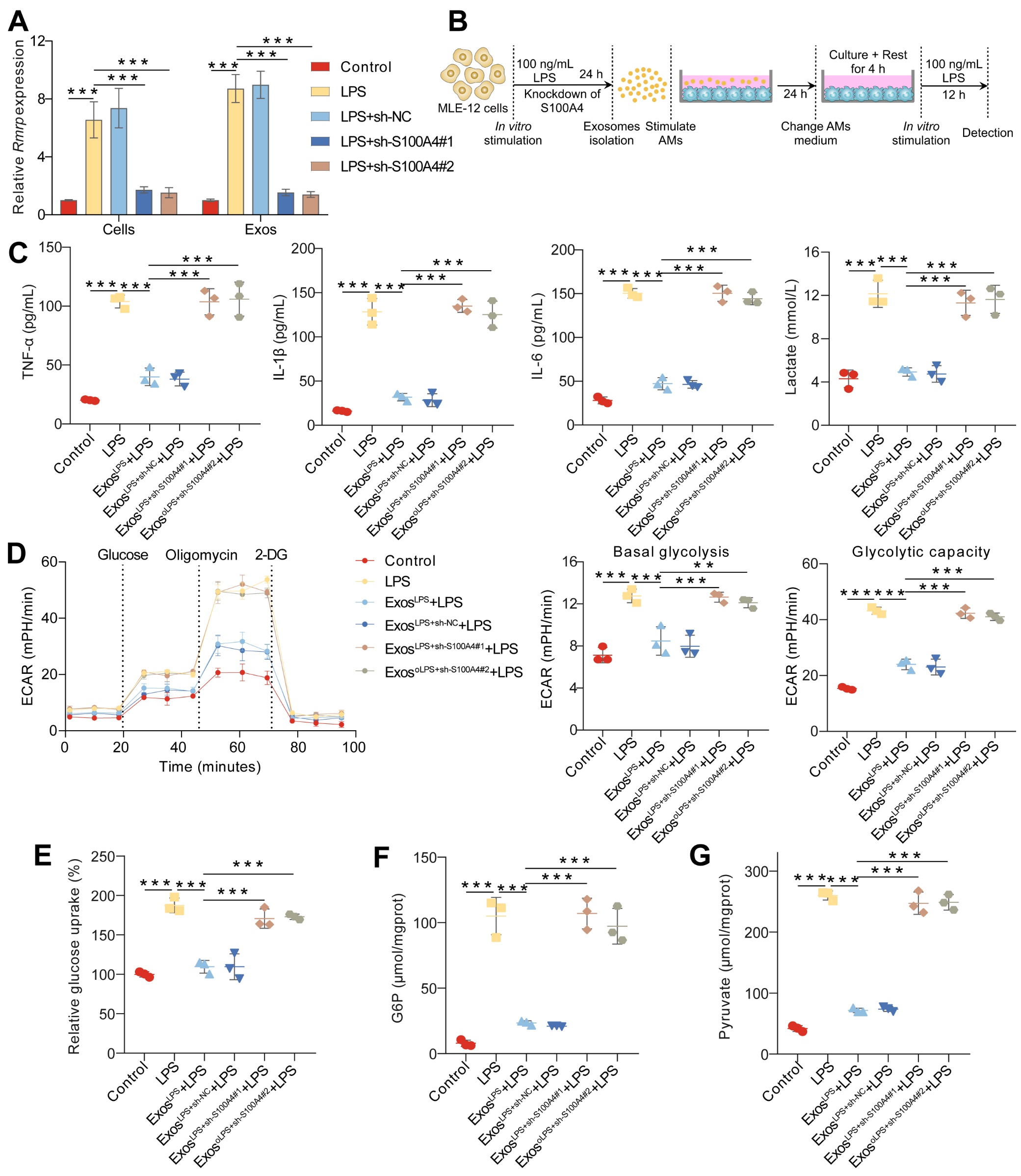


**Figure S18. S100A4 increases Rmrp expression and promotes immune tolarance and glycolytic defects mediated by AEC-II-derived exosomes.** (A) RT-qPCR analysis of cellular and exosomal Rmrp levels in MLE-12 cells after LPS stimulation and S100A4 silencing (n = 3/group). (B) Experimental schematic of S18C–G. (C) ELISA of TNF-α, IL-1β, IL-6, and lactate contents in the supernatant of AMs after treatment with MLE-12 cell-derived exosomes and follow-up LPS (n = 3/group). (D) Seahorse extracellular flux analysis was performed to assess the ECAR of AMs (n = 3/group). (E) Glucose uptake, (F) cellular G6P, and (G) cellular pyruvate contents were analyzed in AMs after coculturing with MLE-12 cell-derived exosomes and subsequent LPS stimulation (n = 3/group). Data are shown as mean ± SD. Two-way ANOVA followed by Sidak’s test (A) or one-way ANOVA followed by Tukey’s post hoc test (C–G) were used for statistical analysis. ***p* < 0.01, ****p* < 0.001.


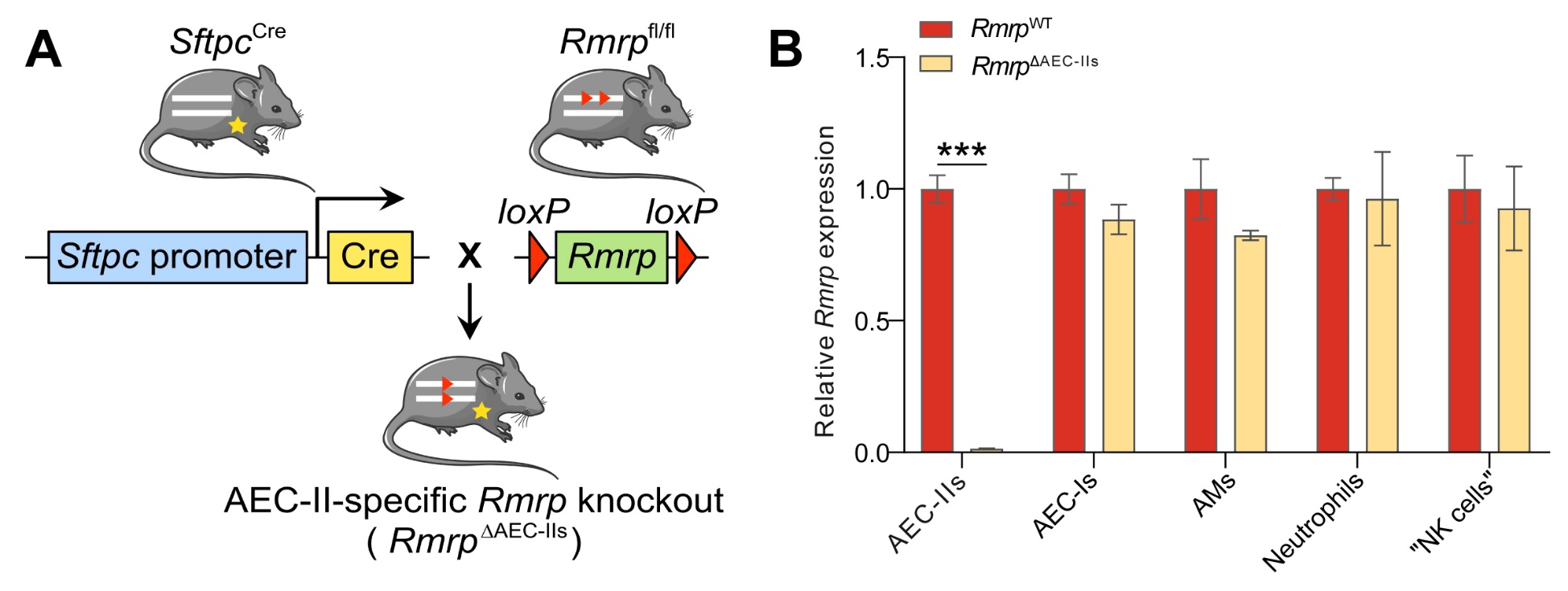


**Figure S19. Establishment and identification of AEC-II-specific *Rmrp* knockout (*Rmrp*^∆AEC-IIs^) mice.** (A) Schematic diagram of AEC-II-specific *Rmrp* knockout (*Rmrp*^∆AEC-IIs^) mice generation. (B) RT-qPCR analysis of Rmrp expression in AEC-IIs, AEC-Is, AMs, neutrophils, and NK cells isolated from *Rmrp*^WT^ or *Rmrp*^∆AEC-IIs^ mice (n = 3/group). Data are presented as mean ± SD. Two-way ANOVA followed by Sidak’s test was used for statistical analysis. ****p* < 0.001.


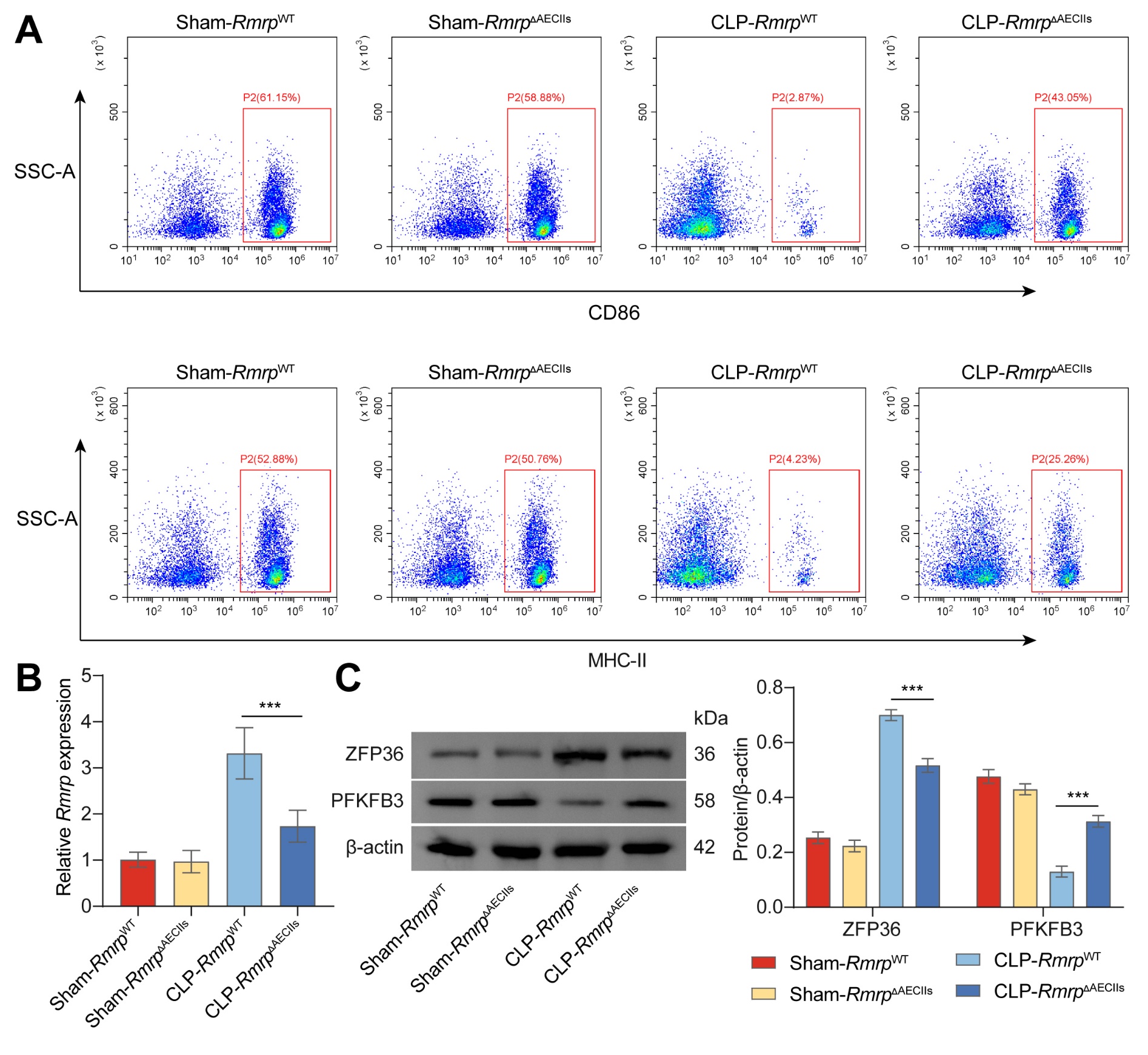


**Figure S20. Rmrp depletion in AEC-IIs leads to stronger immune responses, along with enhanced Rmrp/ZFP36 expression and lower PFKFB3 expression in AMs.** (A) Flow cytometry was used to detect the CD86 and MHC-II expression in AMs isolated from *Rmrp*^WT^ or *Rmrp*^∆AEC-IIs^ mice and subsequently treated with LPS. Representative flow cytometry plots are shown. (B) RT-qPCR analysis of Rmrp (n = 9/group) and (C) WB analysis of ZFP36 and PFKFB3 in AMs exposed to the *in vitro* immune tolerance model (left). Densitometric analysis of the immunoblots of ZFP36 and PFKFB3 are shown (right) (n = 3/group). Data are presented as mean ± SD. One-way ANOVA followed by Tukey’s test (B) or two-way ANOVA followed by Sidak’s test (C) was used for statistical analysis. ****p* < 0.001.


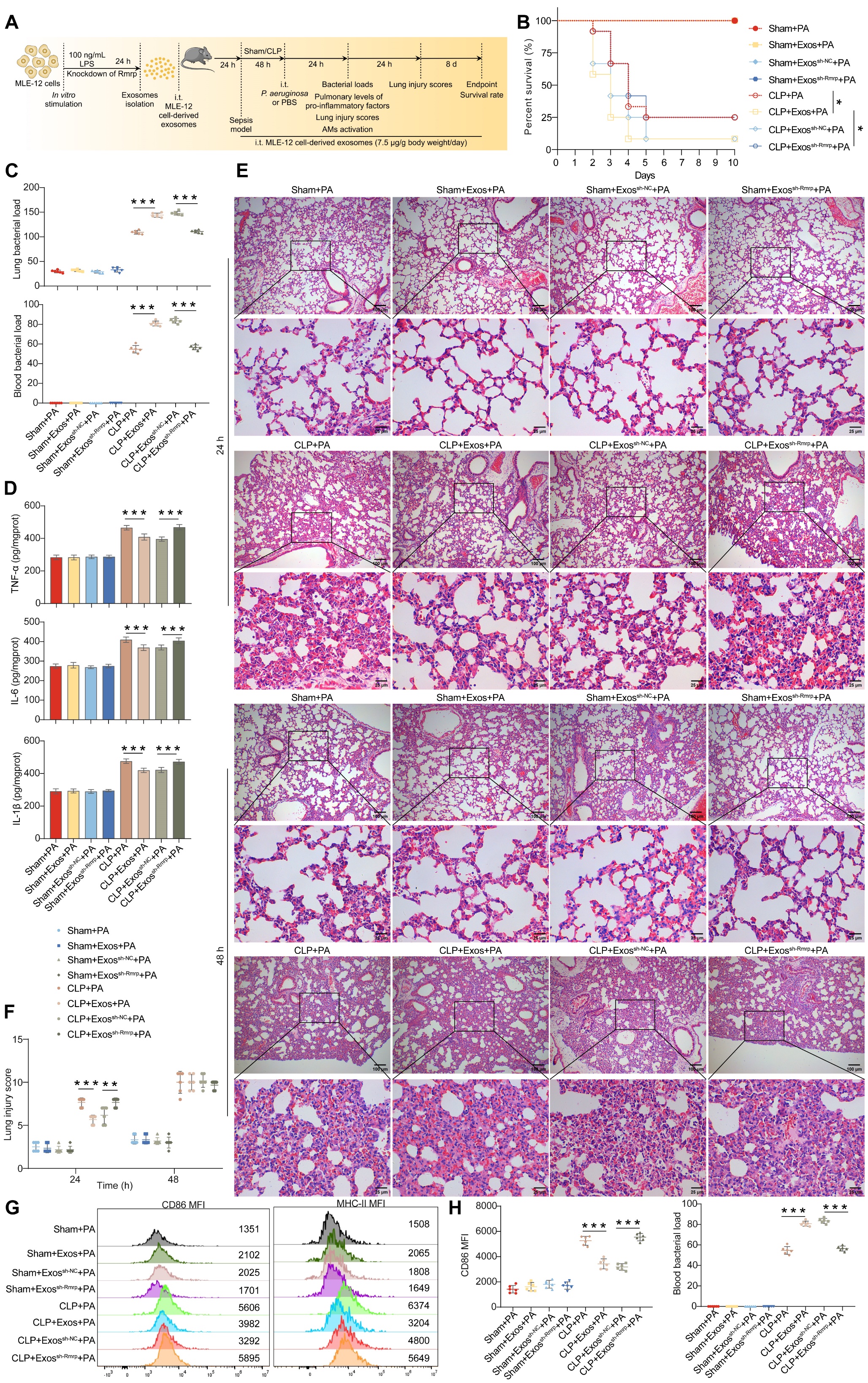


**Figure S21. AEC-II-derived exosomal Rmrp exacerbates immune tolerance of AMs and secondary infection after sepsis.** (A) Schematic overview of experimental design for panels S21B–H. MLE-12 cell-derived exosomes were intratracheal administered to sham and CLP mice, which were then given a secondary infection with *P. aeruginosa* 48 h after CLP. (B) Survival rate of mice was evaluated (n = 12/group). (C) Bacterial loads in the lung and blood (n = 6/group). (D) ELISA was used to assess the concentrations of TNF-α, IL-6, and IL-1β in the lung tissues (n = 6/group). (E) HE staining of lung tissues. Representative HE staining images and (F) lung injury scores are shown (n = 6/group). Scale bars: 100 μm. (G) CD86 and MHC-II levels were detected in AMs using flow cytometry. Representative flow cytometry plots are shown. (H) MFI of CD86 and MHC-II in AMs in S21G were evaluated (n = 6/group). Data are presented as mean ± SD. Log-rank test (B), one-way ANOVA followed by Tukey’s test (C, D, H) and two-way ANOVA followed by Sidak’s test (F) was used for statistical analysis. **p* < 0.05, ***p* < 0.01, ****p* < 0.001.


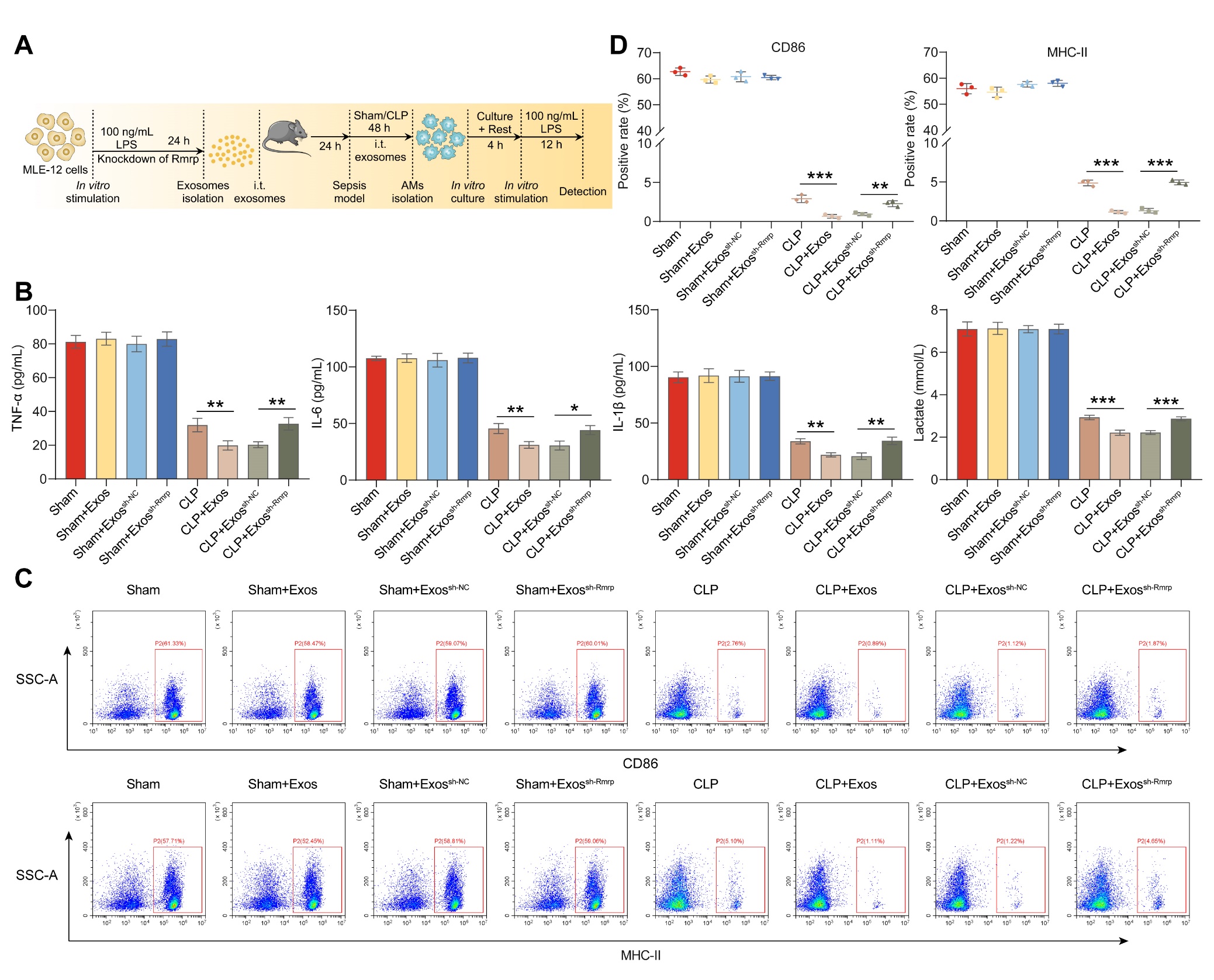


**Figure S22. AEC-II-derived exosomal Rmrp impairs immune functions of AMs.** (A) Experimental scheme for panels S22B–D. (B) Supernatant TNF-α, IL-6, IL-1β, and lactate contents of AMs, which were isolated from mice given MLE-12-cell-derived exosomes and then stimulated with LPS *in vitro* (n = 3/group). (C) Flow cytometry analysis of CD86 and MHC-II in AMs. Representative flow cytometry plots are shown. (D) Percentages of CD86^+^ and MHC-II^+^ AMs in S19C were evaluated (n = 3/group). Data are presented as mean ± SD. One-way ANOVA followed by Tukey’s test was used for statistical analysis. **p* < 0.05, ***p* < 0.01, ****p* < 0.001.


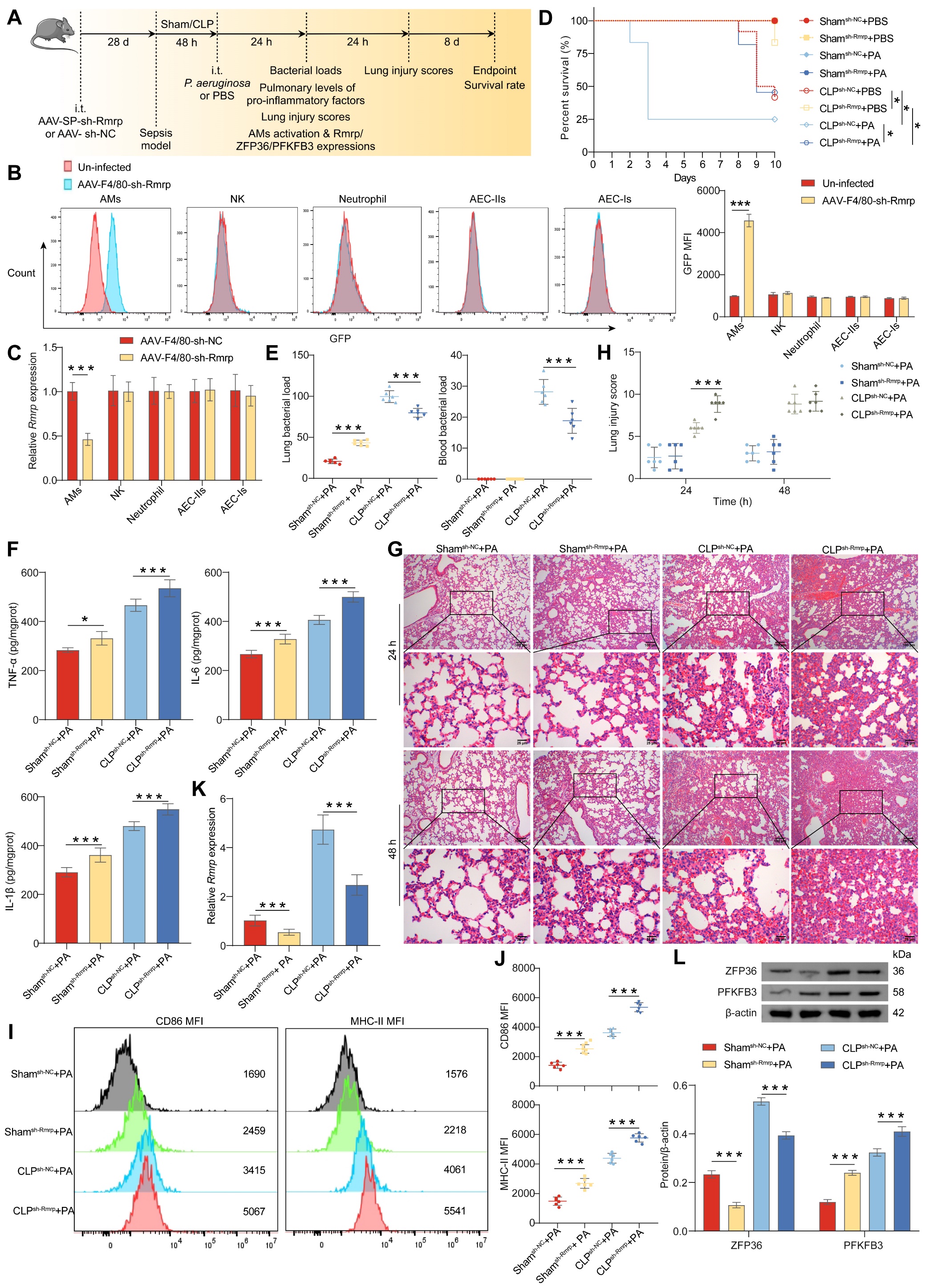


**Figure S23. Specific knockdown of Rmrp in AMs ameliorated SII and secondary pneumonia induced by *P. aeruginosa*.** (A) Schematic overview of experimental design for panels S23D–L. (B) AMs, NK cells, neutrophils, AEC-IIs, and AEC-Is were isolated from mice intratracheally injected with or without AAV-F4/80-sh-Rmrp. Then the GFP expression in these cells were analyzed using flow cytometry. Representative flow cytometry plots are presented (left). Percentages of GFP^+^ cells were evaluated (right) (n = 3/group). (C) Mice were injected intratracheally with AAV-F4/80-sh-NC or AAV-F4/80-sh-Rmrp and AMs, NK cells, neutrophils, AEC-IIs, and AEC-Is were harvested 2 weeks later. RT-qPCR was used to detect the expression of Rmrp in these cells (n = 9/group). (D) Survival rate of mice which were firstly injected intratracheally with macrophage-specific AAV and then challenged with *P. aeruginosa* post CLP (n = 12/group). (E) Bacterial loads in the lung and blood (n = 6/group). (F) Pulmonary concentrations of TNF-α, IL-6, and IL-1β in the lung tissues (n = 6/group). (G) Representative HE staining images of lung tissues and (H) lung injury scores were calculated. Scale bars: 100 μm (n = 6/group). (I) Flow cytometry analysis of CD86 and MHC-II expression in AMs. Representative flow cytometry plots are shown. (J) MFI of CD86 and MHC-II in AMs in S23I were analyzed (n = 6/group). (K) RT-qPCR analysis of Rmrp (n = 9/group) and (L) WB analysis of ZFP36 and PFKFB3 in AMs isolated from mice injected with AAV-F4/80-sh-NC or AAV-F4/80-sh-Rmrp (upper). Densitometric analysis of the immunoblots of ZFP36 and PFKFB3 are shown (lower) (n = 3/group). Data are presented as mean ± SD. Two-way ANOVA followed by Sidak’s test (B, C, H, L), log-rank test (D), or one-way ANOVA followed by Tukey’s test (E, F, J, K) and was used for statistical analysis. **p* < 0.05, ***p* < 0.01, ****p* < 0.001.


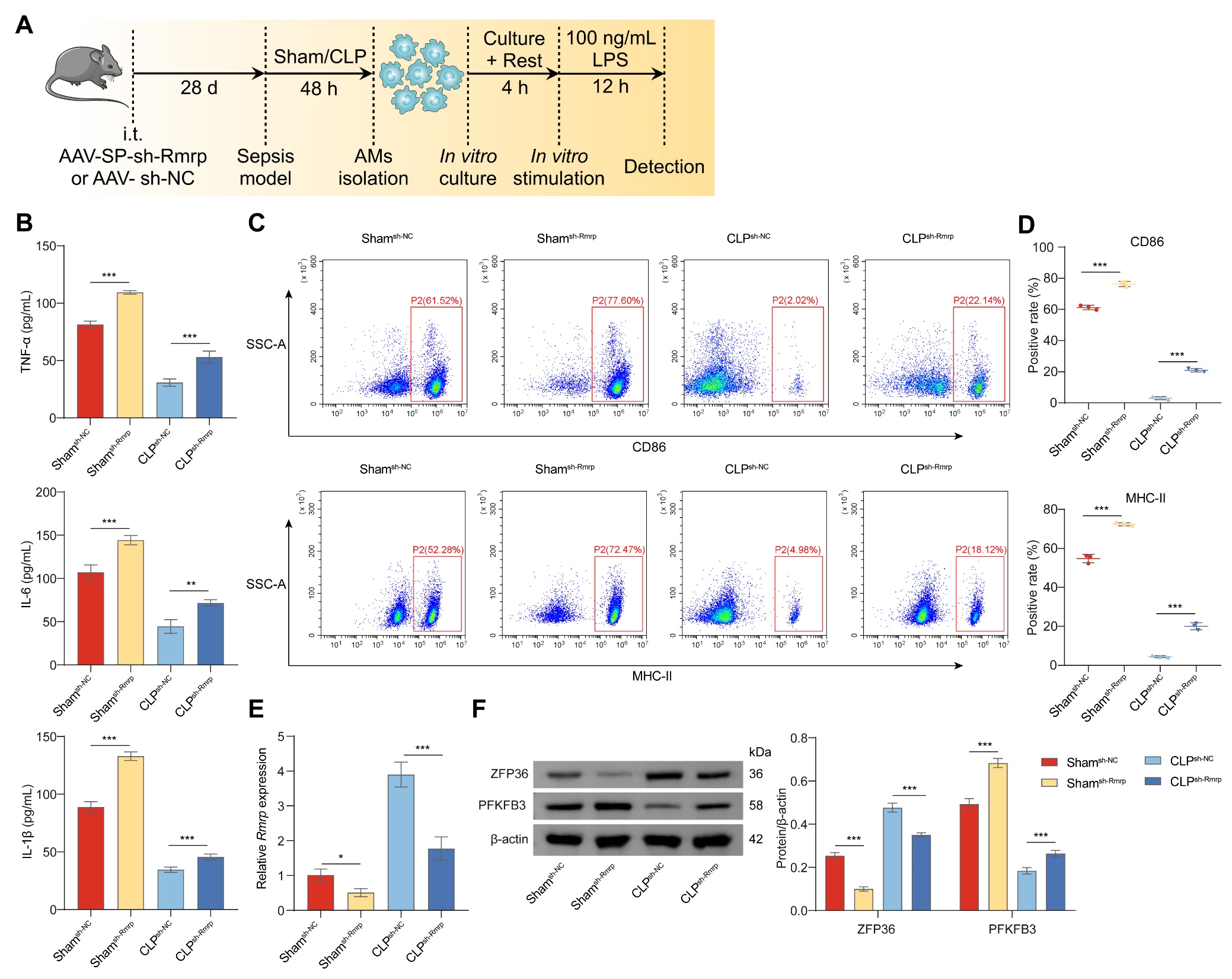


**Figure S24. Specific knockdown of Rmrp in AMs defects immune functions, decreases Rmrp/ZFP36 expression, and increases PFKFB3 expression of AMs.** (A) Experimental scheme for panels S24B–F. (B) ELISA of supernatant TNF-α, IL-6, and IL-1β concentrations of AMs, which is isolated from mice injected with AAV-F4/80-sh-NC or AAV-F4/80-sh-Rmrp and stimulated with LPS *in vitro* subsequently (n = 3/group). (C) CD86 and MHC-II expression of AMs were measured using flow cytometry. Representative flow cytometry plots are shown. (D) Percentages of CD86^+^ and MHC-II^+^ AMs in S24C were evaluated (n = 3/group). (E) RT-qPCR analysis of Rmrp (n = 9/group) and (F) WB analysis of ZFP36 and PFKFB3 in AMs exposed to the *in vitro* immune tolerance model (left). Densitometric analysis of the immunoblot of ZFP36 and PFKFB3 are presented (right) (n = 3/group). Data are shown as mean ± SD. One-way ANOVA followed by Tukey’s test (B, D, E) or two-way ANOVA followed by Sidak’s test (F) was used for statistical analysis. **p* < 0.05, ****p* < 0.001.

**
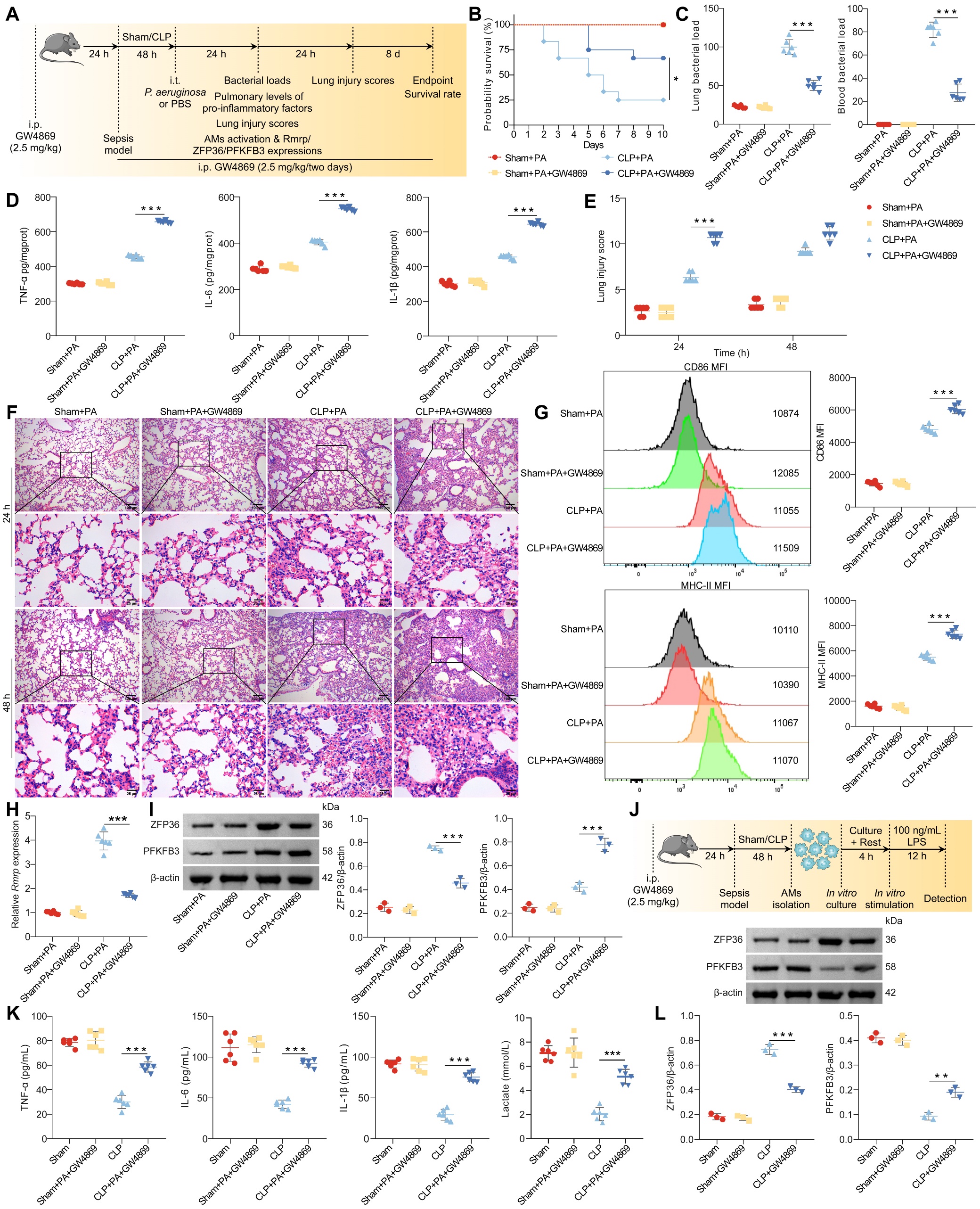
**

**Figure S25. Potential therapeutic role of GW4869 for AM immune tolerance and secondary infections after sepsis.** (A) Schematic overview of the experimental design for panels S25B–I. (B) Survival rates of mice in the sham group or after CLP followed by *P. aeruginosa* challenge (n = 12/group). (C) Bacterial loads in lung (upper) and blood (lower) samples from mice (n = 6/group). (D) Measurement of TNF-α, IL-6, and IL-1β levels in lung tissue samples (n = 6/group). (F) Representative HE staining images of lung tissues. Scale bar: 100 μm (upper) and 25 μm (lower). (E) Lung injury scores based on HE staining (n = 6/group). (G) Flow cytometry analysis of CD86 and MHC-II expression in AMs isolated from mice. Representative flow cytometry plots are shown (left). The MFI of CD86 and MHC-II in AMs are plotted (right) (n = 6/group). (H) RT-qPCR analysis of Rmrp expression in AMs isolated from mice (n = 6/group). (I) WB analysis of ZFP36 and PFKFB3 protein levels in AMs isolated from mice. Densitometric analysis of ZFP36 and PFKFB3 immunoblots is shown (right) (n = 3/group). (J) Schematic overview of the experimental design for panels S25K–L. (K) ELISA of TNF-α, IL-6, IL-1β, and lactate concentrations in the supernatant of AMs isolated from mice and treated with LPS *in vitro* (n = 6/group). (L) WB analysis of ZFP36 and PFKFB3 protein levels in AMs. Densitometric analysis of ZFP36 and PFKFB3 immunoblots is shown (right) (n = 3/group). Data are presented as mean ± SD. Statistical analysis was performed using the Log-rank test (B), one-way ANOVA followed by Tukey's post hoc test (C, D, G–I, K, L), and two-way ANOVA followed by Sidak’s test (F). **p* < 0.05, ***p* < 0.01, ****p* < 0.001.

**Table S1.** Primers used for RT-qPCR analysis.

| Gene | Primer sequences |
| --- | --- |
| Kcnq1ot1 | F 5’-GCACATTCATGCAGCCCAAA-3’ |
|  | R 5’-AGGTGGGTGATAGTTCCAGC-3’ |
| Tug1 | F 5’-TCAGGCCCCTAACTCGAAGA-3’ |
|  | R 5’-GATCGCAAAAGCATAGGCCG-3’ |
| Zfas1 | F 5’-GGCTCGGGGACTACATTTCC-3’ |
|  | R 5’-CAAGTTAACCCCGGAGGGAC-3’ |
| Crnde | F 5’-AGCCAACCTATCAGTGCAGC-3’ |
|  | R 5’-AAAGTGGATCAAGGCGTCCA-3’ |
| H19 | F 5’-GCAGGTAGAGCGAGTAGCTG-3’ |
|  | R 5’-AGACCTGGCCTAGTCTCCAG-3’ |
| Meg3 | F 5’-CAGAGCGCTTCTGAAGACCA-3’ |
|  | R 5’-CACCTACTGGGTGCTCACTG-3’ |
| Snhg1 | F 5’-ACAAAAGGATGGGTGTACGCT-3’ |
|  | R 5’-ATGTTGTCACAGCACCCTGA-3’ |
| Gas5 | F 5’-GTGGGATCTCACAGCCAGTT-3’ |
|  | R 5’-CATTGCGCTCGCTCTGTTAT-3’ |
| Hotair | F 5’-AAGGCTGAAATGGAGGACCG-3’ |
|  | R 5’-TACCGATGTTGGGGACCTCT-3’ |
| Miat | F 5’-CTGGGGGTGATTTCATGCCT-3’ |
|  | R 5’-AGCCATTCATTTCTGCCGGA-3’ |
| Malat1 | F 5’-TGCAGTGTGCCAATGTTTCG-3’ |
|  | R 5’-GGCCAGCTGCAAACATTCAA-3’ |
| Rmrp | F 5’-ACTGTTAGCCCGCCAAGAAG-3’ |
|  | R 5’-CACTGCCTGCGTCACTATGT-3’ |
| Sox2ot | F 5’-CAACTCGTTCTGTCCGGTGA-3’ |
|  | R 5’-CCATGCCAGATCAGGGTGTT-3’ |
| Snhg14 | F 5’-GTTCATCTGTGTGGGGCCTTA-3’ |
|  | R 5’-GCTGTGCAATACCATCAGCA-3’ |
| Neat1 | F 5’-GGTTGACGCCTACACAGTGA-3’ |
|  | R 5’-CTGCTGCCATTCATGCATCC-3’ |
| Pvt1 | F 5’-AGCCTCTGTGGTCTTTAAGCA-3’ |
|  | R 5’-TTTCACAAGCCAGCATTCACT-3’ |
| Xist | F 5’-CTTGGTGGCCAGGATGGAAT-3’ |
|  | R 5’-CAGCCTCGGTCTCTCGAATC-3’ |
| Hk1 | F 5’-GTGATGGTGGTGGTGGTAGG-3’ |
|  | R 5’-CACCCCAAGGAAACACCACT-3’ |
| Hk2 | F 5’-CTGCTTTGGAGATCCGAGGG-3’ |
|  | R 5’-GTCTAGCTGCTTAGCGTCCC-3’ |
| Hk3 | F 5’-TGGGGAGCTGACCCAGAGTT-3’ |
|  | R 5’-AATTGCTGCAAGCATTCCAGTT-3’ |
| Pfkm | F 5’-CTCCCCGTGTTCGCCAAG-3’ |
|  | R 5’-TTTTAGCTCTCCTCCGCCG-3’ |
| Pfkp | F 5’-AACCTGAACACCTATAAGCGTCT-3’ |
|  | R 5’-GCAATCCCGACTCGAACAGCA-3’ |
| Pfkl | F 5’-TCCAGCTACGTGAAGGATGC-3’ |
|  | R 5’-CATACAGGACCGGCTGTGTT-3’ |
| Pfkfb1 | F 5’-GGACTCCGGATCCCATTGAT-3’ |
|  | R 5’-GAAACCGGGAGACATCCGTT-3’ |
| Pfkfb2 | F 5’-GAGCCCTGACTACCCCGAA-3’ |
|  | R 5’-CTCTGGCCTACATTTATCACCT-3’ |
| Pfkfb3 | F 5’-GGGAGAGGTCAGAGAACATGAA-3’ |
|  | R 5’-TGTCTTTGCCACCCCAACAT-3’ |
| Pfkfb4 | F 5’-CGGGAACTGACCCAGAATCC-3’ |
|  | R 5’-AGGCCCACCATGACAATGAG-3’ |
| Pkm2 | F 5’-GCTCTAGGTATCGCAGCAGG-3’ |
|  | R 5’-AGTCCCTGCTTCACTGTGTG-3’ |
| Ldha | F 5’-AACTTGGCGCTCTACTTGCT-3’ |
|  | R 5’-TAGCCGCCTGAGGACTTACT-3’ |
| Ldhb | F 5’-TTCTGCTCGATTCCGCTACC-3’ |
|  | R 5’-ATGCCGTACATTCCCTGTCC-3’ |
| Ldhc | F 5’-AGGTGTGTGTTTTCGGCCTC-3’ |
|  | R 5’-GCAGGCCTACAATGGCTTTC-3’ |
| β-actin | F 5’-ACATCCGTAAAGACCTCTATGCC-3’ |
|  | R 5’-TACTCCTGCTTGCTGATCCAC-3’ |
| Gapdh | F 5’-GCGACTTCAACAGCAACTCCC-3’ |
|  | R 5’-CACCCTGTTGCTGTAGCCGTA-3’ |
| cel-miR-39-3p | F 5’-ACACTCCAGCTGGGTCACCGGGTGTAAATCAG-3’ |
|  | R 5’-CTCAACTGGTGTCGTGGA-3’ |
| Firefly luciferase | F 5’-TCTTGCGTCGAGTTTTCCGG-3’ |
|  | R 5’-GCACGGAAAGACGATGACGG-3’ |
| U6 | F 5’-CTCGCTTCGGCAGCACA-3’ |
|  | R 5’-AACGCTTCACGAATTTGCGT-3’ |

**Table S2.** Clinical characteristics of patients enrolled in this study.

|  | Non-sepsis (n=45) | Sepsis (n=73) | *p* |
| --- | --- | --- | --- |
| Age | 64.53 ± 8.92 | 64.47 ± 8.45 | 0.9671 |
| Gender | 24 Male, 21 Female | 37 Male, 36 Female | - |
| WBC count (×10^9^/L) | 6.98 ± 1.77 | 14.85 ± 3.08 | <0.0001 |
| SOFA scores | - | 8.16 ± 2.20 | - |

**Table S3.** Proteins interacted with sense Rmrp but not anti-sense Rmrp identified using biotin-RNA pulldown assay followed by MS.

**Table S4.** Proteins interacted with sense Pfkfb3 mRNA but not anti-sense Pfkfb3 mRNA identified using biotin-RNA pulldown assay followed by MS.
